# Cost-effectiveness of rotavirus vaccination in children under five years of age in 195 countries: A meta-regression analysis

**DOI:** 10.1016/j.vaccine.2022.05.042

**Published:** 2022-06-21

**Authors:** Mark M. Janko, Jonah Joffe, Danielle Michael, Lauren Earl, Katherine L. Rosettie, Gianna W. Sparks, Samuel B. Albertson, Kelly Compton, Paola Pedroza Velandia, Lauryn Stafford, Peng Zheng, Aleksandr Aravkin, Hmwe H. Kyu, Christopher J.L. Murray, Marcia R. Weaver

**Affiliations:** a Institute for Health Metrics and Evaluation, University of Washington, 3980 15th Ave. NE, Seattle, WA 98195, USA; b Department of Applied Mathematics, University of Washington, Lewis Hall 201, Seattle, WA 98195, USA; c Department of Health Metric Sciences, University of Washington, 3980 15th Ave. NE, Seattle, WA 98195, USA; d Department of Global Health, University of Washington, 3980 15th Ave. NE, Seattle, WA 98195, USA

**Keywords:** Cost-effectiveness analysis, Rotavirus vaccine, Meta-regression

## Abstract

**Background:**

Rotavirus caused an estimated 151,714 deaths from diarrhea among children under 5 in 2019. To reduce mortality, countries are considering adding rotavirus vaccination to their routine immunization program. Cost-effectiveness analyses (CEAs) to inform these decisions are not available in every setting, and where they are, results are sensitive to modeling assumptions, especially about vaccine efficacy. We used advances in meta-regression methods and estimates of vaccine efficacy by location to estimate incremental cost-effectiveness ratios (ICERs) for rotavirus vaccination in 195 countries.

**Methods:**

Beginning with Tufts University CEA and Global Health CEA registries we used 515 ICERs from 68 articles published through 2017, extracted 938 additional one-way sensitivity analyses, and excluded 33 ICERs for a sample of 1,418. We used a five-stage, mixed-effects, Bayesian metaregression framework to predict ICERs, and logistic regression model to predict the probability that the vaccine was cost-saving. For both models, covariates were vaccine characteristics including efficacy, study methods, and country-specific rotavirus disability-adjusted life-years (DALYs) and gross domestic product (GDP) per capita. All results are reported in 2017 United States dollars.

**Results:**

Vaccine efficacy, vaccine cost, GDP per capita and rotavirus DALYs were important drivers of variability in ICERs. Globally, the median ICER was $2,289 (95% uncertainty interval (UI): $147–$38,993) and ranged from $85 per DALY averted (95% UI: $13–$302) in Central African Republic to $70,599 per DALY averted (95% UI: $11,030–$263,858) in the United States. Among countries eligible for support from Gavi, The Vaccine Alliance, the mean ICER was $255 per DALY averted (95% UI: $39–$918), and among countries eligible for the PAHO revolving fund, the mean ICER was $2,464 per DALY averted (95% UI: $382–$3,118).

**Conclusion:**

Our findings incorporate recent evidence that vaccine efficacy differs across locations, and support expansion of rotavirus vaccination programs, particularly in countries eligible for support from Gavi, The Vaccine Alliance.

## 1 Introduction

Mortality due to diarrheal diseases has declined by 60% since the year 2000, but diarrheal diseases remain one of the leading causes of death for children under 5 every year, and were responsible for an estimated 497,434 childhood deaths in 2019 [1]. Rotavirus is a leading etiology of diarrheal disease mortality globally, responsible for 30% percent of diarrheal deaths among children under 5 [2].

Some of the recent decline in mortality has been attributed to rotavirus vaccination, which averted an estimated 28,000 deaths in 2016 [2]. By the end of 2018, Gavi, The Vaccine Alliance, supported rotavirus vaccination programs in 45 of 57 eligible countries, covering 39% of children in those countries with a full course of a rotavirus vaccine [3]. By April 2020, a total of 107 countries introduced rotavirus vaccines to their routine immunization schedules [4].

As more countries consider introducing rotavirus vaccination into their routine immunization programs, cost effectiveness of the vaccine can inform their decisions. However, cost effectiveness analyses (CEAs) are not available in 53 countries. Among the 142 countries with existing CEA, 49 have estimates that are based on a single study, meaning that the results are sensitive to the modeling assumptions of the study authors and do not consider the cumulative evidence. For countries with more than one CEA, results can vary greatly. For example, the 20 published incremental cost-effectiveness ratios (ICERs) for Peru vary from $132 to $2,438 per DALY averted. Similarly, in Bangladesh, the 19 published ICERs vary from $23 to $1,543 per DALY averted. Because of this variability, efforts to synthesize CEA results and transfer them to other settings are growing. Two systematic reviews synthesized published evidence of CEA on rotavirus vaccines [5], [6]. The first compared methodological approaches between high-income (HIC) and low-income (LIC) countries [5]. The second summarized study characteristics and findings, identifying vaccine efficacy as an important source of variability [6].

More recent cost-effectiveness research relied on meta-analysis to synthesize evidence from studies conducted in multiple countries. Haider et al. used a meta-regression approach to estimate the cost-effectiveness of the rotavirus vaccine across 29 LIC and lower-middle income (LMIC) countries [7]. They produced pooled estimates for the LICs and LMICs under consideration, rather than providing country-specific estimates. Further, their analysis incorporated only three covariates (Gross Domestic Product [GDP], vaccine coverage, and literacy rate) [7]. Jit et al. investigated cost-effectiveness across five European countries using a single model and incorporating country-specific rotavirus burden. They found that results varied across settings due to differences in rotavirus burden and vaccine price, among other factors [8]. Rosettie et al. developed and applied a meta-regression approach to CEA to the human papilloma virus (HPV) vaccine[9]. They exploited one-way sensitivity analyses extracted from published studies and used covariates for study methods, vaccine characteristics (coverage, cost, type, booster, and target group), and country-specific variables for GDP per capita and HPV burden to predict incremental cost effectiveness ratios (ICERs) across 195 countries.

In this paper we apply meta-regression methods developed by Rosettie et al. to estimate the cost-effectiveness of rotavirus vaccination in 195 countries. We identify and quantify sources of heterogeneity in published CEAs from study methods, and vaccine characteristics, well as economic and epidemiologic conditions in each country (i.e. GDP per capita, rotavirus burden). We focus specifically on vaccine efficacy, because it differs by location [1], [10] and has been identified as a driver of heterogeneity [6], but has not yet been used in any meta-analyses of a vaccine-preventable disease.

## 2 Data and methods

### 2.1 Cost-effectiveness data

We used cost-effectiveness data through 2017 from two comprehensive registries of all published CEA maintained by Tufts University’s Center for Evaluation of Risk and Value in Health: (1) CEA registry with cost per quality-adjusted life year (QALY) results [11], and (2) Global Health CEA registry with cost per disability-adjusted life year (DALY) results [12]. As previously reported, they searched PubMed for English-language articles using keywords “QALYs”, “quality-adjusted” [11] and “cost-utility analysis” for the CEA registry, and “disability-adjusted” or “DALY” for the Global Health CEA registry [12]. Abstracts were screened to identify original estimates, and assigned an International Classification of Diseases, 10th Revision (ICD-10) code to each article, [11], [12] which we subsequently mapped to 2017 Global Burden of Disease, Injury and Risk Factor Study (GBD) causes and etiologies. For rotavirus, the CEA registry contains 30 studies measuring the cost per QALY, while the Global Health CEA registry contains 41 studies measuring the cost per DALY (Supplementary Table S1). Combined, these two registries include 519 incremental cost-effectiveness ratios (ICERs) across 142 countries.

This study complies with the Guidelines for Accurate and Transparent Health Estimates Reporting (GATHER) (Supplementary Table S2).

### 2.2 Data standardization, mapping, and extraction

Building on the registry data, we performed several tasks to create covariates and extend the dataset, as described in the Supplementary Sections 3 and 4, and previously reported [9]. Briefly, we extracted data on 5 study methods variables with missing values in the registry data whenever possible: cost discount rate, QALY/DALY discount rate, health outcome measure, perspective, and time horizon. We collapsed perspectives into two categories: healthcare payer and health sector versus all other categories. To account for economic and epidemiological differences across countries, each ICER was mapped to GBD categories for at least one age group, sex, location, and cause, which were used to pull GDP per capita and rotavirus burden measured as DALYs from diarrhea attributable to rotavirus per 100,000 population for children under 5 for the study year [2]. DALYs combine the effects of mortality and morbidity where a death from rotavirus in a child aged 2 to 4 years is measured as 81.81 years of life lost, and an episode of rotavirus is measured as roughly 0.002 years lived with disability, i.e. 5/365 years duration times an average disability weight of 0.12. We extracted data on four vaccine characteristics: vaccine type (monovalent, pentavalent, or both), coverage, cost, and efficacy. All comparisons were relative to a null comparator defined as no intervention (n = 1,245, 92.6% of final sample), standard of care (n = 98, 7.3%) or placebo (n = 2, 0.1%). When registry entries did not correspond to these categories, we extracted data to recalculate them. Finally, we extracted ICERs and covariates from one-way sensitivity analyses, identifying the covariate that differed, and the reference analysis. ICER values, vaccine costs, and GDP per capita were adjusted to 2017 US dollars ($US).

### 2.3 Inclusion and exclusion criteria

**Fig. 1:**
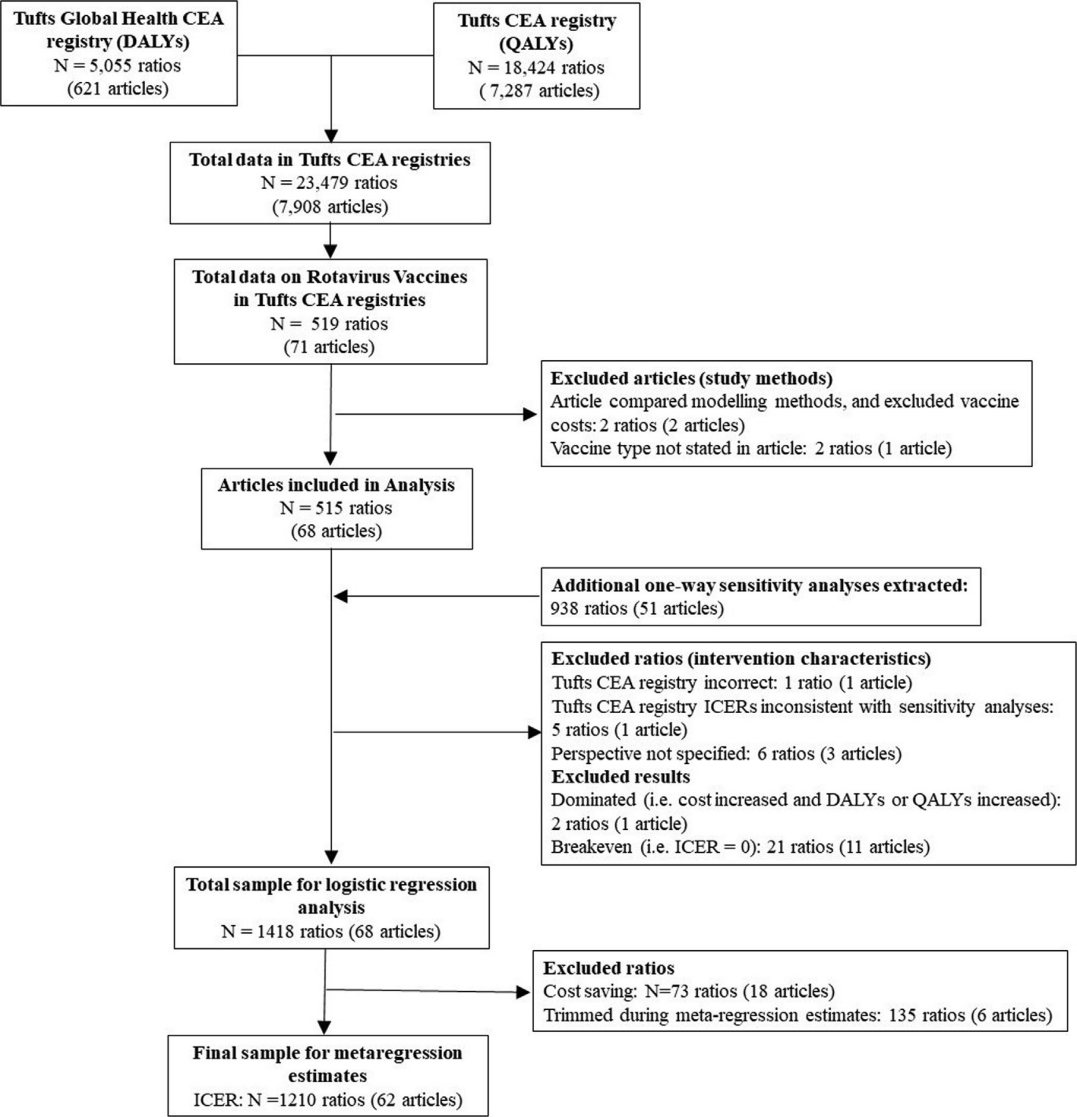
Study flow diagram of study selection in logistic and meta-regression models. * For articles with more than one ratio, some ratios may be excluded and others included. CEA = cost-effectiveness analysis, ICER = incremental cost-effectiveness ratio, QALY = quality-adjusted life-year, DALY = disability-adjusted life-year.

Of the 519 initial cost-effectiveness results from the Tufts database, we excluded two articles that compared modeling methods (two ratios), or did not report vaccine type (two ratios) (Fig. 1). We extracted an additional 938 cost-effectiveness results from one-way sensitivity analyses for seven variables. We also excluded 35 ratios due to missing data, reporting errors, or because the ICER was 0 (Fig. 1).

We conducted a logistic regression analysis (see below) to estimate the probability that a cost-effectiveness result was cost-saving using 1,418 cost-effectiveness results, of which 73 were cost-savings. We conducted the meta-regression analysis with a total of 1,345 ICERs (excluding the cost-savings results) from 142 countries and 68 studies.

### 2.4 Statistical analysis

To estimate ICERs across countries, we adopted a Bayesian mixed-effects meta-regression framework that employs regularization and outlier detection to select covariates and estimate their associations with the observed ICER. We used those estimated associations to predictions ICERs for 195 countries. This framework consists of five stages described in Supplementary Section 5 and previously reported [9], [13].

The first stage estimated prior distributions for seven pre-selected covariates in which one-way sensitivity analyses were done in the published literature: cost discount rate, DALY/QALY discount rate, perspective, vaccine type, vaccine coverage, vaccine cost, and vaccine efficacy. The aim of the sensitivity analyses was to reduce omitted variable bias and identify prior distributions for variables that were highly correlated, such as log-vaccine cost and log-GDP per capita. The one-way sensitivity analyses differed by one covariate and no unmeasured covariates from their corresponding reference analysis. We therefore matched each sensitivity analysis with its corresponding reference analysis and fit separate linear models to estimate the effect of the difference between covariate values in reference and sensitivity analyses on the difference in the corresponding log-ICERs. The estimated regression coefficients and standard errors from these models were then used as Gaussian prior distributions for these covariates in all subsequent models.

The second stage estimated the association between log-ICER and log-GDP per capita, using a spline ensemble that can estimate a non-linear response curve. The estimates included log-rotavirus burden as a covariate. This stage had a robust statistical approach to outlier detection, with 10% of observed log-ICERs being classified by the model as outliers and trimmed from the dataset ahead of further modeling steps. The resulting spline-transformed GDP per capita variable was used in subsequent stages.

In the third stage, we used a generalized Lasso approach to select additional covariates to include in the final model, thereby balancing covariate selection from a priori knowledge versus statistical approaches. The seven covariates from the first stage, and the spline-transformed log-GDP per capita variables were preselected. Candidate covariates for the model were: whether or not the study used a lifetime time horizon, whether the outcome measure was QALYs or DALYs, and log-rotavirus burden, all of which were selected for the final meta-regression model.

In the fourth stage, we created Gaussian priors for the spline transformed GDP per capita variable, and the two variables selected in stage 3 to safeguard against overfitting in the final estimates. Given that we had coefficient estimates for these variables from Stage 3, we standardized the variables, and used a grid-search and 10-fold cross validation to select the standard deviation for Gaussian priors. The selected prior standard deviation for the standardized covariates minimized the mean squared error for predicting data in the hold-out set.

In the fifth stage, we fit a linear mixed model that accounted for between-study variability and the fact that studies often reported multiple ICERs as part of sensitivity analyses. The model has a random intercept for each article, and covariates selected in the third stage of the analysis using priors estimated from the first and fourth stages, respectively. The parameter estimates for the covariates were used to predict ICERs for 195 countries.

Using this modeling framework, we explore three different parameterizations of efficacy to determine whether or not its inclusion improves model fit. First, we include efficacy as a main effect. Second, we define effective coverage as the product of coverage and efficacy (scaled to be between 0 and 100%), where covered population is the primary beneficiary of the intervention. Finally, we estimate a model without efficacy. All model comparisons are based on R-squared and root mean square error (RMSE) fit statistics, with the best fitting model used for final results. The model with efficacy as the main effect had the best fit (R-square = 0.962, RMSE = 0.607), and was used for final results.

We estimated the probability that rotavirus vaccination was cost-saving using a mixed effects logistic regression as described in Supplementary Section 6. The model was fit subsequent to the meta-regression and included the same final set of covariates, but used the full data set with 1,418 results instead of the subset of the data selected in the trimming stage of the analysis. As with the meta-regression, we estimate a random intercept for each article.

We use the estimates from the meta-regression and logistic regression to predict both the ICER and probability the vaccine was cost-savings for 195 countries as a function of country-level covariates. The predicted ICER with adjustment is the product of the predicted ICER and (one minus the cost-saving probability). The predicted cost-saving probabilities were small, as were the adjustments to the predicted ICERs.

Our predictions assume 90% vaccine coverage (the GAVI target), lifetime time horizon, healthcare payer perspective, 3% burden and cost discount rates, monovalent vaccine type, and DALYs as the outcome measure. Our estimate of country-specific vaccine efficacy comes from GBD [1]. Because the rotavirus vaccine does not have a single market price, we used the cost of all required doses based on the 2017 cost per dose reported in the WHO’s Market Information for Access (MIA4) and aggregated by Linksbridge [14], [15] (see Supplementary Section 7). We used the listed UNICEF price for the 57 Gavi-eligible countries [16], and Pan American Health Organization (PAHO) for 22 countries eligible for PAHO Revolving Fund.

We predict both the mean ICER and 95% UI, which are then adjusted by the probability that the intervention is cost-saving. We report two ratios: 1) the adjusted mean predicted ICER to GDP per capita, and the upper bound of the UI of the adjusted predicted ICER to GDP per capita. While GDP per capita has been criticized as a cost-effectiveness threshold as outdated [17], [18], and not an accurate measure of a population’s preference for spending on health improvements [19], the ratios may provide a useful screening tool [19] and place the results in the context of the country’s economy.

## 3 Results

The sub-Saharan Africa region reported more results than any other region (4 4 0), followed by high-income countries (3 3 9), Southeast Asia, East Asia and Oceana (1 7 7), Latin America and Caribbean (1 3 7), Central Europe, Eastern Europe, and Central Asia (1 0 7), South Asia (83), and North Africa and the Middle East (62). (Table 1). A total of 521 ICERs were reported from the healthcare payer perspective, followed by 478 from the limited societal, 329 societal, and 17 from the healthcare sector perspectives. Most ICERs (1,029) measured health outcomes with DALYs, and used a 3% discount rate (1,058). Median vaccine efficacy across studies was 82% (Interquartile Range [IQR] 64-87). Median vaccine coverage was 75% (IQR 70-90), and median vaccine cost was $7.41 (IQR $3.61 - $59.30) which included all doses.

**Table 1:** Descriptive statistics of cost-effectiveness results and peer-reviewed articles on rotavirus vaccines included the analysis.

	**Number of ratios reported in Tufts registries (%)**	**Number of ratios reported in Tufts registries plus sensitivity analyses extracted (%)**	Number of articles reported in Tufts registries plus sensitivity analyses* (%)
**Sample size**	483	1,345	68

**Study characteristics**			
Region			
Sub-Saharan Africa	176 (36.4)	440 (32.7)	14 (20.6)
High-income	51 (10.6)	339 (25.2)	29 (42.6)
Southeast Asia, East Asia & Oceania	69 (14.3)	177 (13.2)	12 (17.6)
Latin America & Caribbean	57 (11.8)	137 (10.2)	8 (11.8)
Central Europe, Eastern Europe, & Central Asia	51 (10.6)	107 (8.0)	5 (7.4)
North Africa and the Middle East	35 (7.2)	62 (4.6)	10 (14.7)
South Asia	44 (9.1)	83 (6.2)	11 (16.2)
Year published			
2005	1 (0.2)	6 (0.4)	1 (1.5)
2006	0 (0.0)	0 (0.0)	0 (0.0)
2007	12 (2.5)	59 (4.4)	3 (4.4)
2008	8 (1.7)	43 (3.2)	5 (7.4)
2009	14 (2.9)	226 (16.8)	10 (14.7)
2010	78 (16.1)	434 (32.3)	5 (7.4)
2011	81 (16.8)	183 (13.6)	8 (11.8)
2012	17 (3.5)	54 (4.0)	7 (10.3)
2013	7 (1.4)	27 (2.0)	5 (7.4)
2014	15 (3.1)	27 (2.0)	6 (8.8)
2015	232 (48.0)	253 (18.8)	10 (14.7)
2016	2 (0.4)	14 (1.0)	2 (2.9)
2017	16 (3.3)	19 (1.4)	6 (8.8)

**Study methods**			
Cost discount rate			
< 3%	5 (1.0)	95 (7.1)	10 (14.7)
3%	457 (94.6)	1,065 (79.2)	50 (73.5)
> 3%	21 (4.3)	185 (13.8)	15 (22.1)
Health outcome measure			
QALYs	42 (8.7)	316 (23.5)	27 (39.7)
DALYs	441 (91.3)	1,029 (76.5)	41 (60.3)
QALY/DALY discount rate			
<3%	12 (2.5)	181 (13.5)	19 (27.9)
3%	453 (93.8)	1,052 (78.2)	52 (76.5)
>3%	18 (3.7)	112 (8.3)	17 (25.0)
Perspective			
Societal	105 (21.7)	329 (24.5)	38 (55.9)
Limited societal	94 (19.5)	478 (35.5)	11 (16.2)
Healthcare payer	272 (56.3)	521 (38.7)	50 (73.5)
Health sector	12 (2.5)	17 (1.3)	3 (4.4)
Time Horizon			
Lifetime	241 (49.9)	307 (22.8)	15 (22.1)
Less than lifetime	242 (50.1)	1,038 (77.2)	53 (77.9)

**Vaccine characteristics**			
Type of vaccine			
Monovalent	180 (37.3)	488 (36.3)	48 (70.6)
Pentavalent	152 (31.5)	318 (23.6)	34 (52.9)
Monovalent & Pentavalent	151 (31.3)	579 (43.0)	6 (8.8)
	**Median (IQR)**	**Median (IQR)**	
Vaccine coverage	83 (70,94)	75 (70,90)	68
Vaccine cost (2017 USD)	$6.41 (4.85, 9.99)	$7.41 (3.61, 59.30)	68
Vaccine efficacy	83.7 (63, 86)	82 (64, 87)	68
Vaccine effective coverage	59.5 (53.4, 78.3)	59.5 (53.8, 82.5)	68

An asterisk (*) denotes that the total number of articles may exceed 68 since some articles examined multiple regions, vaccine characteristics, and cost-effectiveness analyses characteristics. QALY = quality-adjusted life-year, DALY = disability-adjusted life-year, IQR = interquartile range.

In meta-regression analysis, vaccine efficacy and log-DALYs per capita were negatively associated with the log-ICER, and log-vaccine cost and log-GDP per capita were positively associated with it (Supplementary Table S5). A 10 % increase in burden would reduce the ICER by 4.2%, and a 10% increase in vaccine price would increase the ICER by 6.4%. Including vaccine efficacy in the analysis slightly improved model fit; R^2^ increased from 0.960 to 0.961, while Root mean-squared error fell from 0.613 to 0.607. The random effects variance for the model including efficacy was also lower (0.576 including efficacy versus 0.593). We did not observe similar improvements for models of effective coverage (Supplementary Table S6). We report estimates for the model including efficacy in the main text, and estimates for the model without efficacy in Supplementary Table S9.

**Fig. 2:**
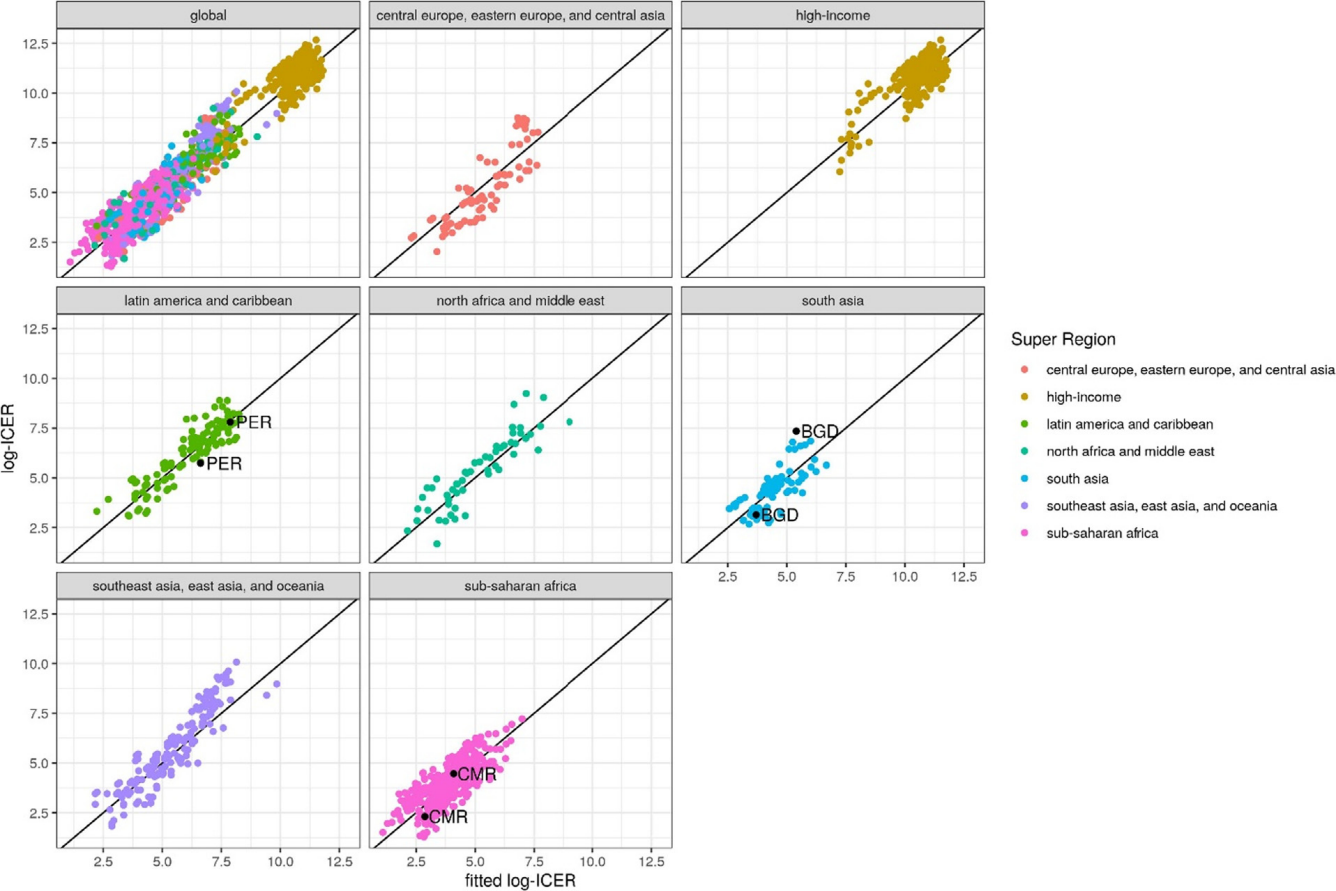
Observed log-ICER vs fitted log-ICER overall and by GBD super-region. Fig. 2**legend.** Observed log-ICER vs fitted log-ICER overall and by GBD super region. The minimum and maximum values for Peru (PER), Bangladesh (BGD), and Cameroon (CMR) are shown to highlight differences in observed log-ICERs across studies within a given country. ICER = incremental cost-effectiveness ratio.

The ranges of observed ICERs within countries were broad due to differences in methods and intervention characteristics. The ranges of observed ICERs within super-regions were broad for the same reasons as well as differences in rotavirus burden and GDP among countries. Despite these differences, the final meta-regression model fit the data well, as shown in the comparison of the observed and fitted ICERs globally and by super-region (Fig. 2).

Among the seven GBD super-regions (Table 2), Sub-Saharan Africa and South Asia had the lowest population-weighted mean ICERs among GBD super-regions. Among the 46 countries making up the sub-Saharan African region, the mean predicted ICER was $251 per DALY averted (90% UI 38–903), while for the 5 countries making up the South Asia region, the mean predicted ICER was $294 per DALY averted (95% UI 45–1 062). The 34 countries making up the high-income region had the highest ICER ($40 914 per DALY averted; 95% UI 6 382–153 130).

**Table 2:** Predicted incremental cost-effectiveness ratios aggregated to super-region level compared to range of input data by super-region from Tufts registries.

**Super Region**	**Predicted ICER adjusted for cost-saving probabilities in 2017 US$ per DALY Averted (95% UI)**	**Tufts registry dataset and additional extractions**			
		**Minimum ICER in Tufts data and sensitivity analyses extractions in 2017 US$ per unit change in DALY or QALY**	**Minimum ICER location in Tufts data**	**Maximum ICER in Tufts data and sensitivity analyses extractions in 2017 US$ per unit change in DALY or QALY**	**Maximum ICER location in Tufts data**
High-Income	40,915 (6,382 to 153,130)	95	Argentina	319,574	Spain
North Africa and Middle East	2,407 (374 to 8,884)	3	Iran	10,224	Iran
Central Europe, Eastern Europe, and Central Asia	3,993 (618 to 14,726)	1	Uzbekistan	12,964	Albania
Southeast Asia, East Asia, and Oceania	2,408 (375 to 8,845)	1	Cambodia	418,802	Mauritius
Latin America and Caribbean	2,454 (380 to 9,085)	2	Haiti	17,604	Cuba
South Asia	294 (45 to 1,065)	15	Pakistan	1,543	Bangladesh
Sub-Saharan Africa	251 (38 to 903)	0	Angola	1,373	Cape Verde

Population-weighted super-region predictions assuming 90% vaccine coverage, lifetime time horizon, healthcare payer perspective, 3% vaccine cost and burden discount rates, monovalent vaccine type, DALYs averted as health outcome measure, and no intervention as the comparator. ICER = incremental cost-effectiveness ratio, UI = uncertainty interval, QALY = quality-adjusted life-year, DALY = disability adjusted life-year.

**Fig. 3:**
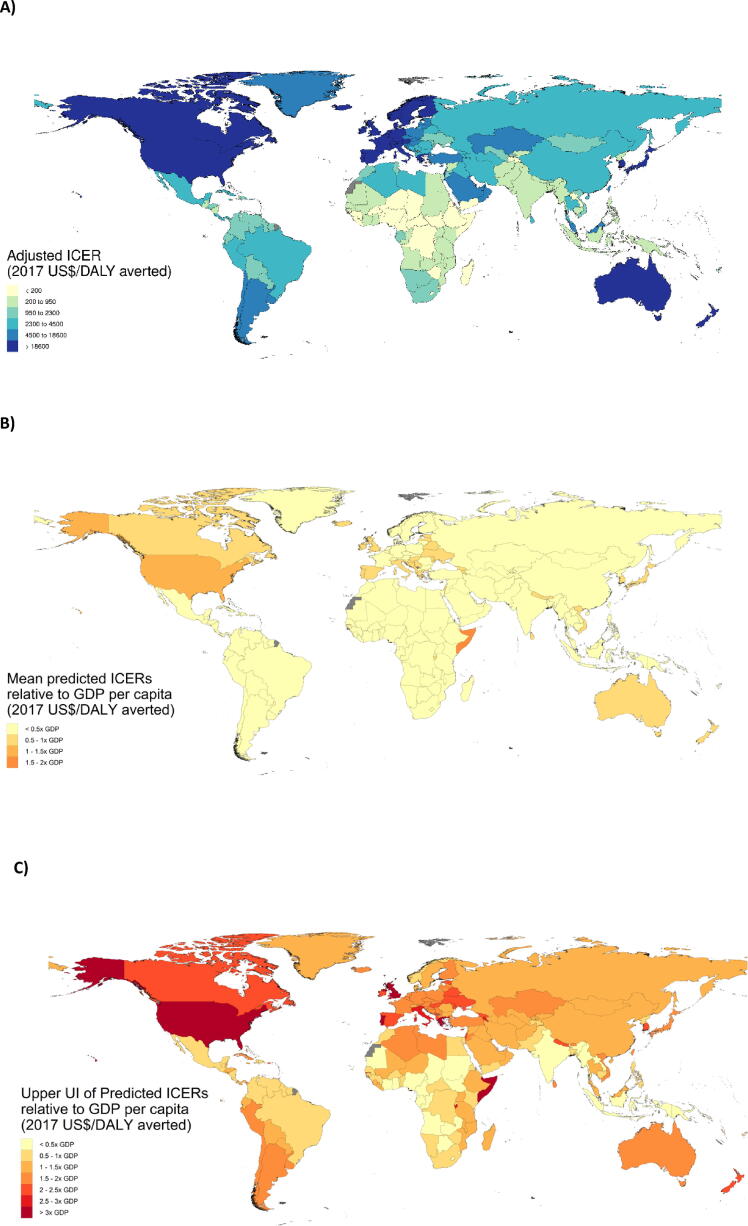
**Predicted ICERs from meta-regression analysis by country.**Fig. 3**legend.** (A) Predicted ICERs from meta-regression analysis by country in 2017 USD per DALY averted; (B) Predicted ICERs relative to seven categories of GDP per capita ranging from <0.5 to >3.0 times GDP; C) GDP category in which the upper bound of the 95% UI falls. ICER = incremental cost-effectiveness ratio, UI = uncertainty interval, DALY = disability-adjusted life-year.

Predicted ICERs varied across countries (Table 3; Fig. 3A). Among all 195 countries, the lowest mean adjusted ICERs were observed in Central African Republic (2017 US$ 85 per DALY averted; 95% UI 13–302), and Chad ($120 per DALY averted; 95% UI 18–428), which have the two highest burdens worldwide. The highest mean predicted ICERs were observed in the US ($70,599 per DALY averted; 95% UI 11,030–263,858), and Luxembourg ($46,158 per DALY averted; 95% UI 7,256–169,808), which are in the bottom 8% of rotavirus burden worldwide. Predicted ICERs may be outside the range of observed ICERs, due to differences in parameters in the published and predicted results, such as parameters such as vaccine cost and efficacy.

**Table 3:** Predicted incremental cost-effectiveness ratios by country adjusted for cost-saving probabilities.

**Country**	**Predicted ICER adjusted for cost-saving probabilities in 2017 US$ per DALY Averted (95% UI)**	**Rotavirus Vaccine Efficacy % (95% UI)**	**Rotavirus diarrhea DALYs per 100,000 children 0 to 5 years of age**	**Tufts registry dataset and additional extractions**		
				**Number of ratios**	**Minimum ICER in 2017 US$ per DALY or QALY**	**Maximum ICER in 2017 US$ per DALY or QALY**
Central Europe Eastern Europe and Central Asia						
Albania	3,373 (523–12,365)	78 (64–92)	84.0	4	446	12,964
Armenia	3,985 (616–14,618)	79 (65–92)	52.1	16	20	5,630
Azerbaijan	2,678 (418–9,830)	78 (65–92)	249.5	9	9	100
Belarus	4,138 (646–15,202)	84 (73–95)	62.6	2	2,989	3,626
Bosnia and Herzegovina	3,166 (493–11,609)	81 (68–93)	114.7	2	6,160	6,949
Bulgaria	3,364 (526–12,393)	84 (73–95)	170.7	2	4,584	6,370
Croatia	8,713 (1,354–32,168)	85 (75–96)	105.8	0	NA	NA
Czech Republic	11,215 (1,732–41,604)	87 (78–96)	110.0	0	NA	NA
Estonia	13,140 (2,030–48,729)	87 (79–96)	55.6	0	NA	NA
Georgia	2,977 (461–10,944)	80 (67–93)	52.0	10	61	4,274
Hungary	10,063 (1,558–37,231)	85 (75–96)	89.4	0	NA	NA
Kazakhstan	5,927 (924–21,960)	81 (69–94)	87.6	2	585	689
Kyrgyzstan	499 (75–1,805)	73 (56–89)	228.1	10	22	685
Latvia	9,248 (1,435–34,147)	87 (78–96)	93.9	0	NA	NA
Lithuania	8,295 (1,277–30,606)	88 (79–97)	134.8	0	NA	NA
Macedonia	2,535 (395–9,279)	83 (71–94)	167.0	2	5,866	6,849
Moldova	1,430 (220–5,199)	80 (67–93)	117.3	10	839	5,272
Mongolia	1,484 (230–5,436)	72 (55–88)	368.6	10	27	370
Montenegro	4,546 (710–16,772)	85 (74–95)	64.4	0	NA	NA
Poland	7,712 (1,193–28,438)	86 (76–96)	142.1	0	NA	NA
Romania	3,406 (531–12,535)	83 (72–95)	248.0	2	2,163	4,570
Russian Federation	4,410 (682–16,277)	86 (76–96)	177.6	0	NA	NA
Serbia	3,531 (552–12,977)	84 (74–95)	92.5	0	NA	NA
Slovakia	9,176 (1,415–33,959)	86 (77–96)	145.7	0	NA	NA
Slovenia	12,266 (1,898–45,563)	88 (79–97)	105.7	0	NA	NA
Tajikistan	175 (26–632)	66 (48–84)	3,114.2	10	2	185
Turkmenistan	3,508 (547–12,919)	77 (63–92)	173.1	0	NA	NA
Ukraine	2,058 (316–7,512)	83 (71–94)	66.9	10	1,641	4,783
Uzbekistan	1,131 (173–4,120)	75 (59–90)	58.5	6	1	42
High-Income						
Andorra	25,784 (4,010–96,684)	90 (83–98)	46.2	0	NA	NA
Argentina	6,742 (1,050–24,866)	80 (68–93)	178.9	14	95	21,814
Australia	40,076 (6,255–149,868)	88 (79–96)	36.5	11	2,765	68,147
Austria	26,603 (4,141–99,919)	88 (80–97)	52.5	0	NA	NA
Belgium	24,312 (3,783–91,206)	88 (80–97)	58.4	61	5,630	119,778
Brunei	21,638 (3,358–81,067)	86 (76–96)	59.6	0	NA	NA
Canada	36,697 (5,723–138,239)	89 (81–97)	27.9	14	2,078	117,187
Chile	8,156 (1,256–30,162)	83 (72–95)	168.2	7	2,237	35,200
Cyprus	16,771 (2,593–62,496)	88 (79–96)	56.0	0	NA	NA
Denmark	19,141 (2,972–71,359)	90 (82–97)	141.4	0	NA	NA
Finland	23,096 (3,594–86,609)	88 (80–97)	68.2	30	4,820	151,315
France	22,273 (3,459–83,480)	88 (79–96)	65.4	46	18,843	249,924
Germany	22,090 (3,439–82,722)	90 (83–98)	67.7	8	80,059	209,020
Greece	22,004 (3,401–82,156)	86 (75–96)	23.9	0	NA	NA
Greenland	18,586 (2,881–69,232)	88 (79–96)	121.7	0	NA	NA
Iceland	27,239 (4,246–102,293)	89 (81–97)	47.2	0	NA	NA
Ireland	47,446 (7,397–177,120)	89 (81–97)	26.7	5	59,838	206,479
Israel	22,304 (3,463–83,589)	86 (76–96)	54.8	12	3,284	96,391
Italy	28,493 (4,433–106,974)	86 (76–96)	27.8	0	NA	NA
Japan	30,082 (4,686–113,113)	89 (81–97)	42.1	4	7,527	85,246
Luxembourg	46,158 (7,256–169,808)	90 (82–97)	50.3	0	NA	NA
Malta	22,324 (3,458–83,507)	85 (75–96)	32.9	0	NA	NA
Netherlands	27,239 (4,243–102,313)	90 (82–97)	53.1	57	3,770	168,172
New Zealand	26,123 (4,066–98,009)	88 (79–97)	41.0	2	39,221	57,018
Norway	26,145 (4,093–96,292)	91 (84–98)	128.0	2	51,044	56,705
Portugal	21,739 (3,366–81,290)	82 (71–94)	27.6	0	NA	NA
Singapore	29,503 (4,601–110,505)	89 (81–97)	55.9	0	NA	NA
South Korea	18,869 (2,914–70,329)	89 (82–97)	40.5	8	107	354
Spain	22,953 (3,566–86,037)	84 (73–95)	42.2	10	26,761	319,574
Sweden	25,135 (3,921–93,656)	90 (82–97)	80.6	0	NA	NA
Switzerland	28,464 (4,461–105,356)	92 (85–98)	91.5	0	NA	NA
United Kingdom	45,242 (7,052–170,512)	89 (81–97)	11.7	44	39,730	189,763
Uruguay	7,965 (1,226–29,489)	79 (66–93)	182.6	2	1,470	1,525
United States	70,599 (11,030–263,858)	89 (80–97)	45.5	2	121,302	223,843
Latin America and Caribbean						
Antigua and Barbuda	8,119 (1,261–29,994)	82 (70–94)	130.7	0	NA	NA
Barbados	10,691 (1,655–39,635)	82 (70–94)	86.5	0	NA	NA
Belize	1,465 (227–5,379)	72 (56–89)	310.5	2	544	621
Bermuda	38,801 (6,085–144,020)	87 (77–96)	54.6	0	NA	NA
Bolivia	960 (147–3,508)	69 (51–86)	562.1	10	25	388
Brazil	2,848 (439–10,582)	75 (59–90)	277.7	9	856	7,223
Colombia	2,279 (354–8,427)	74 (58–90)	245.8	4	892	2,461
Costa Rica	2,892 (450–10,714)	78 (64–92)	183.8	2	5,186	5,313
Cuba	3,183 (495–11,752)	77 (62–91)	72.9	8	6,674	17,604
Dominica	1,7008 (265–6,246)	82 (70–94)	276.8	0	NA	NA
Dominican Republic	1,930 (300–7,129)	71 (54–88)	367.4	7	494	1,036
Ecuador	2,084 (323–7,672)	75 (59–90)	163.1	0	NA	NA
El Salvador	1,332 (205–4,879)	69 (51–86)	377.8	0	NA	NA
Grenada	3,361 (523–12,448)	77 (62–92)	99.4	0	NA	NA
Guatemala	603 (93–2,191)	63 (44–82)	2,533.1	2	662	906
Guyana	982 (152–3,590)	73 (57–89)	741.3	10	3	2,973
Haiti	224 (345–807)	55 (34–75)	2,110.5	10	2	174
Honduras	980 (149–3,562)	62 (42–81)	598.4	15	51	1,089
Jamaica	2,223 (345–8,170)	78 (64–92)	110.3	2	925	925
Mexico	2,742 (426–10,168)	76 (61–91)	230.1	10	589	3,232
Nicaragua	586 (89–2,108)	64 (45–83)	330.7	10	100	1,106
Panama	4,317 (665–15,877)	78 (64–92)	531.2	7	70	3,295
Paraguay	1,975 (305–7,259)	75 (60–90)	144.2	2	1,477	1,615
Peru	3,464 (538–12,765)	75 (60–91)	62.0	20	132	2,438
Puerto Rico	16,041 (2,482–59,786)	87 (77–96)	64.8	0	NA	NA
Saint Lucia	2,774 (432–10,261)	77 (62–92)	144.9	2	2,567	2,568
Saint Vincent and the Grenadines	2,006 (312–7,382)	73 (57–89)	247.2	0	NA	NA
Suriname	1,441 (224–5,299)	75 (59–90)	536.4	0	NA	NA
The Bahamas	13,998 (2,167–52,117)	86 (76–96)	89.4	0	NA	NA
Trinidad and Tobago	8,415 (1,296–31,129)	84 (73–95)	165.3	0	NA	NA
Venezuela	1,485 (231–5,467)	74 (58–90)	575.2	5	622	1,540
Virgin Islands	18,944 (2,936–70,770)	87 (77–96)	54.1	0	NA	NA
North Africa and Middle East						
Afghanistan	221 (33–790)	42 (21–64)	3,324.8	13	5	82
Algeria	2,579 (401–9,461)	76 (61–91)	218.5	2	828	1,070
Bahrain	14,100 (2,180–52,529)	84 (72–95)	82.7	0	NA	NA
Egypt	553 (85–2,002)	74 (59–90)	1,646.9	3	424	553
Iran	2,916 (455–10,725)	78 (64–92)	300.9	10	3	10,224
Iraq	2,488 (387–9,129)	75 (60–90)	301.2	2	219	270
Jordan	2,069 (320–7,536)	80 (67–93)	196.8	2	1,842	1,873
Kuwait	14,899 (2,319–55,585)	88 (79–96)	130.2	0	NA	NA
Lebanon	3,109 (485–11,432)	79 (65–93)	225.1	0	NA	NA
Libya	2,907 (454–10,668)	81 (69–94)	146.2	1	8,411	8,411
Morocco	1,019 (157–3,717)	66 (48–84)	711.8	2	672	871
Oman	7,255 (1,120–26,810)	84 (73–95)	244.8	1	2,462	2,462
Palestine	1,772 (273–6,426)	67 (49–85)	137.0	0	NA	NA
Qatar	33,499 (5,241–124,349)	88 (79–96)	61.1	0	NA	NA
Saudi Arabia	9,400 (1,455–34,840)	86 (76–96)	184.2	0	NA	NA
Sudan	303 (46–1,101)	62 (43–82)	2,206.7	10	62	316
Syria	694 (105–2,508)	73 (57–89)	118.3	2	720	753
Tunisia	2,178 (337–8,000)	77 (63–92)	117.2	2	1,387	1,412
Turkey	6,660 (1,034–24,757)	79 (66–93)	92.8	2	596	1,319
United Arab Emirates	17,361 (2,704–64,889)	89 (81–97)	112.6	0	NA	NA
Yemen	158 (24–568)	56 (36–76)	4,085.9	10	18	408
South Asia						
Bangladesh	343 (52–1,235)	59 (40–79)	829.8	19	23	1,543
Bhutan	1,780 (273–6,467)	57 (37–77)	236.1	10	15	280
India	260 (40–945)	67 (49–85)	1,981.5	29	17	294
Nepal	530 (80–1,910)	53 (32–74)	298.7	10	23	776
Pakistan	392 (59–1,408)	56 (35–76)	791.4	15	15	371
Southeast Asia East Asia and Oceania						
American Samoa	3,435 (536–12,634)	82 (69–94)	195.0	0	NA	NA
Cambodia	345 (52–1,240)	59 (39–79)	869.3	9	1	99
China	3,301 (515–12,134)	79 (66–93)	173.9	35	688	213,789
Federated States of Micronesia	1,568 (240–5,722)	70 (53–87)	192.0	2	960	1,057
Fiji	1,484 (231–5,430)	77 (62–91)	692.1	2	1,971	2,179
Guam	10,513 (1,638–39,079)	87 (77–96)	236.8	0	NA	NA
Indonesia	629 (98–2,287)	75 (60–91)	2,402.1	13	30	468
Kiribati	672 (102–2,447)	65 (46–83)	1,121.5	8	4	153
Laos	182 (28–657)	61 (41–80)	5,772.2	10	6	111
Malaysia	4,885 (762–18,060)	82 (69–94)	127.7	2	2,488	2,899
Maldives	4,641 (721–17,186)	73 (57–89)	127.7	2	19,039	23,812
Marshall Islands	2,289 (352–8,393)	67 (49–85)	225.3	0	NA	NA
Mauritius	3,493 (544–12,879)	80 (67–93)	264.7	1	418,802	418,802
Myanmar	251 (38–907)	63 (44–82)	2,107.3	10	7	238
North Korea	306 (46–1,103)	68 (50–86)	741.2	0	NA	NA
Northern Mariana Islands	8,104 (1,250–29,962)	85 (75–96)	183.9	0	NA	NA
Papua New Guinea	331 (50–1,196)	54 (33–74)	2,501.9	8	20	181
Philippines	719 (111–2,615)	72 (56–89)	1,221.6	2	404	446
Samoa	2,346 (363–8,580)	75 (60–91)	196.7	2	1,527	1,698
Seychelles	7,870 (1,223–29,077)	81 (69–94)	140.7	0	NA	NA
Solomon Islands	480 (73–1,729)	53 (32–73)	662.8	10	168	990
Sri Lanka	2,419 (373–8,872)	79 (66–93)	70.7	10	190	4,144
Taiwan (Province Of China)	11,185 (1,728–41,464)	89 (81–97)	109.3	5	308	7,903
Thailand	2,483 (387–9,102)	77 (63–92)	293.7	3	126	4,335
Timor-Leste	722 (111–2,620)	64 (46–83)	1,617.6	10	19	302
Tonga	2,577 (400–9,441)	75 (60–90)	187.2	2	2,869	3,170
Vanuatu	1,047 (160–3,802)	61 (41–80)	768.7	2	2,878	3,254
Vietnam	1,227 (189–4,464)	73 (57 89)	205.3	29	82	3,548
Sub-Saharan Africa						
Angola	566 (88–2,057)	58 (38–78)	5,893.8	8	0	107
Benin	165 (25–589)	45 (24–66)	6,770.6	10	11	134
Botswana	1,217 (189–4,452)	74 (59–90)	2,682.2	2	298	459
Burkina Faso	148 (22–525)	37 (17–57)	10,865.4	10	9	99
Burundi	187 (28–667)	40 (19–60)	3,689.4	10	11	103
Cameroon	180 (27–650)	58 (39–78)	4,953.5	10	10	87
Cape Verde	1,298 (199–4,723)	63 (44–82)	492.2	2	1,041	1,373
Central African Republic	85 (13–302)	40 (20–61)	27,445.5	10	14	117
Chad	120 (18–428)	33 (14–52)	23,156.9	9	7	47
Comoros	206 (31–744)	59 (40–79)	2,673.6	10	71	210
Congo (Brazzaville)	209 (32–757)	68 (50–86)	4,290.1	11	17	149
Cote D'Ivoire	201 (31–722)	51 (30–72)	5,289.2	10	10	184
Djibouti	310 (47–1,118)	54 (34–75)	1,768.6	7	17	121
DR Congo	124 (19–443)	47 (26–68)	9,297.0	10	15	106
Equatorial Guinea	1,714 (267–6,306)	76 (61–91)	2,480.6	0	NA	NA
Eritrea	168 (263–602)	52 (31–73)	4,185.5	10	72	635
Ethiopia	192 (29–686)	44 (23–66)	3,995.0	11	13	145
Gabon	1,438 (223–5,279)	74 (58–90)	2,426.4	0	NA	NA
Ghana	207 (32–750)	67 (50–85)	3147.3	17	2	519
Guinea	182 (28–659)	42 (21–63)	5116.5	10	8	80
Guinea-Bissau	142 (225–507)	46 (24–67)	8,414.6	10	4	70
Kenya	207 (31–749)	62 (42–81)	2,547.8	25	22	553
Lesotho	184 (28–665)	62 (43–82)	4,207.3	10	184	790
Liberia	167 (25–595)	46 (25–68)	4,966.2	10	10	144
Madagascar	164 (25–586)	48 (27–69)	4,728.8	10	7	206
Malawi	221 (33–796)	48 (27–69)	2,354.5	25	2	194
Mali	296 (45–1,056)	37 (17–57)	2,326.7	10	2	60
Mauritania	215 (33–776)	60 (41–80)	3,046.7	10	8	75
Mozambique	254 (38–911)	41 (20–62)	2,327.8	10	13	98
Namibia	1,265 (197–4,623)	72 (55–88)	1,643.8	2	650	819
Niger	166 (25–588)	24 (8–41)	10,655.5	10	5	63
Nigeria	133 (20–479)	62 (43–81)	14,002.9	11	1	74
Rwanda	185 (28–664)	54 (33–74)	3,653.4	10	0	127
Sao Tome and Principe	349 (52–1,238)	60 (40–80)	902.0	10	27	385
Senegal	210 (32–753)	51 (30–71)	3,732.1	24	15	174
Sierra Leone	167 (25–593)	44 (23–65)	5,362.5	10	1	71
Somalia	168 (25–592)	21 (6–36)	7,146.1	10	4	30
South Africa	1,575 (246–5,758)	78 (64–92)	1,114.7	2	124	186
South Sudan	151 (23–540)	40 (19–61)	14,259.4	0	NA	NA
Swaziland	467 (73–1,699)	69 (52–87)	5,669.8	2	222	302
Tanzania	370 (56–1,331)	52 (31–73)	790.3	11	16	160
The Gambia	332 (50–1,199)	50 (30–71)	836.8	10	20	160
Togo	153 (23–548)	51 (30–72)	5,594.9	10	20	216
Uganda	218 (33–783)	50 (29–71)	2,580.0	13	11	80
Zambia	234 (36–844)	60 (41–80)	2,967.4	10	10	118
Zimbabwe	197 (30–709)	59 (39–79)	2,773.1	8	18	246

Country predictions assuming 90% vaccine coverage, lifetime time horizon, payer perspective, 3% cost and burden discount rates, monovalent vaccine type, DALYs averted as health outcome measure, vaccine cost (for 2-dose course) and no intervention as the comparator. ICER = incremental cost-effectiveness ratio, UI = uncertainty interval, QALY = quality-adjusted life-year, DALY = disability adjusted life-year.

Among countries eligible for support from Gavi, The Vaccine Alliance, the mean ICER was $255 per DALY averted (95% UI: $39 - $918). Among countries eligible for the PAHO revolving fund, the mean ICER was $2,464 per DALY averted (95% UI: $382 - $3,118).

Considering these results in the context of each country’s economy, 21 countries had adjusted ICERs with upper UIs less than one-half times GDP per capita (Fig. 3c). Among them, 15 were in the sub-Saharan Africa region, 4 in Southeast Asia, East Asia, and Oceana, one in South Asia (India), and one in North Africa and the Middle East (Sudan). An additional 43 countries had upper UIs that were between 0.5 and one times the GDP per capita, including 17 in sub-Saharan Africa, 13 in Latin America and the Caribbean, six in Southeast Asia, East Asia, and Oceana, three in North Africa and the Middle East, two in South Asia, and one each in high-income (Norway) and Central Europe, Eastern Europe, and Central Asia (Tajikistan).

## 4 Discussion

To our knowledge, this is the most comprehensive meta-regression analysis of CEA of rotavirus vaccination conducted, building on previous work that focused on 29 LICs and LIMCs [7], and previous efforts to leverage a meta-regression approach to synthesize all available evidence on HPV and provide cost-effectiveness estimates for 195 countries worldwide [9].

Unlike previous approaches, our approach facilitates the transferability of published estimates across settings, while accounting for differences between settings. Currently, a Ministry of Health in a country where there are no cost effectiveness estimates available may look to estimates from neighboring countries. However, these estimates may vary considerably within a country, and there would be no quantitative way to adjust them to account for differences in economic activity, burden of disease, vaccine efficacy or cost. For example, there are no published cost-effectiveness estimates in Equatorial Guinea, while neighboring Cameroon has estimates that range from $10 to $87 per DALY averted (Fig. 2). Relying on estimates from Cameroon would likely underestimate the cost-effectiveness of the intervention because of differences in GDP per capita ($15,803 in Equatorial Guinea vs $1,671 in Cameroon) and corresponding vaccine costs ($22.53 vs $4.61, respectively). This is consistent with the findings from Jit et al, who noted that different vaccine prices contributed to variability in CEA results [8]. Using a meta-regression approach and accounting for the drivers of variability in cost-effectiveness, our estimates are $1,714 per DALY averted (95% UI: $267 - $6,306) in Equatorial Guinea.

Our approach also facilitates decision-making for policy-makers in a country equipped with multiple estimates. For example, Bangladesh’s 19 published estimates ranged from $23 to $1,543 per DALY averted owing to differences in modeling assumptions that cannot be easily synthesized in a policy-making setting. Our approach quantifies the effects of important drivers of variability in ICERs, producing an estimated ICER and simple linear equation that allows policy-makers to leverage the estimated effects and apply them using their own assumptions about levels of coverage, efficacy, discount rates, and other factors included in our model. Under our model, the estimated ICER for Bangladesh is $343 per DALY averted (95 %UI: $52 - $1,235).

Our results provide encouraging evidence in favor of introducing rotavirus vaccination worldwide. All countries had predicted mean ICERs below three times GDP per capita, with the exception of the United States and Somalia. Taking uncertainty into account, the upper bound of the UI of the predicted ICER was below one times GDP per capita in 64 countries, but exceeded three time GDP per capita in five countries (the US, UK, Portugal, Greece, and Somalia).

Nevertheless, much progress needs to be made in order to meet the Global Vaccine Action Plan’s target of 90% coverage by 2030. Of the 195 countries considered here, only 24 had reached 90% coverage in 2017 [20]. Twelve countries (Zambia, Eritrea, Senegal, The Gambia, Uzbekistan, Sao Tome and Principe, Ghana, Burkina Faso, Mozambique, Burundi, Rwanda, and Nicaragua) are LICs, two (Palestinian Territories and Morocco) are LMICs, five (Armenia, Fiji, Guyana, Mauritius, Paraguay) are UMICs, and five (Luxembourg, Norway, Saudi Arabia, Bahrain, and Qatar) are HICs. These countries all have different economies but a shared commitment to rotavirus vaccination, suggesting that the large scale-up and delivery of vaccinations across the rest of the world is possible.

Our work here has a number of limitations. First, we are unable to account for all differences between the studies used in our meta-analysis. Doing so would require more detailed descriptions of methodologies used in each article. Second, although we used the full sample of results to estimate the probability that the result was cost-saving, and adjusted all predicted ICERs by the predicted cost-saving probability, we did not propagate the correlation between the logistic regression and the meta-regression models. The probability that the rotavirus vaccine was cost-saving at current vaccine prices was low however, meaning that the correlation would not have substantially changed our results. Third, we applied Lasso in Stage 3 of the analysis framework to identify essential features from a subset instead of all covariates. Future research may improve the estimates by extracting sensitivity analyses for a more covariates, applying selection criteria to the variables in Stage 1, or improving methods for variable selection in the presence of collinearity in Stage 3. Fourth, we did not assess validity of our predictions in terms of model calibration and discrimination. CEA researchers working with decision analytic models have initiated research on predictive validity, and this field will have implications for the quality of the published results that are the data for in our meta-regression analysis. Fifth, uncertainty of reported ICERs is largely based on sensitivity analyses of different assumed values for input parameters.

Despite these limitations, our work is the most comprehensive set of country-specific estimates of rotavirus vaccination. In producing these estimates, our meta-regression approach synthesizes all available evidence, quantifies uncertainty to facilitate decision-making, and incorporates country-specific estimates of rotavirus vaccine efficacy. Our results support introducing or expanding rotavirus vaccination, which remains a critical step in reducing rotavirus burden.

## Author contributions

MMJ, MRW, and CJLM led the design of the study. JJ, DM, LE, KLR, PPV, KC, and LS contributed to the design. DM, KLR, and GWS extracted the data. SBA and HHK provided vaccine efficacy estimates. PZ and AA developed the statistical modeling framework. MMJ and JJ conducted the statistical analysis. MMJ, JJ, DM, and LE produced figures and tables. CJLM and MRW secured funding for the study.

## Funding source

Bill and Melinda Gates Foundation (OPP1152504)

## Declaration of Competing Interest

The authors declare that they have no known competing financial interests or personal relationships that could have appeared to influence the work reported in this paper.
