# Supplementary material for: Cost-effectiveness of rotavirus vaccination in children under five years of age in 195 countries: A meta-regression analysis
Source: Vaccine. 2022 Jun 21;40(28):3903–17. doi: 10.1016/j.vaccine.2022.05.042 (PMC9208428; doi:10.1016/j.vaccine.2022.05.042)
Supplement: Supplementary data 1 [file mmc1.docx]

Supplementary Material for “Cost-effectiveness of Rotavirus vaccination in children under five years of age in 195 countries: A meta-regression analysis”

Contents

[Foreword: Disclosure 3](#_Toc95330071)

[Disclosure Reference: 3](#_Toc95330072)

[Section 1. Summary of articles included in analysis 4](#_Toc95330073)

[Section 1 References 18](#_Toc95330074)

[Section 2: GATHER Compliance 22](#_Toc95330075)

[Section 3. Intervention Taxonomy: 24](#_Toc95330076)

[Section 3.1: Intervention Taxonomy Overview 24](#_Toc95330077)

[Section 3.2. Guiding principles for intervention taxonomy 24](#_Toc95330078)

[Section 3 References: 27](#_Toc95330079)

[Section 4. Data extractions and mapping [1] 28](#_Toc95330080)

[Section 4.1: Null comparator 28](#_Toc95330081)

[Section 4.2: Time horizon 28](#_Toc95330082)

[Section 4.3: Discount rate 28](#_Toc95330083)

[Section 4.4: Age 28](#_Toc95330084)

[Section 4.5: Sex 29](#_Toc95330085)

[Section 4.6: Causes 29](#_Toc95330086)

[Section 4.7: Locations 29](#_Toc95330087)

[Section 4.8: Delivery platforms 29](#_Toc95330088)

[Section 4.9: Efficacy 31](#_Toc95330089)

[Section 4.10: Vaccine Price 31](#_Toc95330090)

[Section 4 References 32](#_Toc95330091)

[Section 5. Meta-regression analysis 33](#_Toc95330092)

[5.1. Crosswalk analyses of sensitivity analysis covariates 33](#_Toc95330093)

[5.2. Estimation of Nonlinear log-GDP per capita response curve 35](#_Toc95330094)

[5.2.1. B-splines linear tails 35](#_Toc95330095)

[5.2.2 Robust Trimming Strategy 36](#_Toc95330096)

[5.2.3 Spline Ensemble 36](#_Toc95330097)

[5.2.4 Sampling Knots from Simplex 36](#_Toc95330098)

[5.2.5 Scoring 37](#_Toc95330099)

[5.2.6 New nonlinear ‘signal’ covariate 37](#_Toc95330100)

[5.3. Covariate Selection 38](#_Toc95330101)

[5.4. Gaussian prior cross-validation 39](#_Toc95330102)

[5.5. Meta-Regression Analysis 39](#_Toc95330103)

[5.6. Selecting Efficacy Parameter 41](#_Toc95330104)

[Section 5 References 42](#_Toc95330105)

[Section 6: Cost-saving predictions 43](#_Toc95330106)

[Section 7. Vaccine Cost 44](#_Toc95330107)

[Section 7 References 45](#_Toc95330108)

[Section 8. Adjusted incremental cost-effectiveness ratios predicted from the model excluding efficacy. 46](#_Toc95330109)

# Foreword: Disclosure

Sections of this supplementary material have been adapted from Rosettie et al. [1]*.* Our goal as a research group is to provide evidence that will help stakeholders to make more informed decisions on adopting interventions with the best value, and to support delivery of those interventions through efficient health systems.

To build on available evidence, we are conducting meta-regression analyses of published CEAs in the Tufts University CEA registries to quantify the effects of factors at the method, intervention, and county-level, and generate estimates of incremental CE ratios (ICERs) for multiple interventions in 195 countries. Therefore, we have endeavored to standardize our approach and processes where possible and to improve our processes where necessary. Hence, much of the information contained herein is largely adapted from Rosettie et al. [1]*.*

At the beginning of each section, which largely bares resemblance to supplementary material of Rosettie et al. [1], there will be an appropriate citation should you be interested in retrieving previous information.

## Disclosure Reference

[1] Rosettie KL, Joffe JN, Sparks GW, Aravkin A, Chen S, Compton K, et al. Cost-effectiveness of HPV vaccination in 195 countries: A meta-regression analysis. PLOS ONE 2021;16:e0260808. https://doi.org/10.1371/journal.pone.0260808.

# Section 1. Summary of articles included in analysis

|  |  | | **Table S1. Selected characteristics of cost-effectiveness articles on rotatvirus vaccines included in the analysis** | | | | | | | | | | | | |  |
| --- | --- | --- | --- | --- | --- | --- | --- | --- | --- | --- | --- | --- | --- | --- | --- | --- |
| **Title** | | **Year** | | **Countries** | **Time Horizon** | **DALY/**  **QALY Discount Rate** | **Costs Discount Rate** | **Vaccine Types** | **PubMedID** | **Vaccine Cost (2017 I$)** | **Vaccine Efficacy** | **Vaccine Coverage (%)** | **Perspective** | **Number of ratios** | **Minimum ICER (2017 I$ per unit change in DALY or QALY)** | **Maximum ICER (2017 I$ per unit change in DALY or QALY)** |
| Health Care Costs of Diarrheal Disease and Estimates of the Cost-Effectiveness of Rotavirus Vaccination in Vietnam [1] | | 2005 | | Vietnam | 5 years | 3 | 3 | monovalent | 16235169 | 20.39 | 93 | 93 | healthcare payer | 1 | 178 | 178 |
| Potential Cost-Effectiveness of Vaccination for Rotavirus Gastroenteritis in Eight Latin American and Caribbean Countries [2] | | 2007 | | Argentina, Brazil, Chile, Dominican Republic, Honduras, Mexico, Panama, Venezuela | Lifetime | 3 | 3 | monovalent | 17612464 | Varies by country (134.17,  120.5, 100.13, 82.06, 75.85, 53.39, 80.11, 42.67) | 85 | Varies by country (88, 96, 99, 65, 92, 93, 86, 68) | limited societal | 8 | 438 | 22,961 |
| The Cost-Effectiveness of Rotavirus Vaccination in Australia [3] | | 2007 | | Australia | 5 years | 5 | 5 | monovalent and pentavalent | 18022735 | Varies by vaccine type (161.1, 181.24) | 85 – 95.8 | 90 | societal and payer | 4 | Cost-saving | 70,592 |
| Evaluating Rotavirus Vaccination in England and Wales. Part ii. The Potential Cost-Effectiveness of Vaccination [4] | | 2007 | | United Kingdom | Lifetime | 3.5 | 3.5 | monovalent and pentavalent | 17400341 | Varies by vaccine type (114.91, 123.12) | 94 | 95 | payer | 2 | 101,459 | 133,060 |
| Economic Impact of a Rotavirus Vaccine in Brazil [5] | | 2008 | | Brazil | Lifetime | 3 | 3 | monovalent | 19069617 | 37.66 | 85 | 96 | payer | 1 | 1,687 | 1,687 |
| The Cost-Utility of Rotavirus Vaccination with Rotarix (rix4414) in the Netherlands [6] | | 2008 | | Netherlands | Lifetime | 1.5 | 4 | monovalent | 18215445 | 102.33 | 100 | 100 | societal | 1 | 28,303 | 28,303 |
| Cost and Cost-Effectiveness of Childhood Vaccination Against Rotavirus in France [7] | | 2008 | | France | 3 years | 3 | 3 | monovalent and pentavalent | 18166250 | 192.29 | 85 | 75 | payer | 1 | 179,427 | 179,427 |
| Cost-Effectiveness of Rotavirus Vaccination: Exploring Caregiver(s) and "no medical care" Disease Impact in Belgium [8] | | 2008 | | Belgium | 7 years | 1.5 | 3 | monovalent and pentavalent | 18948433 | Varies by vaccine type (147.78, 146.46) | 96 - 98 | 97.5 | societal and payer | 4 | 10,126 | 87,950 |
| Cost-Effectiveness Analysis of Vaccination Against Rotavirus with rix4414 in France [9] | | 2008 | | France | Lifetime | 3 | 3 | monovalent | 19382820 | 146.14 | 100 | 85 | limited societal | 1 | 57,677 | 57,677 |
| Economic Evaluation of a Routine Rotavirus Vaccination Programme in Indonesia [10] | | 2009 | | Indonesia | 5 years | 3 | 3 | monovalent and pentavalent | 19931723 | 17.13 | 76.5 | 80 | payer | 1 | 151 | 151 |
| Cost-Benefit Analysis of a Rotavirus Immunization Program in the Arab Republic of Egypt [11] | | 2009 | | Egypt | 5 years | 3 | 3 | monovalent | 19817621 | 24.4 | 79.1 | 97 | payer | 1 | 539 | 539 |
| Rotavirus Disease Burden and Impact and Cost-Effectiveness of a Rotavirus Vaccination Program in Kenya [12] | | 2009 | | Kenya | Lifetime | 3 | 3 | monovalent | 19817618 | 4.5 | 85 | 75 | payer | 1 | 41 | 41 |
| Cost-Effectiveness of Rotavirus Vaccination in Peru [13] | | 2009 | | Peru | 5 years | 3 | 3 | monovalent | 19817591 | 12.17 | 85 | 97 | payer | 3 | 814 | 870 |
| Economic Impact of a Rotavirus Vaccination Program in Mexico [14] | | 2009 | | Mexico | 5 years | 3 | 3 | monovalent | 19695142 | 21.02 | 85 | 92.8 | payer | 1 | 1,027 | 1,027 |
| Cost-Effectiveness of Rotavirus Vaccination in Vietnam [15] | | 2009 | | Vietnam | 5 years | 3 | 3 | monovalent | 19159483 | 20.4 | 77 | 94 | societal | 1 | 1,058 | 1,058 |
| Budget Impact and Cost-Effectiveness of Including a Pentavalent Rotavirus Vaccine in the New Zealand Childhood Immunization Schedule [16] | | 2009 | | New Zealand | 10 years | 3.5 | 0 | pentavalent | 19490550 | 127.64 | 95 | 85 | societal and payer | 1 | 40,284 | 64,459 |
| Impact of Transmission Dynamics on the Cost-Effectiveness of Rotavirus Vaccination [17] | | 2009 | | United States | 20 years | 3 | 3 | pentavalent | 19389452 | 243.27 | 90 | 70 | societal and payer | 2 | 121,236 | 223,721 |
| Potential Impact and Cost-Effectiveness Analysis of Rotavirus Vaccination of Children in Israel [18] | | 2009 | | Israel | 5 years | 3 | 3 | monovalent and pentavalent | 19221026 | Varies by vaccine type (313.25, 337.34) | 85 – 92 | 94 | payer | 2 | 15,363 | 42,860 |
| The Cost-Effectiveness of Rotavirus Vaccination: Comparative Analyses for five European Countries and Transferability in Europe [19] | | 2009 | |  | 5 years |  |  |  | 19715781 |  |  |  | n/a only S.A. |  |  |  |
| The Cost-Effectiveness of Rotavirus Vaccination in Malawi [20] | | 2010 | | Malawi | 2 years | 3 | 3 | monovalent | 20684689 | 0.23 | 49.5 | 87 | payer | 1 | 4 | 4 |
| Cost-Effectiveness of Rotavirus Vaccination as Part of the National Immunization Program for Thai Children [21] | | 2010 | | Thailand | 5 years | 3 | 3 | monovalent | 20578490 | 17.41 | 90 | 85 | limited societal | 1 | 130 | 130 |
| Health and Economic Impact of Rotavirus Vaccination in Gavi-Eligible Countries. [22] | | 2010 | | Angola, Benin, Burkina Faso, Cameroon, Chad, Comoros, Ghana, Guinea, Guinea-Bissau, Liberia, Madagascar, Mali, Mauritania, Niger, Nigeria, Sao Tome and Principe, Senegal, Sierra Leone, The Gambia, Togo, Burundi,  Central African Republic, Congo, Cote D'Ivoire,  DR Congo, Eritrea, Ethiopia, Kenya, Lesotho, Malawi, Mozambique, Rwanda, Tanzania, Uganda, Zambia, Zimbabwe, Cuba, Guyana, Honduras, Bolivia, Haiti, Nicaragua, Afghanistan, Djibouti, Pakistan, Somalia, Sudan, Yemen, Armenia, Azerbaijan, Georgia, Kyrgyzstan, Tajikistan, Uzbekistan, Moldova, Ukraine, Indonesia, South Korea, Sri Lanka, Timor-Leste, Bangladesh, Bhutan, India, Myanmar, Nepal, Cambodia, Kiribati, Laos, Mongolia, Papua New Guinea, Solomon Islands, Vietnam | 5 years | 3 | 3 | monovalent and pentavalent | 20470426 | Varies by country (11.84, 6.55, 6.31, 6.88, 6.22, 8.92, 6.55, 6.74, 7.73, 8.81, 5.19, 6.71, 5.37, 6.79, 6.38, 10.12, 6.84, 5.98, 5.15, 6.62, 7.77, 10.27, 5.30, 7.48, 12.13, 11.33, 7.21, 8.86, 6.94, 5.01, 6.15, 6.81, 5.81, 5.45, 6.55, 9.43, 8.83, 10.54, 9.06, 7.97, 7.40, 6.91, 5.37, 10.10, 5.18, 5.41, 11.11, 10.16, 7.39, 4.33, 7.01, 5.62  4.74, 4.17, 7.14, 5.42, 5.67, 13.61, 5.85, 6.99, 6.95, 6.08, 4.91, 3.78, 5.61, 6.26, 15.59, 6.21, 5.28, 14.19, 17.59, 6.37) | Varies by country (78.4, 71.8, 84.7, 71.9, 72.9, 72.8, 81, 81.2, 73.2, 73.6, 80.8, 81.4. 75.6, 83.7, 81.9. 78.9, 75.8. 82.9, 83.5) | 70 | limited societal | 72 | 10 | 16,365 |
| Potential Epidemiological and Economical Impact of Two Rotavirus Vaccines in Colombia [23] | | 2010 | | Colombia | Lifetime | 3 | 3 | monovalent and pentavalent | 20347057 | Varies by vaccine type (26.54, 39.81) | 85 - 95 | 95 | societal | 2 | 1,232 | 2,587 |
| Is It Cost-Effective to Introduce Rotavirus Vaccination in the Dutch National Immunization Program? [24] | | 2010 | | Netherlands | 20 years | 1.5 | 4 | monovalent and pentavalent | 20109593 | Varies by vaccine type (112.39, 104.9) | 82 – 91 | 97 | societal | 2 | 61,818 | 68,126 |
| The Cost-Effectiveness of Rotavirus Vaccination in Armenia [25] | | 2011 | | Armenia | 13 years | 3 | 3 | monovalent | 21945959 | 7.92 | 79 | 95 | limited societal | 1 | 18 | 18 |
| Comparative Evaluation of the Potential Impact of Rotavirus Versus HPV Vaccination in Gavi-Eligible Countries: Preliminary Analysis Focused on the Relative Disease Burden [26] | | 2011 | | Angola, Benin, Burkina Faso, Cameroon, Chad, Comoros, Ghana, Guinea, Guinea-Bissau, Liberia, Madagascar, Mali, Mauritania, Niger, Nigeria, Sao Tome and Principe, Senegal, Sierra Leone, The Gambia, Togo, Burundi,  Central African Republic, Congo, Cote D'Ivoire,  DR Congo, Eritrea, Ethiopia, Kenya, Lesotho, Malawi, Mozambique, Rwanda, Tanzania, Uganda, Zambia, Zimbabwe, Cuba, Guyana, Honduras, Bolivia, Haiti, Nicaragua, Afghanistan, Djibouti, Pakistan, Somalia, Sudan, Yemen, Armenia, Azerbaijan, Georgia, Kyrgyzstan, Tajikistan, Uzbekistan, Moldova, Ukraine, Indonesia, South Korea, Sri Lanka, Timor-Leste, Bangladesh, Bhutan, India, Myanmar, Nepal, Cambodia, Kiribati, Laos, Mongolia, Papua New Guinea, Solomon Islands, Vietnam | 5 years | 3 | 3 | monovalent and pentavalent | 21679420 | Varies by country (7.90, 4.37, 4.21, 4.58, 4.15, 5.95, 4.37, 4.49, 5.16, 5.88, 3.46, 4.48, 3.58, 4.53, 4.25, 6.74, 4.56, 3.98, 3.43, 4.41, 5.18, 6.85, 3.53, 4.99, 8.09, 7.55, 4.80, 5.90, 4.63  3.34, 4.10, 4.54, 3.87, 3.63, 4.37, 6.29, 5.89, 7.02, 6.04, 5.32, 4.93, 4.61, 3.58, 6.73, 3.45, 3.61, 7.41, 6.77, 4.93, 2.89, 4.67, 3.74, 3.16, 2.78, 4.76, 3.61, 3.78, 9.07, 3.90, 4.66, 4.63, 4.05, 3.28, 2.52, 3.74, 4.17, 10.40, 4.14, 3.52,  9.46, 11.72, 4.25) | 85 | 70 | societal | 72 | Cost-saving | 6,539 |
| Projected Impact and Cost-Effectiveness of a Rotavirus Vaccination Program in India, 2008 [27] | | 2011 | | India | 5 years | 3 | 3 | monovalent | 21288839 | 2.04 | 50 | 63 | payer | 1 | 24 | 24 |
| Cost-Effectiveness of Rotavirus Vaccination in the Netherlands; the Results of a Consensus Model [28] | | 2011 | |  | 5 years |  |  |  | 21663620 |  |  |  | n/a only SA |  |  |  |
| Cost-Effectiveness Analysis of a Universal Rotavirus Immunization Program in Japan [29] | | 2011 | | Japan | 5 years | 5 | 5 | monovalent and pentavalent | 21788701 | 174.32 | 95 | 100 | societal and payer | 2 | 7,638 | 86,496 |
| Cost-Effectiveness of Universal Rotavirus Vaccination in Reducing Rotavirus Gastroenteritis in Ireland [30] | | 2011 | | Ireland | 5 years | 4 | 4 | monovalent and pentavalent | 21821085 | Varies by vaccine type (94.67, 59.18) | 97.3 – 100 | 90 | societal and payer | 3 | 92,010 | 150,910 |
| Cost-Effectiveness of a Pentavalent Human-Bovine Reassortant Rotavirus Vaccine for Children <=5 Years of Age in Taiwan [31] | | 2011 | | Taiwan | 5 years | 0 | 0 | pentavalent | 21919673 | 70.64, 81 | 98 | 96 | payer | 2 | 0 | 2,468 |
| Socio-Economic Modelling of Rotavirus Vaccination in Castilla y Leon, Spain [32] | | 2011 | | Spain | 5 years | 5 | 5 | monovalent and pentavalent | 22037437 | Varies by vaccine type (238.08, 213.9) | 85.2 – 94 | 100 | societal | 2 | 27,038 | 52, 638 |
| A Cost Effectiveness and Capacity Analysis for the Introduction of Universal Rotavirus Vaccination in Kenya: Comparison Between Rotarix and Rotateq Vaccines [33] | | 2012 | | Kenya | 5 years | 3 | 3 | monovalent and pentavalent | 23115650 | Varies by vaccine type (6.81, 14.29) | 78 | 86.4 | societal | 2 | 196 | 398 |
| Projected Health Impact and Cost-Effectiveness of Rotavirus Vaccination Among Children <5 Years of Age in China [34] | | 2012 | | China | Lifetime | 3 | 0 | monovalent | 22705174 | 3.56 | 72 | 93 | societal | 1 | Cost-saving | Cost-saving |
| Distributional Impact of Rotavirus Vaccination in 25 Gavi Countries: Estimating Disparities in Benefits and Cost-Effectiveness [35] | | 2012 | | Bangladesh, Congo, Ethiopia, India, Kenya, Niger, Nigeria, Uganda | 5 years | 3 | 3 | monovalent | 22520124 | Varies by country (6.70, 2.99, 7.51, 4.65, 6.73, 4.72, 4.19, 4.79) | 57 | Varies by Country (94.4, 60.6, 48.1, 69.1, 93.3. 49.1, 47.4, 80.8) | payer | 8 | 23 | 195 |
| Evaluation of Cost-Effectiveness of Live Oral Pentavalent Reassortant Rotavirus Vaccine Introduction in Ghana [36] | | 2012 | | Ghana | 5 years | 3 | 3 | pentavalent | 22321664 | 15.58 | 65 | 84.6 | payer | 1 | 64 | 64 |
| Health Economics of Rotavirus Immunization in Vietnam: Potentials for Favorable Cost-Effectiveness in Developing Countries [37] | | 2012 | | Vietnam | 5 years | 3 | 3 | pentavalent | 22119590 | 19.6 | 63.9 | 93 | societal and payer | 2 | 729 | 871 |
| Effectiveness and Cost-Effectiveness of Pediatric Rotavirus Vaccination in British Columbia: A Model-Based Evaluation [38] | | 2012 | | Canada | 5 years | 3 | 3 | monovalent and pentavalent | 23107595 | Varies by vaccine type (59.73, 32.9) | 95.8 – 100 | 93.2 | payer | 2 | 2,142 | 12,764 |
| Cost Effectiveness of Infant Vaccination for Rotavirus in Canada [39] | | 2012 | | Canada | 5 years | 5 | 5 | monovalent and pentavalent | 23730312 | Varies by vaccine type (178.49, 159.19) | 96 | 94 | societal and payer | 4 | Cost-saving | 120,875 |
| Cost-Effectiveness of a New Rotavirus Vaccination Program in Pakistan: a Decision Tree Model [40] | | 2013 | | Pakistan | 5 years | 3 | 3 | monovalent | 24176497 | 11.17 | 48.3 | 85 | payer | 1 | 165 | 165 |
| Cost-Effectiveness of Rotavirus Vaccination Programs in Taiwan [41] | | 2013 | | China | 5 years | 3 | 3 | monovalent and pentavalent | 24060569 | Varies by vaccine type (72, 108) | 96.1 | 96 – 97.8 | payer | 2 | 19,180 | 51,543 |
| An Update of "Cost-Effectiveness of Rotavirus Vaccination in the Netherlands: The Results of a Consensus Rotavirus Vaccine Model" [42] | | 2013 | | Netherlands | Lifetime | 1.5 | 4 | pentavalent | 23363553 | 88.35 | 92 | 95 | societal | 2 | 3,816 | 4.532 |
| Cost-Effectiveness of Rotavirus Immunization in Indonesia: Taking Breastfeeding Patterns into Account [43] | | 2013 | | Indonesia | 2 years | 3 | 3 | pentavalent | 23707163 | 12.27 | 76.5 | 94 | societal | 1 | 146 | 146 |
| Cost-Effectiveness of a Pentavalent Rotavirus Vaccine in Japan [44] | | 2013 | | Japan | 5 years | 3 | 3 | pentavalent | 23919721 | 154.51 | 88 | 94 | societal | 1 | 18,475 | 18,475 |
| Analysis of the Universal Immunization Programme and Introduction of a Rotavirus Vaccine in India with Indiasim [45] | | 2014 | | India | Lifetime | 3 | 3 | monovalent | 25091670 | 22.21 | 44 | 76.8 | other | 1 | 75 | 75 |
| Estimated Impact and Cost-Effectiveness of Rotavirus Vaccination in India: Effects of Geographic and Economic Disparities [46] | | 2014 | | India | 5 years | 3 | 3 | monovalent | 25091669 | 3.71 | 50 | Varies by region (82, 56.97, 66.88, 73.27,  82.19, 83.99,  79.8, 59.3, 50.6, 58.1) | other | 10 | 101 | 317 |
| Budget Impact and Cost-Utility Analysis of Universal Infant Rotavirus Vaccination in Spain [47] | | 2014 | | Spain | 5 years | 3 | 3 | pentavalent | 24360847 | 152.18 | 74 | 100 | societal | 1 | 242,646 | 242,646 |
| Cost-Effectiveness Analysis of Rotavirus Vaccination Among Libyan Children Using a Simple Economic Model [48] | | 2014 | | Libya | .75 years | 0 | 0 | pentavalent | 25499622 | 25.31 | 100 | 98 | limited societal | 1 | 8,667 | 8,667 |
| Cost Effectiveness of a Pentavalent Rotavirus Vaccine in Oman [49] | | 2014 | | Oman | 5 years | 0 | 0 | pentavalent | 24941946 | 50.51 | 88 | 94 | societal | 1 | Cost-saving | Cost-saving |
| Cost-Effectiveness of Childhood Rotavirus Vaccination in Germany [50] | | 2014 | | Germany | 5 years | 3 | 3 | monovalent and pentavalent | 24561052 | Varies by vaccine type (166.34, 166.65) | 96 – 98 | 90 | payer | 2 | 145,540 | 177,590 |
| Cost-Effectiveness of Rotavirus Vaccination for Under-Five Children in Iran [51] | | 2015 | | Iran | 5 years | 3 | 3 | monovalent and pentavalent | 26396704 | Varies by vaccine type (16.83, 11.22) | 84 | 98 | limited societal | 2 | 5,882 | 10,136 |
| The Cost Effectiveness of Rotavirus Vaccination in Iran [52] | | 2016 | | Iran | Lifetime | 3 | 3 | monovalent | 26360331 | 8.93 | 93 | 96.6 | payer | 1 | 17 | 17 |
| Cost-Effectiveness of Live Oral Attenuated Human Rotavirus Vaccine in Tanzania [53] | | 2015 | | Tanzania | 5 years | 3 | 3 | monovalent | 25949216 | 15.84 | 57 | 93 | payer | 1 | 42 | 42 |
| Epidemiological and Economic Impact of Monovalent and Pentavalent Rotavirus Vaccines in Low and Middle Income Countries: a Cost-Effectiveness Modeling Analysis [54] | | 2015 | | Afghanistan, Albania, Algeria, Angola, Malaysia, Samoa, Botswana, Brazil, Benin, Bhutan, Bolivia, Bosnia and Herzegovina, Bulgaria, Burkina Faso, Burundi, Cambodia, Cameroon, Cape Verde, Central African Republic, Chad, Chile, China, Cote D'Ivoire, Dominican Republic,  Colombia, Dr Congo, Georgia, Ghana, Guatemala, Guinea-Bissau, India, Jordan, Comoros, Kazakhstan, Kenya, Kyrgyzstan, Laos, Lesotho, Liberia, Macedonia, Malawi, Myanmar, Namibia, Nepal, Nicaragua, Niger, Nigeria, Pakistan, Panama, Papua New Guinea, Guyana, Paraguay, Peru, Philippines, Romania, Rwanda, Sierra Leone, Solomon Islands, Sri Lanka, Sudan, Swaziland, Syria, Zambia, Haiti, Tajikistan, Honduras, Congo, Costa Rica, Egypt, Eritrea, Ethiopia, Federated States of Micronesia, Fiji, Senegal, Guinea, Indonesia, Iraq, Mexico, Russia, Jamaica, Argentina, Venezuela, El Salvador, Gabon, Lithuania, Madagascar, Mali, Mauritania, Mauritius, Moldova, Mongolia, Morocco, Mozambique, Saint Lucia, Sao Tome and Principe, Tanzania, Thailand, The Gambia, Timor-Leste, Togo, Tonga, Tunisia, Turkey, Uganda, Ukraine, Uruguay, Vanuatu, Vietnam, Yemen, South Africa, Maldives, Armenia, Azerbaijan, Bangladesh,  Belarus, Belize | Lifetime | 3 | 3 | monovalent and pentavalent | 25923424 | Varies by Country and vaccine type (4.42, 6.18, 5.39, 4.21, 9.76, 5.08, 12.74, 7.44, 6.48, 9.23, 6.19, 5.40, 5.74, 5.01, 7.54, 6.57, 6.87, 5.99, 5.51, 5.97, 10.59, 5.20, 7.35, 6.41, 5.92, 5.16, 6.50, 5.67, 7.92, 6.90, 9.71, 6.32, 8.46, 5.88, 5.12, 10.38, 9.05, 8.91, 7.76, 6.16, 7.49, 6.53, 6.39, 11.46, 6.62, 5.77, 6.19, 5.39, 9.36, 6.37, 4.64, 4.05, 7.74, 8.43, 5.68, 4.95, 8.37, 7.30, 5.31, 4.63, 5.12, 5.87, 6.56, 5.72, 7.07, 8.33, 7.26, 6.23, 5.43, 4.13, 5.83, 3.12, 6.94, 4.62, 6.53, 9.99, 5.69, 6.42, 5.60, 6.03, 4.27, 4.90, 10.16, 8.86, 11.69, 13.41, 9.96, 7.39, 6.44, 8.59, 7.49, 5.42, 6.21, 7.46, 6.50, 6.43, 4.92, 8.68, 16.62, 14.49, 4.82, 10.50, 9.15, 6.59, 5.75, 3.69, 5.40, 5.30, 6.99, 4.24, 4.49, 7.46, 7.97, 7.35, 4.36, 11.84, 10.32, 2.80, 3.22, 6.10, 10.71, 9.34, 6.81, 5.94, 14.04, 8.70, 6.47, 5.64, 16.11, 6.37, 4.67, 4.46, 9.98, 8.16, 5.55, 7.31, 5.36, 5.12, 5.01, 8.02, 6.39, 9.51, 12.21, 1.96, 8.41, 7.03, 8.95, 6.99, 5.57, 10.65, 1.71, 7.33, 8.29, 6.13, 7.80, 6.75, 4.91, 4.73, 6.34, 5.53, 5.07, 4.42, 6.88, 6.75, 5.88, 4.35, 4.99, 5.46, 6.26, 5.81, 5.07, 3.58, 5.26, 5.61, 11.36, 9.90, 11.11, 9.56, 5.65, 3.91, 5.49, 4.78, 6.24, 5.44, 4.86, 4.24, 5.76, 6.60, 6.25, 5.45, 12.73, 11.10, 4.58, 5.25, 6.12, 7.02, 5.15, 4.49, 5.12, 4.46, 12.74, 11.11, 19.42, 16.93, 6.02, 5.25, 9.60, 8.37, 6.20, 4.83, 11.19, 5.07, 4.28, 8.33, 7.59, 8.56, 6.62, 7.33, 9.77, 11.20, 5.53, 6.99, 6.09, 4.10, 3.57, 6.57,  5.72, 5.16, 4.50, 9.87, 8.60) | Varies by vaccine (63, 87, 86, 57) | Varies by country (66, 99, 95, 91, 94, 87, 96, 98, 83, 80, 90, 92, 84, 54, 59, 85, 88, 63, 76, 72, 74, 64, 93, 82, 70, 69, 56, 97, 79, 89, 86, 57,  65, 78, 45, 60, 68) | payer | 231 | Cost-saving | 433,797 |
| Cost-Effectiveness of Rotavirus Vaccination in Albania [55] | | 2015 | | Albania | Lifetime | 3 | 3 | monovalent and pentavalent | 25919162 | Varies by vaccine type (14.66, 21.99) | 83 | 97.4 | societal | 2 | 458 | 3,149 |
| Cost-Effectiveness Analysis of the Introduction of Rotavirus Vaccine in Iran [56] | | 2015 | | Iran | Lifetime | 3 | 3 | monovalent and pentavalent | 25919160 | 20.75 | 82.1 | 99 | societal | 1 | 350 | 350 |
| Cost-Effectiveness Analysis of Rotavirus Vaccination in Argentina [57] | | 2015 | | Argentina | 5 years | 3 | 3 | monovalent and pentavalent | 25919152 | Varies by vaccine type (17.75, 18.28) | Varies by vaccine (87.6, 79.2) | Varies by vaccine (85, 95.8) | societal | 6 | Cost-saving | 2,152 |
| Estimated Impact and Cost-Effectiveness of Rotavirus vaccination in Senegal: A Country-led Analysis [58] | | 2015 | | Senegal | 5 years | 3 | 3 | monovalent | 25919151 | 1.62 | 59 | 94.7 | societal | 1 | 62 | 62 |
| Cost-Effectiveness of Rotavirus Vaccination in Kenya and Uganda [59] | | 2015 | | Kenya, Uganda | 5 years | 3 | 3 | monovalent | 25919149 | Varies by country (2.87, 1.18) | 67 | Varies by country (95, 100) | societal and payer | 2 | 23 | 44 |
| Comparison of Impact and Cost-Effectiveness of Rotavirus Supplementary and Routine Immunization in a Complex Humanitarian Emergency, Somali Case Study [60] | | 2015 | | Somalia | 1 year | 0 | 0 | monovalent | 25691915 | 0.32 | 50 | 47 | payer | 1 | 6 | 6 |
| Cost Effectiveness Evaluation of a Rotavirus Vaccination Program in Argentina [61] | | 2015 | | Argentina | Lifetime | 5 | 5 | monovalent and pentavalent | 26303875 | Varies by vaccine type (18.88, 21,24) | 85 | 90 | payer | 2 | Cost-saving | 1,874 |
| Cost-Effectiveness of Monovalent Rotavirus Vaccination of Infants in Malawi: A Postintroduction Analysis Using Individual Patient-Level Costing Data [62] | | 2016 | | Malawi | 20 years | 3 | 3 | monovalent | 27059360 | 0.37 | 64 | 86.3 | societal | 1 | 9 | 9 |
| Cost-Effectiveness of Rotavirus Vaccination in Ghana: Examining Impacts from 2012 to 2031 [63] | | 2017 | | Ghana | 20 years | 3 | 3 | monovalent and pentavalent | 29223486 | Varies by vaccine type (2.5, 4.55) | 65 | 93 | societal and payer | 4 | 270 | 373 |
| Potential Impact and Cost-Effectiveness of Rotavirus Vaccination in Afghanistan [64] | | 2017 | | Afghanistan | 10 years | 3 | 3 | monovalent | 29107346 | 4.04 | 53.1 | 77.3 | payer | 1 | 80 | 80 |
| Health Impact and Cost-Effectiveness of a Domestically-Produced Rotavirus Vaccine in India: A Model Based Analysis [65] | | 2017 | | India | 30 years | 3 | 3 | monovalent | 29099848 | 2.98 | 53.6 | 80 | limited societal | 1 | 58 | 58 |
| Cost-Effectiveness Analysis of Introducing Universal Childhood Rotavirus Vaccination in Bangladesh [66] | | 2017 | | Bangladesh | 2 years | 3 | 3 | monovalent | 29099653 | 2.16 | 55.1 | 65 | payer and limited societal | 2 | 811 | 824 |
| Impact and Cost-Effectiveness of Rotavirus Vaccination in Bangladesh [67] | | 2017 | | Bangladesh | 11 years | 3 | 3 | monovalent | 28623028 | 0.66, 4.46 | 38 – 58 | 94 | limited societal | 6 | 61 | 1,592 |
| Re-Evaluation of the Cost-Effectiveness and Effects of Childhood Rotavirus Vaccination in Norway [68] | | 2017 | | Norway | 5 years | 4 | 4 | monovalent and pentavalent | 28817621 | 58.09 | 93 | 94.7 | payer | 2 | 52,554 | 58,383 |

##

## Section 1 References

[1] Fischer TK, Anh DD, Antil L, Cat NDL, Kilgore PE, Thiem VD, et al. Health care costs of diarrheal disease and estimates of the cost-effectiveness of rotavirus vaccination in Vietnam. J Infect Dis 2005;192:1720–6. https://doi.org/10.1086/497339.

[2] Rheingans RD, Constenla D, Antil L, Innis BL, Breuer T. Potential cost-effectiveness of vaccination for rotavirus gastroenteritis in eight Latin American and Caribbean countries. Rev Panam Salud Publica 2007;21:205–16. https://doi.org/10.1590/s1020-49892007000300003.

[3] Newall AT, Beutels P, Macartney K, Wood J, MacIntyre CR. The cost-effectiveness of rotavirus vaccination in Australia. Vaccine 2007;25:8851–60. https://doi.org/10.1016/j.vaccine.2007.10.009.

[4] Jit M, Edmunds WJ. Evaluating rotavirus vaccination in England and Wales. Part II. The potential cost-effectiveness of vaccination. Vaccine 2007;25:3971–9. https://doi.org/10.1016/j.vaccine.2007.02.070.

[5] Constenla DO, Linhares AC, Rheingans RD, Antil LR, Waldman EA, da Silva LJ. Economic impact of a rotavirus vaccine in Brazil. J Health Popul Nutr 2008;26:388–96. https://doi.org/10.3329/jhpn.v26i4.1880.

[6] Goossens LMA, Standaert B, Hartwig N, Hövels AM, Al MJ. The cost-utility of rotavirus vaccination with Rotarix (RIX4414) in the Netherlands. Vaccine 2008;26:1118–27. https://doi.org/10.1016/j.vaccine.2007.11.070.

[7] Melliez H, Levybruhl D, Boelle PY, Dervaux B, Baron S, Yazdanpanah Y. Cost and cost-effectiveness of childhood vaccination against rotavirus in France. Vaccine 2008;26:706–15. https://doi.org/10.1016/j.vaccine.2007.11.064.

[8] Bilcke J, Van Damme P, Beutels P. Cost-effectiveness of rotavirus vaccination: exploring caregiver(s) and "no medical care’’ disease impact in Belgium. Med Decis Making 2009;29:33–50. https://doi.org/10.1177/0272989X08324955.

[9] Standaert B, Parez N, Tehard B, Colin X, Detournay B. Cost-effectiveness analysis of vaccination against rotavirus with RIX4414 in France. Appl Health Econ Health Policy 2008;6:199–216. https://doi.org/10.1007/BF03256134.

[10] Wilopo SA, Kilgore P, Kosen S, Soenarto Y, Aminah S, Cahyono A, et al. Economic evaluation of a routine rotavirus vaccination programme in Indonesia. Vaccine 2009;27 Suppl 5:F67-74. https://doi.org/10.1016/j.vaccine.2009.09.040.

[11] Ortega O, El-Sayed N, Sanders JW, Abd-Rabou Z, Antil L, Bresee J, et al. Cost-benefit analysis of a rotavirus immunization program in the Arab Republic of Egypt. J Infect Dis 2009;200 Suppl 1:S92-98. https://doi.org/10.1086/605057.

[12] Tate JE, Rheingans RD, O’Reilly CE, Obonyo B, Burton DC, Tornheim JA, et al. Rotavirus disease burden and impact and cost-effectiveness of a rotavirus vaccination program in kenya. J Infect Dis 2009;200 Suppl 1:S76-84. https://doi.org/10.1086/605058.

[13] Clark AD, Walker DG, Mosqueira NR, Penny ME, Lanata CF, Fox-Rushby J, et al. Cost-effectiveness of rotavirus vaccination in peru. J Infect Dis 2009;200 Suppl 1:S114-124. https://doi.org/10.1086/605043.

[14] Constenla D, Velázquez FR, Rheingans RD, Antil L, Cervantes Y. Economic impact of a rotavirus vaccination program in Mexico. Rev Panam Salud Publica 2009;25:481–90. https://doi.org/10.1590/s1020-49892009000600003.

[15] Kim S-Y, Goldie SJ, Salomon JA. Cost-effectiveness of Rotavirus vaccination in Vietnam. BMC Public Health 2009;9:29. https://doi.org/10.1186/1471-2458-9-29.

[16] Milne RJ, Grimwood K. Budget impact and cost-effectiveness of including a pentavalent rotavirus vaccine in the New Zealand childhood immunization schedule. Value Health 2009;12:888–98. https://doi.org/10.1111/j.1524-4733.2009.00534.x.

[17] Shim E, Galvani AP. Impact of transmission dynamics on the cost-effectiveness of rotavirus vaccination. Vaccine 2009;27:4025–30. https://doi.org/10.1016/j.vaccine.2009.04.030.

[18] Chodick G, Waisbourd-Zinman O, Shalev V, Kokia E, Rabinovich M, Ashkenazi S. Potential impact and cost-effectiveness analysis of rotavirus vaccination of children in Israel. Eur J Public Health 2009;19:254–9. https://doi.org/10.1093/eurpub/ckp005.

[19] Jit M, Bilcke J, Mangen M-JJ, Salo H, Melliez H, Edmunds WJ, et al. The cost-effectiveness of rotavirus vaccination: Comparative analyses for five European countries and transferability in Europe. Vaccine 2009;27:6121–8. https://doi.org/10.1016/j.vaccine.2009.08.030.

[20] Berry SA, Johns B, Shih C, Berry AA, Walker DG. The cost-effectiveness of rotavirus vaccination in Malawi. J Infect Dis 2010;202 Suppl:S108-115. https://doi.org/10.1086/653578.

[21] Chotivitayatarakorn P, Chotivitayatarakorn P, Poovorawan Y. Cost-effectiveness of rotavirus vaccination as part of the national immunization program for Thai children. Southeast Asian J Trop Med Public Health 2010;41:114–25.

[22] Kim S-Y, Sweet S, Slichter D, Goldie SJ. Health and economic impact of rotavirus vaccination in GAVI-eligible countries. BMC Public Health 2010;10:253. https://doi.org/10.1186/1471-2458-10-253.

[23] De la Hoz F, Alvis N, Narváez J, Cediel N, Gamboa O, Velandia M. Potential epidemiological and economical impact of two rotavirus vaccines in Colombia. Vaccine 2010;28:3856–64. https://doi.org/10.1016/j.vaccine.2010.03.004.

[24] Mangen M-JJ, van Duynhoven YTHP, Vennema H, van Pelt W, Havelaar AH, de Melker HE. Is it cost-effective to introduce rotavirus vaccination in the Dutch national immunization program? Vaccine 2010;28:2624–35. https://doi.org/10.1016/j.vaccine.2010.01.014.

[25] Jit M, Yuzbashyan R, Sahakyan G, Avagyan T, Mosina L. The cost-effectiveness of rotavirus vaccination in Armenia. Vaccine 2011;29:9104–11. https://doi.org/10.1016/j.vaccine.2011.08.127.

[26] Kim S-Y, Sweet S, Chang J, Goldie SJ. Comparative evaluation of the potential impact of rotavirus versus HPV vaccination in GAVI-eligible countries: a preliminary analysis focused on the relative disease burden. BMC Infect Dis 2011;11:174. https://doi.org/10.1186/1471-2334-11-174.

[27] Esposito DH, Tate JE, Kang G, Parashar UD. Projected impact and cost-effectiveness of a rotavirus vaccination program in India, 2008. Clin Infect Dis 2011;52:171–7. https://doi.org/10.1093/cid/ciq094.

[28] Rozenbaum MH, Mangen M-JJ, Giaquinto C, Wilschut JC, Hak E, Postma MJ, et al. Cost-effectiveness of rotavirus vaccination in the Netherlands; the results of a consensus model. BMC Public Health 2011;11:462. https://doi.org/10.1186/1471-2458-11-462.

[29] Sato T, Nakagomi T, Nakagomi O. Cost-effectiveness analysis of a universal rotavirus immunization program in Japan. Jpn J Infect Dis 2011;64:277–83.

[30] Tilson L, Jit M, Schmitz S, Walsh C, Garvey P, McKeown P, et al. Cost-effectiveness of universal rotavirus vaccination in reducing rotavirus gastroenteritis in Ireland. Vaccine 2011;29:7463–73. https://doi.org/10.1016/j.vaccine.2011.07.056.

[31] Itzler RF, Chen PY, Lac C, El Khoury AC, Cook JR. Cost-effectiveness of a pentavalent human-bovine reassortant rotavirus vaccine for children ≤5 years of age in Taiwan. J Med Econ 2011;14:748–58. https://doi.org/10.3111/13696998.2011.614303.

[32] Pérez-Rubio A, Luquero FJ, Eiros Bouza JM, Castrodeza Sanz JJ, Bachiller Luque MR, de Lejarazu RO, et al. Socio-economic modelling of rotavirus vaccination in Castilla y Leon, Spain. Infez Med 2011;19:166–75.

[33] van Hoek AJ, Ngama M, Ismail A, Chuma J, Cheburet S, Mutonga D, et al. A cost effectiveness and capacity analysis for the introduction of universal rotavirus vaccination in Kenya: comparison between Rotarix and RotaTeq vaccines. PLoS One 2012;7:e47511. https://doi.org/10.1371/journal.pone.0047511.

[34] Liu N, Yen C, Fang Z, Tate JE, Jiang B, Parashar UD, et al. Projected health impact and cost-effectiveness of rotavirus vaccination among children <5 years of age in China. Vaccine 2012;30:6940–5. https://doi.org/10.1016/j.vaccine.2012.05.084.

[35] Rheingans R, Atherly D, Anderson J. Distributional impact of rotavirus vaccination in 25 GAVI countries: estimating disparities in benefits and cost-effectiveness. Vaccine 2012;30 Suppl 1:A15-23. https://doi.org/10.1016/j.vaccine.2012.01.018.

[36] Abbott C, Tiede B, Armah G, Mahmoud A. Evaluation of cost-effectiveness of live oral pentavalent reassortant rotavirus vaccine introduction in Ghana. Vaccine 2012;30:2582–7. https://doi.org/10.1016/j.vaccine.2012.01.076.

[37] Tu H-AT, Rozenbaum MH, Coyte PC, Li SC, Woerdenbag HJ, Postma MJ. Health economics of rotavirus immunization in Vietnam: potentials for favorable cost-effectiveness in developing countries. Vaccine 2012;30:1521–8. https://doi.org/10.1016/j.vaccine.2011.11.052.

[38] Fisman DN, Chan CH, Lowcock E, Naus M, Lee V. Effectiveness and cost-effectiveness of pediatric rotavirus vaccination in British Columbia: a model-based evaluation. Vaccine 2012;30:7601–7. https://doi.org/10.1016/j.vaccine.2012.10.034.

[39] Coyle D, Coyle K, Bettinger JA, Halperin SA, Vaudry W, Scheifele DW, et al. Cost effectiveness of infant vaccination for rotavirus in Canada. Can J Infect Dis Med Microbiol 2012;23:71–7. https://doi.org/10.1155/2012/327054.

[40] Patel HD, Roberts ET, Constenla DO. Cost-effectiveness of a new rotavirus vaccination program in Pakistan: a decision tree model. Vaccine 2013;31:6072–8. https://doi.org/10.1016/j.vaccine.2013.10.022.

[41] Chang W-C, Yen C, Chi C-L, Wu F-T, Huang Y-C, Lin J-S, et al. Cost-effectiveness of rotavirus vaccination programs in Taiwan. Vaccine 2013;31:5458–65. https://doi.org/10.1016/j.vaccine.2013.08.103.

[42] Tu HAT, Rozenbaum MH, de Boer PT, Noort AC, Postma MJ. An update of “Cost-effectiveness of rotavirus vaccination in the Netherlands: the results of a Consensus Rotavirus Vaccine model.” BMC Infect Dis 2013;13:54. https://doi.org/10.1186/1471-2334-13-54.

[43] Suwantika AA, Tu HAT, Postma MJ. Cost-effectiveness of rotavirus immunization in Indonesia: taking breastfeeding patterns into account. Vaccine 2013;31:3300–7. https://doi.org/10.1016/j.vaccine.2013.04.055.

[44] Itzler R, O’Brien MA, Yamabe K, Abe M, Dhankhar P. Cost-effectiveness of a pentavalent rotavirus vaccine in Japan. J Med Econ 2013;16:1216–27. https://doi.org/10.3111/13696998.2013.831869.

[45] Megiddo I, Colson AR, Nandi A, Chatterjee S, Prinja S, Khera A, et al. Analysis of the Universal Immunization Programme and introduction of a rotavirus vaccine in India with IndiaSim. Vaccine 2014;32 Suppl 1:A151-161. https://doi.org/10.1016/j.vaccine.2014.04.080.

[46] Rheingans R, Anderson JD, Anderson B, Chakraborty P, Atherly D, Pindolia D. Estimated impact and cost-effectiveness of rotavirus vaccination in India: effects of geographic and economic disparities. Vaccine 2014;32 Suppl 1:A140-150. https://doi.org/10.1016/j.vaccine.2014.05.073.

[47] Imaz I, Rubio B, Cornejo AM, González-Enríquez J. Budget impact and cost-utility analysis of universal infant rotavirus vaccination in Spain. Prev Med 2014;61:116–21. https://doi.org/10.1016/j.ypmed.2013.12.013.

[48] Alkoshi S, Maimaiti N, Dahlui M. Cost-effectiveness analysis of rotavirus vaccination among Libyan children using a simple economic model. Libyan J Med 2014;9:26236. https://doi.org/10.3402/ljm.v9.26236.

[49] Al Awaidy ST, Gebremeskel BG, Al Obeidani I, Al Baqlani S, Haddadin W, O’Brien MA. Cost effectiveness of a pentavalent rotavirus vaccine in Oman. BMC Infect Dis 2014;14:334. https://doi.org/10.1186/1471-2334-14-334.

[50] Aidelsburger P, Grabein K, Böhm K, Dietl M, Wasem J, Koch J, et al. Cost-effectiveness of childhood rotavirus vaccination in Germany. Vaccine 2014;32:1964–74. https://doi.org/10.1016/j.vaccine.2014.01.061.

[51] Shakerian S, Moradi Lakeh M, Esteghamati A, Zahraei M, Yaghoubi M. Cost-Effectiveness of Rotavirus Vaccination for Under-Five Children in Iran. Iran J Pediatr 2015;25:e2766. https://doi.org/10.5812/ijp.2766.

[52] Mousavi Jarrahi Y, Zahraei SM, Sadigh N, Esmaeelpoor Langeroudy K, Khodadost M, Ranjbaran M, et al. The cost effectiveness of rotavirus vaccination in Iran. Hum Vaccin Immunother 2016;12:794–800. https://doi.org/10.1080/21645515.2015.1087626.

[53] Ruhago GM, Ngalesoni FN, Robberstad B, Norheim OF. Cost-effectiveness of live oral attenuated human rotavirus vaccine in Tanzania. Cost Effectiveness and Resource Allocation 2015;13:7. https://doi.org/10.1186/s12962-015-0033-0.

[54] Paternina-Caicedo A, De la Hoz-Restrepo F, Alvis-Guzmán N. Epidemiological and Economic Impact of Monovalent and Pentavalent Rotavirus Vaccines in Low and Middle Income Countries: A Cost-effectiveness Modeling Analysis. Pediatr Infect Dis J 2015;34:e176-184. https://doi.org/10.1097/INF.0000000000000727.

[55] Ahmeti A, Preza I, Simaku A, Nelaj E, Clark AD, Felix Garcia AG, et al. Cost-effectiveness of rotavirus vaccination in Albania. Vaccine 2015;33 Suppl 1:A201-208. https://doi.org/10.1016/j.vaccine.2014.12.075.

[56] Javanbakht M, Moradi-Lakeh M, Yaghoubi M, Esteghamati A, Mansour Ghanaie R, Mahmoudi S, et al. Cost-effectiveness analysis of the introduction of rotavirus vaccine in Iran. Vaccine 2015;33 Suppl 1:A192-200. https://doi.org/10.1016/j.vaccine.2014.12.035.

[57] Urueña A, Pippo T, Betelu MS, Virgilio F, Hernández L, Giglio N, et al. Cost-effectiveness analysis of rotavirus vaccination in Argentina. Vaccine 2015;33 Suppl 1:A126-134. https://doi.org/10.1016/j.vaccine.2014.12.074.

[58] Diop A, Atherly D, Faye A, Lamine Sall F, Clark AD, Nadiel L, et al. Estimated impact and cost-effectiveness of rotavirus vaccination in Senegal: A country-led analysis. Vaccine 2015;33 Suppl 1:A119-125. https://doi.org/10.1016/j.vaccine.2014.12.065.

[59] Sigei C, Odaga J, Mvundura M, Madrid Y, Clark AD, Kenya ProVac Technical Working Group, et al. Cost-effectiveness of rotavirus vaccination in Kenya and Uganda. Vaccine 2015;33 Suppl 1:A109-118. https://doi.org/10.1016/j.vaccine.2014.12.079.

[60] Gargano LM, Tate JE, Parashar UD, Omer SB, Cookson ST. Comparison of impact and cost-effectiveness of rotavirus supplementary and routine immunization in a complex humanitarian emergency, Somali case study. Confl Health 2015;9:5. https://doi.org/10.1186/s13031-015-0032-y.

[61] Martí SG, Alcaraz A, Valanzasca P, McMullen M, Standaert B, Garay U, et al. Cost effectiveness evaluation of a rotavirus vaccination program in Argentina. Vaccine 2015;33:5684–90. https://doi.org/10.1016/j.vaccine.2015.08.026.

[62] Bar-Zeev N, Tate JE, Pecenka C, Chikafa J, Mvula H, Wachepa R, et al. Cost-Effectiveness of Monovalent Rotavirus Vaccination of Infants in Malawi: A Postintroduction Analysis Using Individual Patient-Level Costing Data. Clin Infect Dis 2016;62 Suppl 2:S220-228. https://doi.org/10.1093/cid/civ1025.

[63] Nonvignon J, Atherly D, Pecenka C, Aikins M, Gazley L, Groman D, et al. Cost-effectiveness of rotavirus vaccination in Ghana: Examining impacts from 2012 to 2031. Vaccine 2018;36:7215–21. https://doi.org/10.1016/j.vaccine.2017.11.080.

[64] Anwari P, Debellut F, Pecenka C, Parwiz SM, Clark A, Groman D, et al. Potential impact and cost-effectiveness of rotavirus vaccination in Afghanistan. Vaccine 2018;36:7769–74. https://doi.org/10.1016/j.vaccine.2017.10.058.

[65] Rose J, Homa L, Meropol SB, Debanne SM, Bielefeld R, Hoyen C, et al. Health impact and cost-effectiveness of a domestically-produced rotavirus vaccine in India: A model based analysis. PLoS One 2017;12:e0187446. https://doi.org/10.1371/journal.pone.0187446.

[66] Sarker AR, Sultana M, Mahumud RA, Van Der Meer R, Morton A. Cost-effectiveness analysis of introducing universal childhood rotavirus vaccination in Bangladesh. Hum Vaccin Immunother 2018;14:189–98. https://doi.org/10.1080/21645515.2017.1356962.

[67] Pecenka C, Parashar U, Tate JE, Khan JAM, Groman D, Chacko S, et al. Impact and cost-effectiveness of rotavirus vaccination in Bangladesh. Vaccine 2017;35:3982–7. https://doi.org/10.1016/j.vaccine.2017.05.087.

[68] Edwards CH, Blasio BF de, Salamanca BV, Flem E. Re–evaluation of the cost–effectiveness and effects of childhood rotavirus vaccination in Norway. PLOS ONE 2017;12:e0183306. https://doi.org/10.1371/journal.pone.0183306.

# Section 2: GATHER Compliance

This study complies with the Guidelines for Accurate and Transparent Health Estimates Reporting (GATHER) recommendations. We have documented the steps involved in our analytical procedures and detailed the data sources used. See **Table S2** for the GATHER checklist. The GATHER recommendations may be found here: <http://gather-statement.org/>

| Table S2. GATHER compliance checklist. | | |
| --- | --- | --- |
| Item # | **Checklist item** | **Reported on page #** |
| Objectives and funding | | |
| 1 | Define the indicator(s), populations (including age, sex, and geographic entities), and time period(s) for which estimates were made. | Manuscript: Methods section |
| 2 | List the funding sources for the work. |  |
| Data Inputs | | |
| *For all data inputs from multiple sources that are synthesized as part of the study:* | | |
| 3 | Describe how the data were identified and how the data were accessed. | Manuscript: Methods section |
| 4 | Specify the inclusion and exclusion criteria. Identify all ad-hoc exclusions. | Manuscript: Methods section |
| 5 | Provide information on all included data sources and their main characteristics. For each data source used, report reference information or contact name/institution, population represented, data collection method, year(s) of data collection, sex and age range, diagnostic criteria or measurement method, and sample size, as relevant. | Supplemental Material: Table S1  Detailed information and links to all articles included in analysis can be found in Global Health Data Exchange, our online data sources tool. |
| 6 | Identify and describe any categories of input data that have potentially important biases (e.g., based on characteristics listed in item 5). | Manuscript: Methods, Results |
| *For data inputs that contribute to the analysis but were not synthesized as part of the study:* | | |
| 7 | Describe and give sources for any other data inputs. | N/A |
| *For all data inputs:* | | |
| 8 | Provide all data inputs in a file format from which data can be efficiently extracted (e.g., a spreadsheet rather than a PDF), including all relevant meta-data listed in item 5. For any data inputs that cannot be shared because of ethical or legal reasons, such as third-party ownership, provide a contact name or the name of the institution that retains the right to the data. | Detailed information and links to all articles included in analysis can be found in Global Health Data Exchange,. |
| Data analysis | | |
| 9 | Provide a conceptual overview of the data analysis method. A diagram may be helpful. | Manuscript: Methods section  Supplementary Material: Section 5 and 6 |
| 10 | Provide a detailed description of all steps of the analysis, including mathematical formulae. This description should cover, as relevant, data cleaning, data pre-processing, data adjustments and weighting of data sources, and mathematical or statistical model(s). | Manuscript: Methods section  Supplementary Material: Section 3-6 |
| 11 | Describe how candidate models were evaluated and how the final model(s) were selected. | Supplementary Material: Sections 5 and 6 |
| 12 | Provide the results of an evaluation of model performance, if done, as well as the results of any relevant sensitivity analysis. | Supplementary Material: Sections 5 |
| 13 | Describe methods for calculating uncertainty of the estimates. State which sources of uncertainty were, and were not, accounted for in the uncertainty analysis. | Supplementary Material: Sections 5 |
| 14 | State how analytic or statistical source code used to generate estimates can be accessed. | Analytical source code will be publically available on github. |
| Results and Discussion | | |
| 15 | Provide published estimates in a file format from which data can be efficiently extracted. | Files with published estimates will be publically available in a Global Health Data Exchange entry. |
| 16 | Report a quantitative measure of the uncertainty of the estimates (e.g. uncertainty intervals). | Manuscript: Table 2 and 3  Supplementary Material: Table S9 |
| 17 | Interpret results in light of existing evidence. If updating a previous set of estimates, describe the reasons for changes in estimates. | Manuscript: Discussion |
| 18 | Discuss limitations of the estimates. Include a discussion of any modelling assumptions or data limitations that affect interpretation of the estimates. | Manuscript: Discussion |

# Section 3. Intervention Taxonomy:

## Section 3.1: Intervention Taxonomy Overview

As previously reported in Rosettie et al [1] in order to retrieve relevant interventions from the Tufts University Cost Effectiveness Analysis (CEA) Registry, and the Global Health Cost Effectiveness (GHCEA) Registry we had to devise a searchable standard. The CEA registry includes a phrase known as the “intervention phrase” which describes the content of each entry. This variable is unstandardized. For example, phrases related to the Rotavirus Vaccine include:

- rotarix rotavirus vaccination program with gavi subsidy
- Rotavirus vaccine
- Rotavirus immunization
- rotavirtus vaccine(rotarix)

We reviewed existing intervention taxonomies to adapt for this analysis, including the World Health Organization International Classification of Health Interventions (ICHI), the ITAX taxonomy focused on capturing intervention features such as adaptability and mechanism of action [2], and Cochrane’s EPOC taxonomy [3]. After reviewing these existing taxonomies, we thought that they would not allow us to classify health interventions with sufficient detail to distinguish between drivers of ICERs. Therefore, we leveraged the IHME Global Health Data Exchange (GHDx) platform to develop an intervention taxonomy to standardize and categorize each intervention represented in the Tufts registries.

We used Open Refine to group similar interventions together using its natural language processing and text filtering tools. Previous iterations of this taxonomy utilized a component known as ‘intervention details’. The details represented attributes such as vaccine type, drug dosage, or target population. However that process has since been retired, and the intervention details are no longer utilized in the taxonomy. We created separate taxonomies for intervention keywords. Intervention keywords represented unique intervention components. For each term in the taxonomy, we added synonyms from each intervention description that were unique to that term to the taxonomy. These synonyms were pulled from the Tufts “Intervention Phrase” or “Intervention Paragraph” variables. In the case of the GHCEA registry the “Blurblong” column was also used to identify key variable components. When these variables were either too broad to add as synonyms or too vague to determine which keyword to assign them, we returned to the articles to extract additional details on the interventions and updated the intervention descriptions accordingly. We ran a SQL query to export all of the taxonomy terms and synonyms, and created a Python dictionary where the taxonomy terms were keys and the synonyms were values. We used this dictionary to map all of the ratios in the Tufts registries to one or more intervention keywords.

## Section 3.2. Guiding principles for intervention taxonomy

When building out the intervention taxonomy, we developed several guiding principles, including the following:

1. For pharmaceutical interventions, we chose to use the drug class as the most granular level of detail.
2. We excluded information about the specific target population (e.g. age, sex, risk group) and delivery platform from the intervention keywords, as we had processes for capturing these details in additional variables.
3. The majority of intervention keywords in our taxonomy will have one of two parents: (1) DALY gross interventions, or (2) QALY gross interventions. There are two exceptions to this structure, which necessitate adding a lower level to the taxonomy. These include the following:
   1. Polyhierarchy structure: For interventions that were shared across causes, we used a polyhierarchical approach wherein the same keyword had multiple parents (**Figure S1**). This was limited to chemotherapies and immunotherapies where the same drug classes were used to treat multiple causes, as well as imaging techniques that were used to screen for a variety of conditions. For example, “alkylating antineoplastic agents” had several parent keywords, such as “chemotherapy for biliary tract cancer” and “chemotherapy for B-cell lymphoma.”
   2. Variations on the level of detail provided for a given intervention: We added lower levels to the taxonomy when interventions were described using varying levels of detail in order to retain information on these distinctions (**Figure S2**). In these instances, every ratio would be mapped to the parent, and only those ratios with more detail would also be mapped to the lower level. For example, every ratio that involves antiplatelet therapy is mapped to the intervention keyword “antiplatelet therapy.” In addition, when articles specify the drugs they are evaluating, their corresponding ratios are also mapped to the drug classes for these drugs (e.g. “antiplatelet therapy with COX inhibitors).
4. If a particular intervention is used across multiple causes and the specific intervention did not differ by cause, we did not include the cause name in the intervention keyword. On the other hand, when the same category of intervention is across causes, yet the cause has a strong influence on the specifics of the intervention (e.g. dosage, timing, frequency), we included the cause name in the intervention keyword.


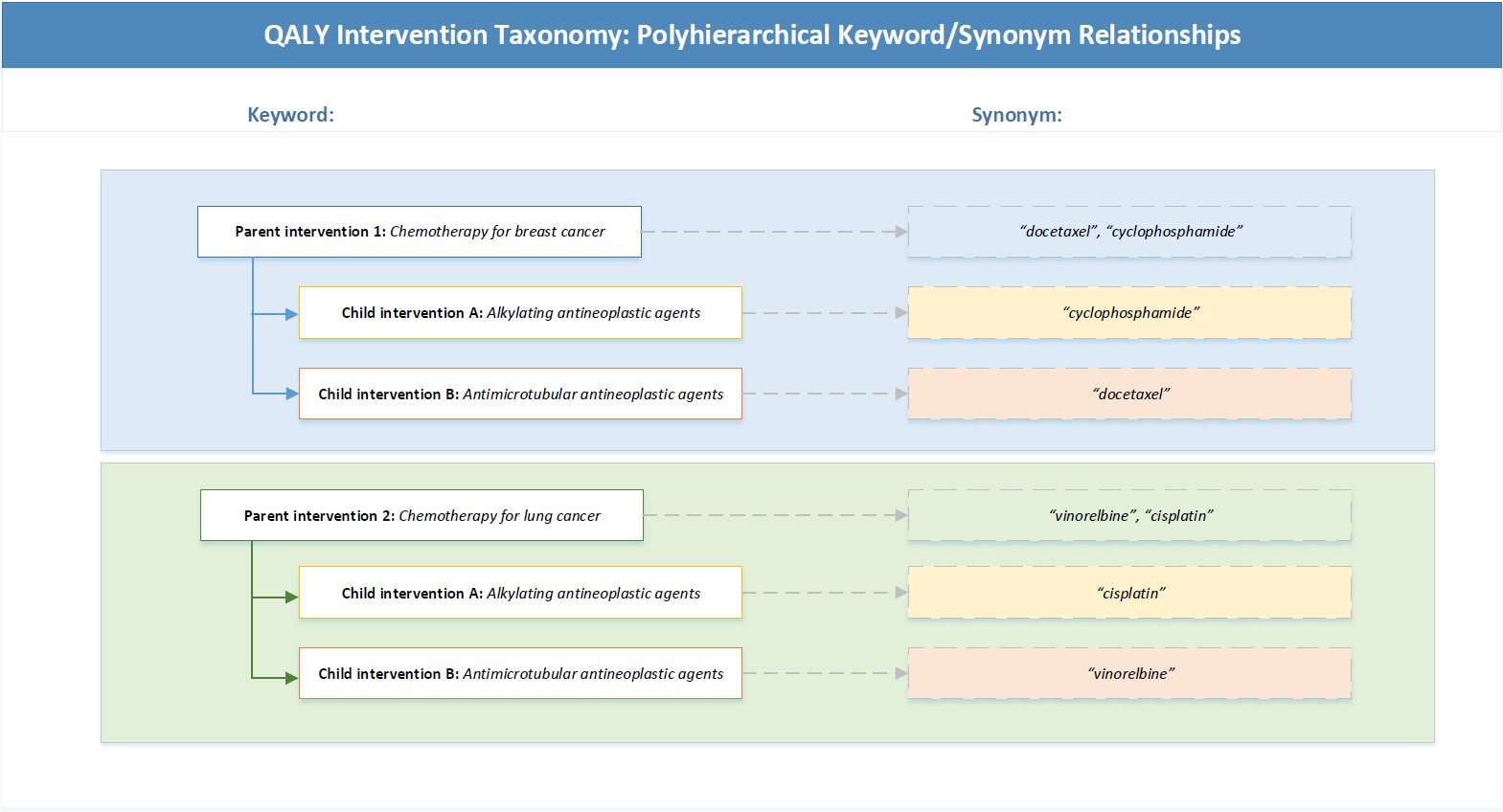


**Figure S1**. Polyhierarchical relationships in intervention taxonomy. Each parent intervention keyword has child keywords that represent specific drug classes for each type of chemotherapy. These drug classes are used to treat multiple cancer types, and thus have multiple parents. For example, “Alkylating antineoplastic agents” is a child with parents “Chemotherapy for breast cancer” and “Chemotherapy for lung cancer.” Therefore, interventions descriptions in the Tufts registries that contain the word “cisplatin” will be mapped to “Child intervention A” and “Parent intervention 2.” This allows us to meta-regress cost-effectiveness ratios for all chemotherapy interventions for a particular type of cancer (e.g. chemotherapy for breast cancer), or to meta-regress cost-effectiveness ratios across cancer types for a particular drug class (e.g. alkylating antineoplastic agents).


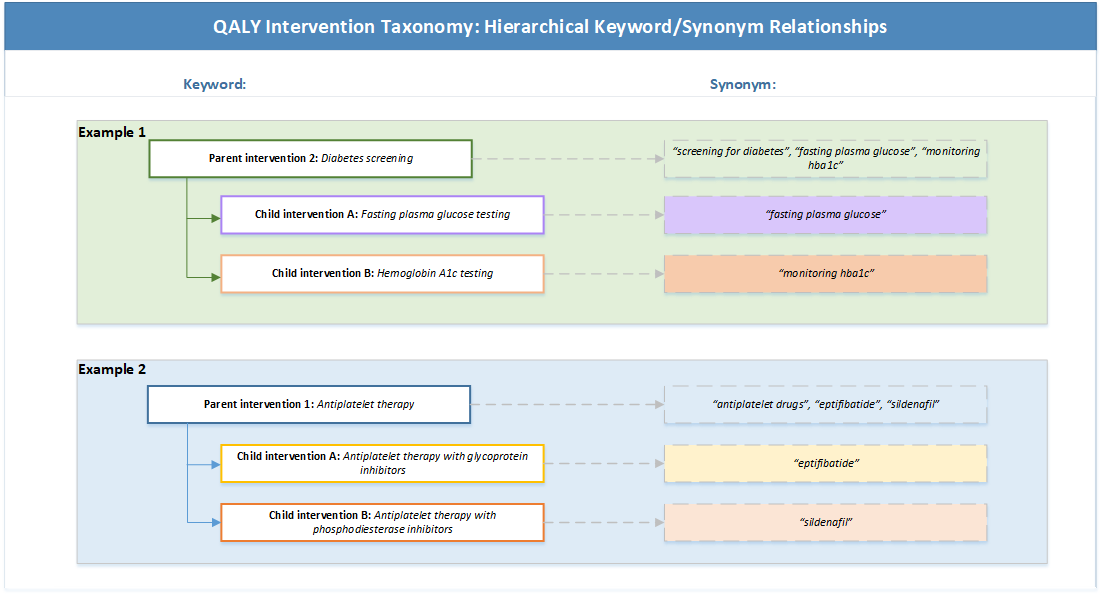


**Figure S2.** Hierarchical relationships in intervention taxonomy to accommodate varying levels of intervention detail. Articles describe interventions with varying levels of detail. In order to retain as much data as possible, we create parent-child relationships that allow for different degrees of intervention specificity. For example, when articles only describe interventions as “diabetes screening” without reference to the specific type of screening, we include synonyms for the parent “Diabetes screening” only. Interventions that include fasting plasma glucose testing or hemoglobin A1c testing are mapped to the parent and the more specific child. This allows us to run a regression for the parent intervention (e.g. all diabetes screening interventions), or to filter down to specific types of screening (e.g. all fasting plasma glucose testing interventions).

## Section 3 References

[1] Rosettie KL, Joffe JN, Sparks GW, Aravkin A, Chen S, Compton K, et al. Cost-effectiveness of HPV vaccination in 195 countries: A meta-regression analysis. PLOS ONE 2021;16:e0260808. https://doi.org/10.1371/journal.pone.0260808.

[2] Schulz R, Czaja SJ, McKay JR, Ory MG, Belle SH. Intervention Taxonomy (ITAX): Describing Essential Features of Interventions. American Journal of Health Behavior 2010;34:811–21. https://doi.org/10.5993/AJHB.34.6.15.

[3] EPOC Taxonomy n.d. https://epoc.cochrane.org/epoc-taxonomy (accessed November 17, 2021).

## Section 4. Data extractions and mapping [1]

## Section 4.1: Null comparator

The Tufts registries include a categorical variable for the comparator for each ICER, including none, placebo, standard of care, or other. They also included a text variable that described the comparator in more detail. When these data were missing, we returned to the articles to extract the comparator description and map the comparator to one of the aforementioned four categories.

When possible, we re-calculated ICERs compared to “other” such that they were compared to “null”. For articles that did not report sufficient data to re-calculate ICERs relative to no intervention, we re-calculated them compared to either “placebo” or “standard of care”. We used data in the Tufts registries on total or per person costs and total or per person health benefits. For ratios without these data reported in the registries, we went back to the articles to extract the necessary data to re-calculate the ICERs relative to the null comparator (i.e. “none”, “placebo”, or “standard of care”). We removed ratios that could not be re-calculated to the null comparator, as well as ratios that were compared to “other” if we had another ratio for the same intervention compared to the null.

We defined the null comparator as no intervention, standard of care, or placebo.

## Section 4.2: Time horizon

For ratios missing the time horizon variables in the Tufts registries, we returned to the papers to extract the time horizon magnitude and units (weeks, months, years, lifetime). For our analysis, we created a dichotomous variable: lifetime vs. not lifetime. If the article did not clearly state the time horizon, but the authors explicitly cite including mortality as an outcome of their analysis, we coded the corresponding ratios as having a lifetime time horizon. The time horizon was not missing for any ratios thus no ratios were omitted due to time horizon.

## Section 4.3: Discount rate

The Tufts registries include variables that capture the discount rate for costs and health outcomes (QALYs or DALYs). For those ratios missing discount rate data, we returned to the papers to extract these values. When articles referenced standard methods, we extracted the discount rates cited in those methods (e.g. WHO-CHOICE). We removed ratios from our analysis if the discount rates were not clearly stated in the article.

## Section 4.4: Age

The Tufts registries include several categorical variables for age. All tufts ratios had corresponding age variables.

## Section 4.5: Sex

For ratios missing sex information, we created a Python dictionary to map interventions to male, female, or both. For ratios that were not mapped using the Python dictionary approach, we used the Tufts’ target population descriptions to map ratios to sex.

## Section 4.6: Causes

In the Tufts registries, articles are mapped to one or more causes or risk factors, yet neither registry used the most recent GBD 2017 cause hierarchy. We needed each ratio to be mapped to one or more GBD 2017 causes, etiologies, or impairments in order to leverage the most recent GBD data. We first developed a mapping of Tufts causes to GBD 2017 causes. For articles with only one cause, we mapped all of the ratios in that article to that cause. For articles with multiple ratios, we first created a Python dictionary with cause-specific keywords to map each ratio to the most detailed cause(s) possible. Next, for those we could not map with the dictionary approach, we used Doctor Evidence software (<https://drevidence.com/>). We uploaded article titles to Doctor Evidence, which used a mapping algorithm to map keywords in those titles to ICD9 and ICD10 codes. We then used the ICD9 and ICD10 to GBD cause mapping algorithm to map these articles to GBD causes. Because these two processes (dictionary mapping and DRE) were automated, we validated them by reviewing the causes each ratio was mapped to and made changes to an erroneous mappings. For the remaining ratios that we were unable to map through the processes outlined above, we used the abstract or returned to the articles to map each of these ratios to GBD causes. Finally, we removed any ratios that could not be mapped to GBD causes.

## Section 4.7: Locations

The Tufts registries included a text variable for the target population country of the intervention. We merged this variable with the GBD location hierarchy. For locations that did not readily merge with GBD locations, we manually mapped them. For ratios that were mapped to regions or super-regions rather than countries, such as a series of WHO Choice articles, we returned to the articles to extract country-level ratios if they were present [2]. If there were no country-level results, we excluded these articles.

## Section 4.8: Delivery platforms

We mapped each ratio to one or more delivery platforms. We adapted the delivery platforms outlined in an article by Jamison and colleagues [2]. These included the following platforms: (1) intersectoral policies to reduce behavioral and environmental risks; (2) population-based; (3) community; (4) health facility; (5) first-level hospital; and (6) referral and specialty hospital. In addition, we separated the referral and specialty hospital platform into inpatient and outpatient, due to the differences in healthcare personal and infrastructure required for inpatient and outpatient services. When the delivery platforms were not clearly stated in the intervention descriptions or abstracts of the articles within the Tufts registries, we returned to the articles to determine the delivery platform. When the articles did not include delivery platform information, we used Jamison et al.’s categorization of interventions as guidance.[3] For interventions that were not represented in Jamison et al., we relied on domain knowledge for each intervention to map them to delivery platforms.

Definitions for the delivery platforms outlined by Jamison and colleagues and adapted for our analysis include the following:

**Platform 1**: **Intersectoral policies to reduce behavioral and environmental risks**

Definition: All policies outside of health sector

*Examples: (1) taxes on alcohol/tobacco; (2) trans-fat ban; (3) salt regulations with industry; (4) public transit; (5) traffic safety*

**Platform 2: Population-based**

Definition: this platform captures all non-personal or population-based health services, such as mass media and social marketing of educational messages, typically delivered by public health agencies.

*Examples: (1) mass media messages concerning awareness on handwashing; (2) sustained vector management for Chagas disease; (3) decentralize stocks of antiviral medications to improve access for at-risk populations; (4) ensure influenza vaccine security at national and sub-national level; (5) community-based HIV testing; (6) WASH behavior-change interventions (e.g. community-led sanitation)*

**Platform 3: Community**

Definition: the community platform encompasses efforts to bring health care services to clients, meeting people where they live. It includes a wide variety of delivery mechanisms. Specific sub platforms include: health outreach and campaigns ( e.g. vaccination campaigns, mass deworming, and face-to-face education and communication); schools (including school health days); and community health workers, who may be based primarily in the community but also connected to first-level care providers with ties to the rest of the system.

*Examples: (1) ANC and postpartum education on family planning; (2) counseling mothers on newborn care; (3) Childhood vaccines (rotavirus, pneumococcus, measles, hep B); (4) education on handwashing and safe disposal of children's stools*

**Platform 4: Health center**

Definition: the health center level captures two types of facility. The first is higher-capacity health facility staffed by a physician or clinical officer and often with a midwife to provide basic medical care, minor surgery, family planning and pregnancy services, and safe childbirth for uncomplicated deliveries. The second is a lower-capacity facility (e.g. health clinics, pharmacies, dental offices) staffed primarily by a nurse or mid-level health care provider, providing services in less-resourced and often more remote settings.

*Examples: (1) early detection and treatment of neonatal pneumonia; (2) hypertension screening; (3) PMTCT of HIV and syphilis; (4) partner notification and treatment for common STIs, including HIV; (5) PREP for HIV; (6) HIV screening in all individuals with TB; (7) providing aspirin for all cases of suspected acute MI; (8) tobacco cessation counseling; (9) screening and intervention for alcohol use disorders*

**Platform 5: First-level hospital**

Definition: a first-level hospital is a facility with the capacity to perform surgery and provide inpatient care. This platform also includes outpatient specialist care and routine pathology services that cannot be feasibly delivered at lower levels, such as newborn screening.

*Examples: (1) induction of labor post-term; (2) surgery for ectopic pregnancy; (3) tubal ligation; (4) calcium and vitamin D supplementation for osteoporosis secondary prevention; (5) medical management of acute heart failure; (6) hernia repair; (7) appendectomy; (8) basic skin grafting; (9) assisted vaginal delivery using vacuum extraction or forceps*

**Platform 6: Referral and specialty hospital outpatient**

Definition: this platform includes general specialists that provide secondary and tertiary services in an outpatient setting.

*Examples: (1) specialized TB services (managing MDR and XDR TB); (2) treatment of early stage cancers*

**Platform 7: Referral and specialty hospital inpatient**

Definition: this platform includes general specialists that provide secondary and tertiary services in an inpatient setting.

*Examples: (1) full supportive care for preterm newborns; (2) repair of cleft lip and palate*

## Section 4.9: Efficacy

The efficacy was not included in the Tufts registries and was extracted directly from each text in the articles. The columns ‘efficacy’ and ‘efficacy_desc’ were created to include these variables. Only one value for efficacy was extracted from each article and ‘efficacy_desc’ details the severity related to the efficacy. Values in ‘efficacy_desc’ include ‘all’, ‘hospitalization’, ‘death’, and ‘severe’. For articles where multiple efficacies were outlined, only the most severe efficacy was extracted. All ratios had corresponding efficacies.

## Section 4.10: Vaccine Price

The vaccine price extracted for each ratio was pulled through text in each article. Vaccine price is expressed in various ways across the articles and the variables ‘vaccine_cost_dose’, ‘vaccine_dose’, ‘vaccine_describe’, and vaccine_total_cost’ were incorporated in the data frame to extract the total vaccine cost. If the vaccine price is expressed in dosage, ‘vaccine_cost_dose’ equals the cost of each dose and ‘vaccine_doses’ equals the number of doses provided. The ‘vaccine_total_cost’ equals ‘vaccine_cost_dose’*’vaccine_doses’, or if the vaccine price is not expressed in doses, the ‘vaccine_total_cost’ is the total cost stated in the article. The last variable created, ‘vaccine_describe’ is a text variable with values ‘course’, ‘dose’, or ‘admin’. ‘Course’ is used when only the total vaccine cost is given, ‘dose’ is used when it clearly states the number of doses used for the vaccine, and ‘admin’ is used when administrative costs cannot be excluded from the vaccine price. All ratios had corresponding vaccine prices.

Another important aspect to vaccine price is the currency, as well as the currency year. The variables ‘vaccine_currency’ and ‘vaccine_currency_year’ were created to be able convert all vaccine prices to 2017 international dollars.

## Section 4 References

[1] Rosettie KL, Joffe JN, Sparks GW, Aravkin A, Chen S, Compton K, et al. Cost-effectiveness of HPV vaccination in 195 countries: A meta-regression analysis. PLOS ONE 2021;16:e0260808.

[2] Evans DB, Adam T, Edejer TT-T, Lim SS, Cassels A, Evans TG. Time to reassess strategies for improving health in developing countries. BMJ 2005;331:1133–6. https://doi.org/10.1136/bmj.331.7525.1133.

[3] Jamison DT, Alwan A, Mock CN, Nugent R, Watkins D, Adeyi O, et al. Universal health coverage and intersectoral action for health: key messages from Disease Control Priorities, 3rd edition. The Lancet 2018;391:1108–20. https://doi.org/10.1016/S0140-6736(17)32906-9.

#

# Section 5. Meta-regression analysis

This section details the statistical model and fitting procedure used to obtain the estimated ICERs, posterior uncertainty estimates, and other intermediate quantities as previously reported [1}. For technical details on model fitting please see [2]. Our meta-regression analysis is broken up into five stages. First, we conduct crosswalk analyses of seven covariates in order to leverage the sensitivity analyses reported in the studies from the Tufts registries, as described in Section 5.1. In the second stage, we estimate a nonlinear response curve for log-GDP per capita, as detailed in Section 5.2. This analysis uses splines to represent the curve, nonlinear observation models for relative risks, a robust statistical approach for outlier detection, and a spline ensemble to make the model less sensitive to model specification. Grouping is ignored in this stage of the analysis. In the third stage, we use the nonlinear response curve estimated in the second stage to select potential bias covariates using a generalized Lasso approach for linear mixed effects models, detailed in Section 5.3. In the fourth stage, as described in Section 5.4, we use 10-fold cross-validation to select the standard deviation of a Gaussian prior to apply to all covariates other than those analyzed in the crosswalk analysis (the first stage). In the fifth stage, we include covariates that were detected in the third stage, along with the nonlinear response, and consider a mixed effects model with a random intercept, as discussed in Section 5.5. Section 5.6 presents our experiments with the efficacy parameter and criteria for selecting the parameter used in the main results.

## 5.1. Crosswalk analyses of sensitivity analysis covariates

Univariate sensitivity analyses are used to estimate the effect of a variable on the ICER with all other variables held constant by definition. We analyzed the difference in log-ICERs between sensitivity analyses and the corresponding reference analyses using models which we refer to as crosswalks. Including the results of these models as priors in subsequent steps of the analysis decreases omitted variable bias by giving more influence to pairs of ICERs which we know differ in no unmeasured variables. It also stabilizes estimates in the presence of multicollinearity. See the correlation matrix in Table S3 below.

We conducted crosswalk analyses for seven variables: vaccine cost, cost discount rate, DALY/QALY discount rate, coverage, vaccine type (pentavalent), perspective (payer vs societal, limited societal, and sector combined), and vaccine efficacy. These covariates had a sufficient number of sensitivity analyses for us to control for study-specific variables not included in our model by using comparisons of sensitivity analyses and main results. For each of these covariates, we paired each sensitivity analysis with another ICER from the same study and location and which differed only in that covariate. For these covariates, we fit separate models of the form

$y_{i,sens}- y_{i,ref}= \alpha_{c}\times\left( x_{c_{i},sens}- x_{c_{i},ref} \right)+ \epsilon_{i}$ (1)

Where $x_{c}$ denotes the crosswalk covariate of interest, which is the only covariate that differs between the sensitivity (sens) and reference (ref) analyses, $y_{i,sens}$ and $y_{i,ref}$ represent the log-ICERs for sensitivity analysis $i$ and its corresponding reference analysis, while $x_{c_{i},sens}$ and $x_{c_{i},ref}$ are the respective values of $x_{c}$. Finally, $\epsilon_{i}\sim N(0,\sigma_{c}^{2})$ are iid normal errors for each crosswalk analysis.

The regression coefficients $\alpha_{c}$, their corresponding standard errors, $\hat{SE}\left[ \hat{\alpha}_{c} \right]$, and samples sizes for the estimates are reported in Table S4. Note that some sensitivity analyses were used in more than one crosswalk model. We use the coefficients and their standard errors as Gaussian priors in all subsequent model stages in which these covariates are included.

|  | Log ICER | Log GDP per capita (2017 USD) | Log Rotavirus DALYS per capita | Log Vaccine Cost (2017 USD) | Burden Measure | Vaccine Type (Pentavalent) | Vaccine Type (both) | Costs Discount Rate | Burden  Discount Rate | Vaccine Efficacy | Vaccine coverage | Payer Perspective | Not Lifetime |
| --- | --- | --- | --- | --- | --- | --- | --- | --- | --- | --- | --- | --- | --- |
| Log ICER | 1 |  |  |  |  |  |  |  |  |  |  |  |  |
| Log GDP per capita (2017 USD) | 0.87 | 1 |  |  |  |  |  |  |  |  |  |  |  |
| Log Rotavirus DALYS per capita | -0.86 | -0.87 | 1 |  |  |  |  |  |  |  |  |  |  |
| Log Vaccine Cost (2017 USD) | 0.86 | 0.81 | -0.72 | 1 |  |  |  |  |  |  |  |  |  |
| Burden Measure | 0.79 | 0.84 | -0.73 | 0.78 | 1 |  |  |  |  |  |  |  |  |
| Vaccine Type (Pentavalent) | 0.44 | 0.4 | -0.35 | 0.4 | 0.4 | 1 |  |  |  |  |  |  |  |
| Vaccine Type (both) | -0.63 | -0.56 | 0.54 | -0.58 | -0.44 | -0.48 | 1 |  |  |  |  |  |  |
| Costs Discount Rate | -0.05 | -0.04 | 0.05 | -0.07 | -0.05 | -0.04 | 0.08 | 1 |  |  |  |  |  |
| Burden  Discount Rate | -0.28 | -0.32 | 0.28 | -0.3 | -0.36 | -0.18 | 0.22 | 0.56 | 1 |  |  |  |  |
| Vaccine Efficacy | 0.6 | 0.66 | -0.66 | 0.63 | 0.6 | 0.22 | -0.25 | -0.03 | -0.25 | 1 |  |  |  |
| Vaccine coverage | 0.68 | 0.62 | -0.63 | 0.6 | 0.5 | 0.38 | -0.74 | -0.04 | -0.21 | 0.39 | 1 |  |  |
| Payer Perspective | 0.36 | 0.28 | -0.29 | 0.29 | 0.21 | 0.33 | -0.65 | 0.04 | -0.07 | 0.08 | 0.47 | 1 |  |
| Not Lifetime | -0.06 | 0.02 | 0.00 | -0.01 | 0.24 | -0.19 | 0.47 | 0.03 | -0.13 | 0.10 | -0.26 | -0.42 | 1 |
| **Table S3.** Correlation matrix of covariates included in meta-regression and logistic regression analyses. | | | | | | | | | | | | | |

| **Covariate** | $\hat{\boldsymbol{\alpha}_{\boldsymbol{c}}}$ | $\hat{\boldsymbol{SE}}\left[ {\hat{\boldsymbol{\alpha}}}_{\boldsymbol{c}} \right]$ | **Sample Size** |
| --- | --- | --- | --- |
| Log Vaccine Cost (2017 USD) | 0.733 | 0.014 | 426 |
| Costs Discount Rate | 0.044 | 0.026 | 12 |
| DALY/QALY Discount Rate | 0.117 | 0.016 | 55 |
| Coverage | -0.003 | 0.008 | 14 |
| Vaccine Type (Pentavalent) | 0.323 | 0.021 | 280 |
| Payer Perspective | 0.513 | 0.027 | 181 |
| Vaccine Efficacy | -0.024 | 0.001 | 284 |
| **Table S4. Parameter estimates for crosswalk analyses** | | | |

## 5.2. Estimation of Nonlinear log-GDP per capita response curve

The relationship between log-ICER and log-GDP per capita is modeled using a basis spline (B-spline)[3,4]. In this section, we present B-splines, specification of constraints, discussion of trimming, and summary of spline ensembles.

### 5.2.1. B-splines linear tails

A spline basis is a set of piecewise polynomial functions with designated degree and domain. If we denote polynomial order by $p$, and the number of knots by $k$, we need $p+ k$ basis elements $s_{j}^{p}$, which can be generated recursively as illustrated in Figure S3.

| 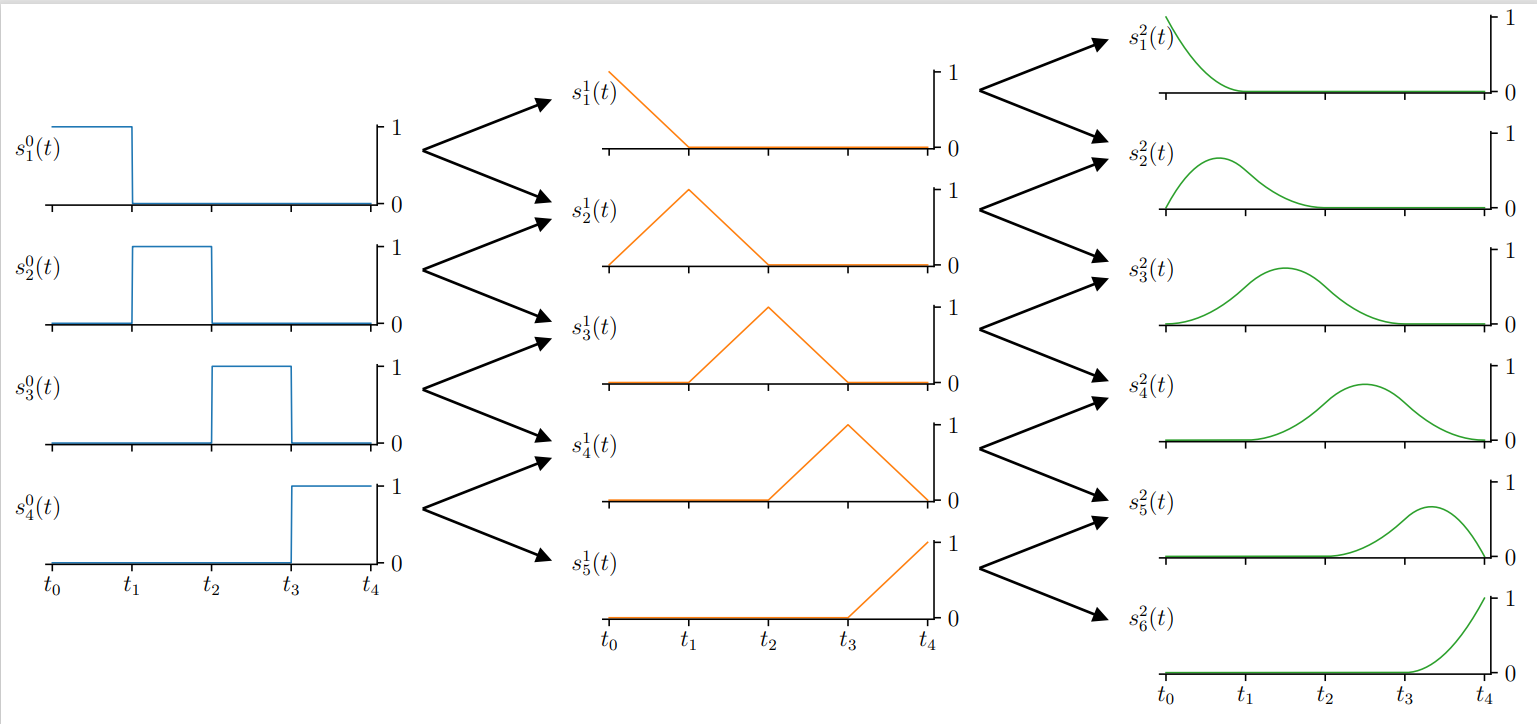 |
| --- |
| **Figure S3.** Recursive generation of b-spline basis elements (orders 0, 1, 2). |

Given such a basis, we can represent any curvilinear relationship as the linear combination of the spline basis elements, with coefficients $\beta\in\mathbb{R}^{p+k}$

$\boldsymbol{f}\left( \boldsymbol{t} \right)\boldsymbol{=}\sum_{\boldsymbol{j=1}}^{\boldsymbol{p+k}} \boldsymbol{\beta}_{\boldsymbol{j}}^{\boldsymbol{p}}\boldsymbol{s}_{\boldsymbol{j}}^{\boldsymbol{p}}\boldsymbol{(t)}$ (2)

An explicit representation of (2) is obtained by building a design matrix $\boldsymbol{X}$. Given a set of $t$ values at which we have data, the $j^{th}$ column of $\boldsymbol{X}$ is given by the expression:

$\boldsymbol{X}_{\boldsymbol{\cdot,j}}\boldsymbol{=}\left[ \begin{aligned} \boldsymbol{s}_{\boldsymbol{j}}^{\boldsymbol{p}}\left( \boldsymbol{0} \right) \\ \boldsymbol{\vdots} \\ \boldsymbol{s}_{\boldsymbol{j}}^{\boldsymbol{p}}\boldsymbol{(t)} \end{aligned} \right]$ (3)

For extreme values of log-GDP per capita with little data, we need the capability to ensure that the outermost segments of the spline are linear, with slopes that match the adjacent segment at the knot. Splines with linear tails are often called natural splines.

| 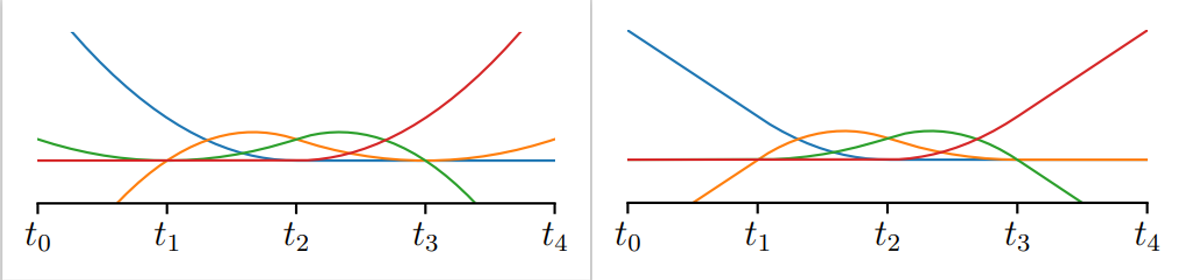 |
| --- |
| **Figure S4.** Left: spline bases. Right: spline bases with linear tails. |

### 5.2.2 Robust Trimming Strategy

To robustify the approach against outliers, we use the trimming strategy, as discussed in [2,5]. The estimator

$\min_{\boldsymbol{\beta\in B}} \sum_{\boldsymbol{i}} \boldsymbol{f}_{\boldsymbol{i}}\boldsymbol{(\beta)}$ (4)

Where $f_{i}$ is as in $(2)$ and $\boldsymbol{B}$ encodes all necessary constraints is extended to the ‘trimmed’ estimator

$\min_{\boldsymbol{w\in\Delta, \beta\in B}} \sum_{\boldsymbol{i}} \boldsymbol{w}_{\boldsymbol{i}}\boldsymbol{f}_{\boldsymbol{i}}\boldsymbol{(\beta)}$ (5)

where each $w_{i}$ is required to be between 0 and 1, and the total mass of $w$ is constrained to equal 90% of the data volume. Specifically, this means that

$\boldsymbol{\Delta=\{w:0 \leq}\boldsymbol{w}_{\boldsymbol{i}}\boldsymbol{\leq1,}\sum_{\boldsymbol{i}} \boldsymbol{w}_{\boldsymbol{i}}\boldsymbol{=0.9}\boldsymbol{N\}}$ (5)

Where $N$ is the total number of data points across studies. Thus the trimmed estimator finds the 90% most fittable data and fits them for β. Selecting a proportion of trimming has had a long history in terms of theory and recent methodological innovations [5,6]. However, thus far it has not been possible to automatically select the expected number of inliers. We chose 90% in order to include the vast majority of the data while remaining robust to a potential set of outliers. The same choice has been made in larger systematic analyses as well [7]. Alternative proportions are available, but we did not experiment with them.

### 5.2.3 Spline Ensemble

Every model estimate intrinsically depends on the choice of knot placement used to generate the spline. To remove the effect of this choice on the estimates, we develop an ensemble over this knot placement, leaving only the choice of spline degree and number of knots as modeling choices.

Given the degree and number of knots, we automatically sample a set of knot placements for a feasible knot distribution. For each resulting knot placement, we fit a spline (using the trimming estimator) and then evaluate each resulting model by computing its fit and curvature, aggregating the final model as a weighted combination of the ensemble.

### 5.2.4 Sampling Knots from Simplex

To establish a reasonable feasible set from which to sample, we prefix a minimal set of the rules for the knot-placement and uniformly sample from this feasible set. Given a number of knots, the rules specify feasible ranges for each knot, and feasible gaps between knots. Specifically, given an interval $[t_{0},t_{k}]$ delimited by terminal knots (which are always the minimum and maximum of the data), the feasible region of the interior knots $t_{1},\ldots, t_{k-1}$ is given by

$$t_{i}\in\left[ a_{i}, b_{i} \right], \mathrm{for} i= 1, \ldots, k-1 , t_{i}-t_{i-1}\in\left[ c_{i},d_{i} \right]\mathrm{for}i=1,\ldots, k.$$

We enforce the rules

$$a_{i}\geq t_{0}, b_{i}\leq t_{k}, c_{i}\geq0, \sum_{i} c_{i}\leq t_{k}-t_{0}.$$

The knot placement that satisfy these four rules comprise a closed polyhedron $\left\{ \boldsymbol{t}:\boldsymbol{Pt} \leq\boldsymbol{p} \right\}$, where,

$$\boldsymbol{P=}\left[ \begin{aligned} \boldsymbol{I} \\ \boldsymbol{-I} \\ \boldsymbol{D} \\ \boldsymbol{-D} \end{aligned} \right]\boldsymbol{, p=}\left[ \begin{aligned} \boldsymbol{b} \\ \boldsymbol{-a} \\ \boldsymbol{d} \\ \boldsymbol{-c} \end{aligned} \right]\boldsymbol{, D=}\left[ \begin{matrix} \boldsymbol{-1 1} & \boldsymbol{\cdots} & \boldsymbol{\ldots} \\ \boldsymbol{\vdots\vdots} & \boldsymbol{\ddots\ddots} & \boldsymbol{\vdots\vdots} \\ \boldsymbol{\ldots} & \boldsymbol{\cdots} & \boldsymbol{-1 1} \end{matrix} \right]\boldsymbol{, b=}\left[ \begin{aligned} b_{1} \\ \vdots\\ b_{k-1} \end{aligned} \right]\boldsymbol{, c=}\left[ \begin{aligned} c_{1} \\ \vdots\\ c_{k} \end{aligned} \right]\boldsymbol{, d=}\left[ \begin{aligned} d_{1} \\ \vdots\\ d_{k} \end{aligned} \right]$$

We calculate the vertices of the polyhedron using the double description method in [8], and uniformly sample knot-placements from within the polyhedron. Each knot placement yields a model, fit using the trimmed constrained spline approach described above.

### 5.2.5 Scoring

Once the ensemble is created, we score the resulting risk curves using two criteria: model fit (measured using the log-likelihood) and total variation (measured using the highest order derivative). These scores balance competing objectives of fit and generalizability. Once we have these scores, denoted as $s_{1}$ and $s_{2}$, we normalize them to the range $[0,1]$:

$$v_{i}= \frac{s_{i}-min(s_{i})}{\max\left( s_{i} \right)-min(s_{i})}$$

and apply a logistic transformation. The transformation is used to make the scoring meaningful even in the presence of spurious curves in a large ensemble. We then multiply the scores

$$\boldsymbol{w=}\boldsymbol{w}_{1}^{p_{1}}\boldsymbol{\odot}\boldsymbol{w}_{2}^{p_{2}}$$

to down-weight models that are low under either criterion (fit or total variation). The final weights are normalized to sum to 1.

### 5.2.6 New nonlinear ‘signal’ covariate

We fit a model of log-ICER on log-GDP per capita using a robust spine ensemble on log-GDP per capita with degree 2, two knots, and linear tails. This model also includes as covariates log cervical cancer DALYs per capita and the four crosswalk covariates. We placed Gaussian priors with means $\hat{\alpha}_{c}$ and standard deviation ${\hat{SE}[\hat{\alpha}}_{c}]$ on the crosswalk covariates’ coefficients. We used this model to generate a nonlinear log-GDP per capita response curve, which is encoded into a new nonlinear covariate called ‘signal’ and included in subsequent stages of the analysis. The shape of this transformation is displayed in Figure S5.

| 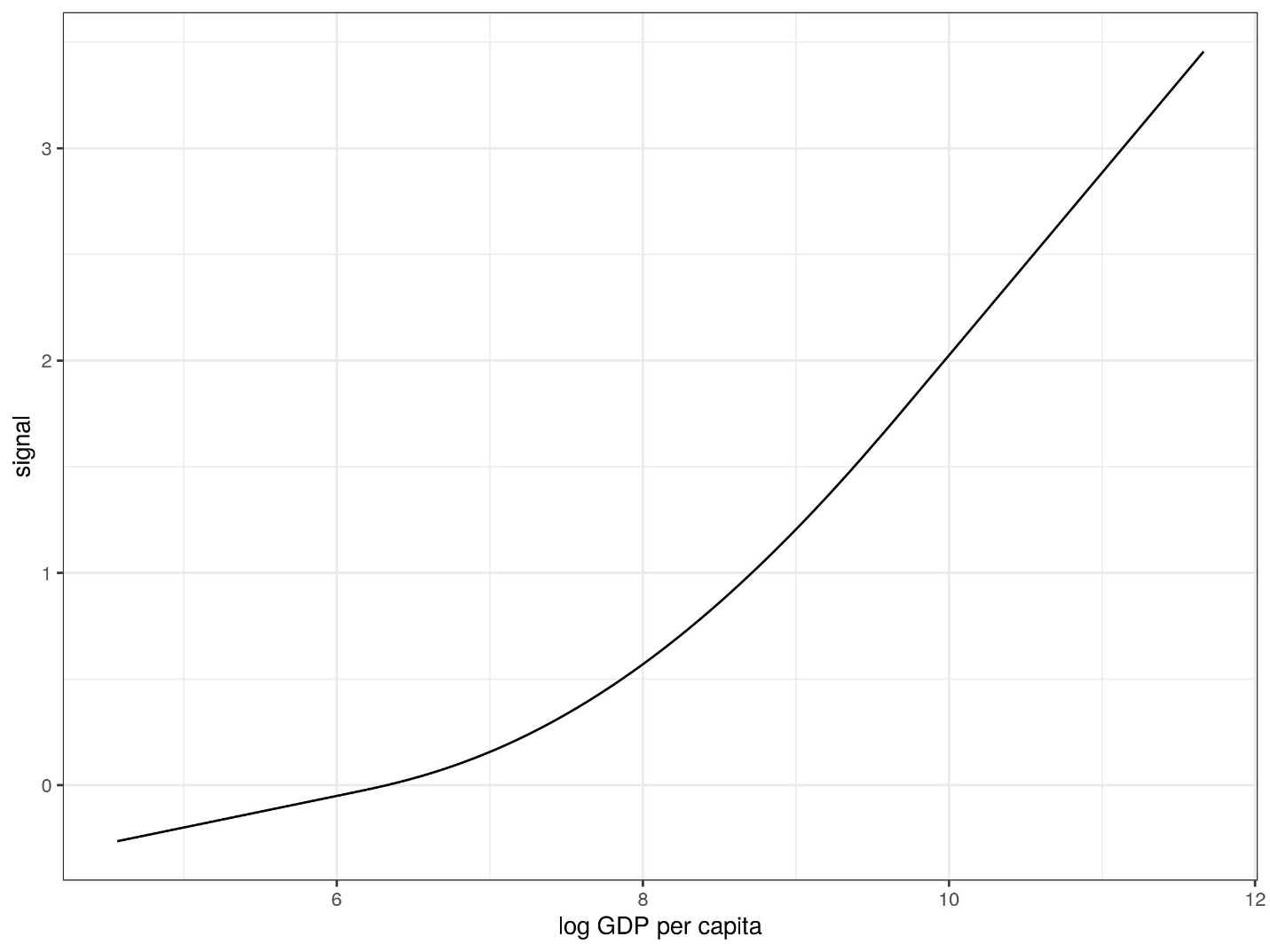 |
| --- |
| **Figure S5.** Nonlinear transformation of the log GDP per capita variable |

In particular, this allows us to fit a linear mixed effects model of the form:

$$y_{ij}= \beta_{0}+\mathrm{signal}_{\mathrm{ij}}\times\beta_{s}+x_{1ij}\beta_{1}+\ldots+ x_{kij}\beta_{k}+u_{i}+ \epsilon_{ij} (4)$$

Where $\epsilon_{ij}\sim N(0,\sigma_{ij}^{2})$ are known for each observation, and $u_{i}\sim N(0,\gamma)$ is a random study-specific intercept with unknown variance $\gamma$.

## 5.3. Covariate Selection

Additional covariates are selected using a Lasso stategy described below in the context of linear mixed effects models [9, 10]. In considering potential covariates, we enforce that every categorical covariate has some variation; in particular every indicator covariate has at least two studies in each category.

• We iteratively decrease the weight on the Lasso regularizer and let coefficients of bias-covariates enter the model in the order derived from the Lasso solutions.

• As a group of coefficients enters the model, we test it for statistical significance.

– If the coefficients are significant, we compute their posterior distribution and use this posterior as the prior for these coefficients for the next round.

– If the coefficients are not significant, the process terminates, and we return the list of (significant) covariates obtained so far.

We included the signal and the four crosswalk covariates as pre-selected covariates without the Lasso regularizer in all models above. We added Gaussian priors to the coefficients of the crosswalk covariates with mean $\hat{\alpha}_{c}$ and standard deviation ${\hat{SE}[\hat{\alpha}}_{c}]$, as estimated in Section 5.1.

Covariates with low variance or that are highly correlated with others are unlikely to be selected by this process, since including them would likely inflate the variance of the resulting estimators by an amount that outweighs the reduction in bias. This is a limitation of the current data set, and future work to expand the data set by extracting sensitivity analyses for a wider number of covariates could allow for the stable estimation of additional parameters.

There is ongoing methodological work to improve variable selection in the presence of collinearity. Based on early work showing the advantages of bridge regression vs. lasso [11] in the presence of correlation, the elastic net penalty [12] has been used, and in principle able to find groups of correlated predictors. Practical use requires additional parameter selection. We are currently looking into methods based on nonconvex regularizes as well [13]. These innovations can further improve variable selection in future work, but now we test for collinearity using basic tests before the lasso procedure starts.

One of the difficult questions in any variable selection procedure is when to stop. The methodology in step 1 builds on the Lasso methodology, but has an automatic termination criteria, stopping as soon as sequentially selected variables (selected across a range of the Lasso parameter) cease to be statistically significant in a standard Gaussian analytical framework.

Bias covariates that pass the selection process are included in the next stage of the model fitting.

## 5.4. Gaussian prior cross-validation

In order to further safeguard against overfitting, we included a Gaussian prior on all covariates. We used 10-fold cross validation to determine the prior standard deviation, $\tau_{cv}$, to apply to the coefficients of all covariates other than the four crosswalk covariates. We fit models of the same form as $(4)$ with priors on coefficients $\beta$. For the seven crosswalk covariates, we used the priors calculated in Section 5.1. For all others, we used a common $N\left( 0, \tau_{cv}^{2} \right)$ prior on their coefficients after standardizing the covariates to have mean 0 and unit variance. We used a grid-search to select the value of $\tau_{cv}$, that minimizes the MSE for predicting data in the hold-out set.

## 5.5. Meta-Regression Analysis

Once the signal covariate is obtained (Section 5.2), bias covariates are selected (Section 5.3), and priors are calculated for crosswalk (Section 5.1) and non-crosswalk (Section 5.4) covariates, we convert the priors on standardized covariates calculated in Section 5.4 to an unstandardized scale and fit a final model of the form

$$y_{ij}= \beta_{0}+x_{1ij}\beta_{1}+\ldots+ x_{kij}\beta_{k}+u_{i}+ \epsilon_{ij} (5)$$

where coefficients for crosswalk covariates have priors $\beta_{c}\sim N\left( \hat{\alpha}_{c}, \hat{SE}\left[ \hat{\alpha}_{c} \right]^{2} \right)$, as estimated in Section 5.1. Coefficients for all other covariates selected in Section 3, including the signal covariate, have priors $\beta_{l}\sim N\left( 0, \tau_{cv}^{2} \right)$, as selected in Section 5.4. $u_{j}\sim N\left( 0, \gamma\right)$ is a study-specific random intercept and $\epsilon_{ij}\sim N\left( 0, \sigma_{\epsilon}^{2} \right)$ are independent error terms. Parameter estimates are displayed in Table S5.

Parameters $\beta$ and $\gamma$ are estimated using maximum likelihood, as detailed in [2]. Standard errors of $\beta$ are estimated by taking the standard deviation across 1000 samples from the posterior distribution of  $\hat{\beta}.$

| **Covariate** | $\hat{\boldsymbol{\beta}}$ | $\hat{\boldsymbol{SE}}\boldsymbol{[}\hat{\boldsymbol{\beta}}\boldsymbol{]}$ | $\hat{\boldsymbol{\gamma}}$ |
| --- | --- | --- | --- |
| Intercept | 0.573 | 0.233 | 0.576 |
| Signal | 0.877 | 0.033 |  |
| Vaccine Type (both) | 0.256 | 0.267 |  |
| Vaccine Type (pentavalent) | 0.257 | 0.016 |  |
| Log Vaccine Cost | 0.640 | 0.009 |  |
| Burden Discount Rate | 0.109 | 0.010 |  |
| Costs Discount rate | 0.006 | 0.011 |  |
| Coverage | 0.021 | 0.001 |  |
| Payer Perspective | 0.427 | 0.020 |  |
| Efficacy | -0.016 | 0.001 |  |
| QALYs | 0.157 | 0.193 |  |
| Log Burden Variable | -0.417 | 0.008 |  |
| Not lifetime | -0.053 | 0.221 |  |
|  |  |  |  |
|  | R^2^ | RMSE | Sample Size |
| **Sample with Sensitivity Analyses** |  |  |  |
| Fixed and Random Effects | 0.96 | 0.61 | 1210 |
| Fixed Effects Only | 0.94 | 0.74 |  |
| Sample of Tufts Registry entries only |  |  |  |
| Fixed and Random Effects | 0.95 | 0.59 | 349 |
| Fixed Effects Only | 0.93 | 0.76 |  |
| **Table S5.** Parameter estimates and fit statistics for rotavirus meta-regression analysis. | | | |

## 5.6. Selecting Efficacy Parameter

We extracted efficacy from published studies to include in meta-regression analyses and test whether or not efficacy leads to better model fits. We considered a number of different ways to include efficacy: as a main effect, as effective coverage, and finally we considered excluding it entirely. Our final model is based on model fit. We defined effective coverage as the product of efficacy and coverage, scaled to be between 0 and 100%, under the assumption that the true efficacy of the vaccine in a population is also a function of its coverage due to herd effects. Model fit statistics for these three parameterizations are displayed in Table S6, showing that model fits are similar, with the model including efficacy as a main effect having slightly better RMSE. Figure S6 shows a plot of the residuals from the model excluding efficacy against the efficacy covariate, showing a relationship.

| **Parameterization** | **R^2^** | **RMSE** |
| --- | --- | --- |
| **Efficacy** |  |  |
| Fixed and Random Effects | 0.961 | 0.607 |
| Fixed Effects only | 0.944 | 0.738 |
| **Effective coverage** |  |  |
| Fixed and Random Effects | 0.956 | 0.646 |
| Fixed Effects only | 0.936 | 0.798 |
| **Efficacy excluded** |  |  |
| Fixed and Random Effects | 0.960 | 0.613 |
| Fixed Effects only | 0.940 | 0.760 |
| **Table S6.** Fit statistics for selecting efficacy parameter | | |

| 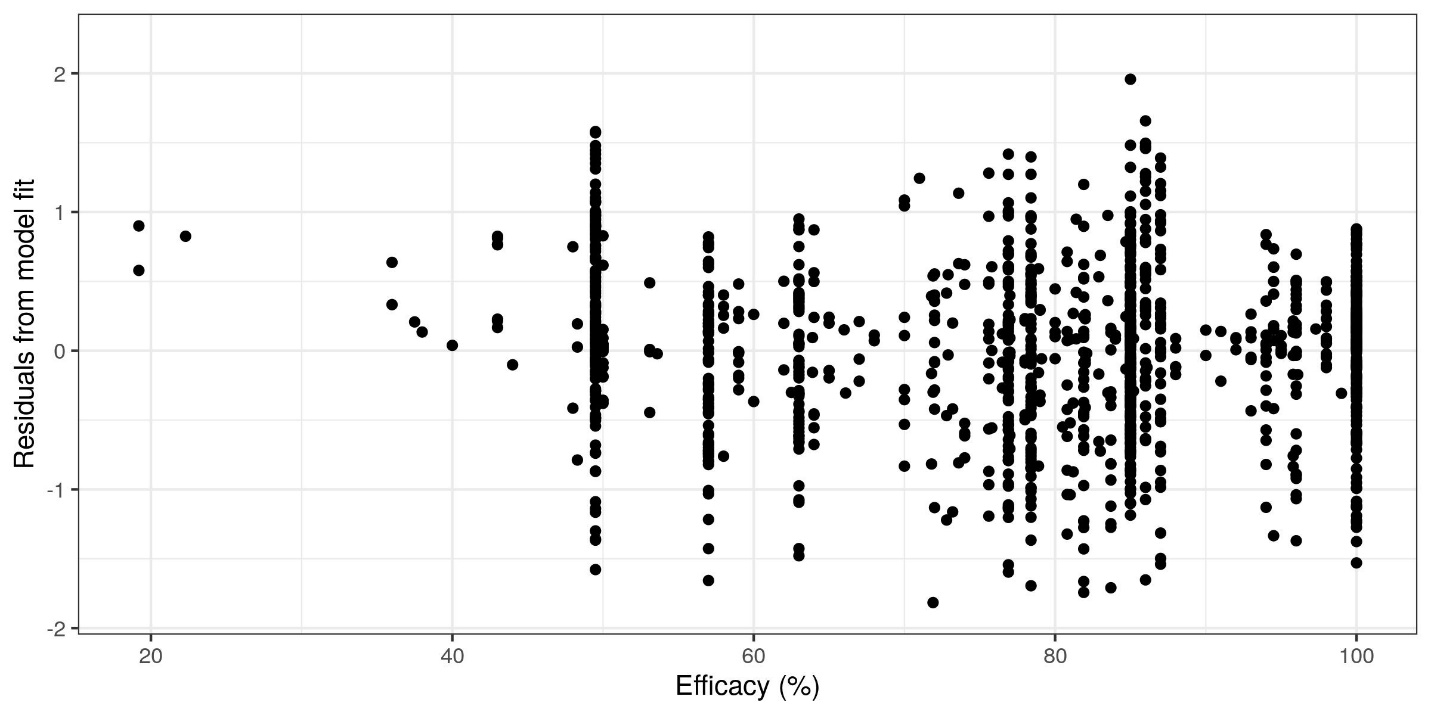 |
| --- |
| **Figure S6.** Residuals from model without efficacy plotted against vaccine efficacy reported in published studies. |

## Section 5 References

[1] Rosettie KL, Joffe JN, Sparks GW, Aravkin A, Chen S, Compton K, et al. Cost-effectiveness of HPV vaccination in 195 countries: A meta-regression analysis. PLOS ONE 2021;16:e0260808

[2] Zheng P, Barber R, Sorensen RJD, Murray CJL, Aravkin AY. Trimmed Constrained Mixed Effects Models: Formulations and Algorithms. Journal of Computational and Graphical Statistics 2021;30:544–56. https://doi.org/10.1080/10618600.2020.1868303.

[3] de Boor C. A Practical Guide to Spline. vol. Volume 27. 1978. https://doi.org/10.2307/2006241.

[4] Friedman JH. Multivariate Adaptive Regression Splines. The Annals of Statistics 1991;19:1–67.

[5] Aravkin A, Davis D. Trimmed Statistical Estimation via Variance Reduction. Mathematics of OR 2020;45:292–322. https://doi.org/10.1287/moor.2019.0992.

[6] Rousseeuw, P. J. Multivariate estimation with high breakdown point. Mathematical Statistics and Applications 1985;8:283–97.

[7] Murray CJL, Aravkin AY, Zheng P, Abbafati C, Abbas KM, Abbasi-Kangevari M, et al. Global burden of 87 risk factors in 204 countries and territories, 1990–2019: a systematic analysis for the Global Burden of Disease Study 2019. The Lancet 2020;396:1223–49. https://doi.org/10.1016/S0140-6736(20)30752-2.

[8] Motzkin TS, Raiffa H, Thompson GL, Thrall RM. 3. The Double Description Method. 3. The Double Description Method, Princeton University Press; 2016, p. 51–74. https://doi.org/10.1515/9781400881970-004.

[9] Bondell HD, Krishna A, Ghosh SK. Joint Variable Selection for Fixed and Random Effects in Linear Mixed-Effects Models. Biometrics 2010;66:1069–77. https://doi.org/10.1111/j.1541-0420.2010.01391.x.

[10] Müller S, Scealy JL, Welsh AH. Model Selection in Linear Mixed Models. Statistical Science 2013;28:135–67. https://doi.org/10.1214/12-STS410.

[11] Fu WJ. Penalized Regressions: The Bridge versus the Lasso. Journal of Computational and Graphical Statistics 1998;7:397–416. https://doi.org/10.1080/10618600.1998.10474784.

[12] Zou H, Hastie T. Regularization and variable selection via the elastic net. Journal of the Royal Statistical Society: Series B (Statistical Methodology) 2005;67:301–20. https://doi.org/10.1111/j.1467-9868.2005.00503.x.

[13] Zheng P, Askham T, Brunton SL, Kutz JN, Aravkin AY. A Unified Framework for Sparse Relaxed Regularized Regression: SR3. IEEE Access 2019;7:1404–23. https://doi.org/10.1109/ACCESS.2018.2886528.

Section 6: Cost-saving predictions

We built a logistic regression model with both fixed and random effects to predict the probability of a rotavirus vaccine intervention being cost-saving. The model was trained on data from the Tuft’s registries (1439 ratios from 68 articles). In order to account for between study heterogeneity, ratios were grouped by article and a random intercept was calculated for each article.

We built a model that included the same covariates as the meta-regression analysis. These fixed covariates were: GDP per capita, cost of a full vaccine series (in 2017 USD), vaccine coverage (%), cost discount rate (%), burden discount rate (QALY or DALY discount rate [%]), rotavirus DALYs per person (burden), time horizon (lifetime or less than lifetime), vaccine type (monovalent, pentavalent, or both [monovalent reference]), outcome measure (either DALYs or QALYs).

We log transformed GDP per capita, DALYs per person, and vaccine cost to correct for a right skewed distribution of both of these variables. Results of the logistic regression model, including estimates of the coefficients for all covariates, along with standard error, Z value and p value are in **Table S7**. Of all the covariates used in the model, log GDP per capita, log vaccine cost, DALY/QALY discount rate, target sex, and log cervical burden were statistically significant.

| **Table S7.** Logistic regression output for cost-saving predictions. | | | | |
| --- | --- | --- | --- | --- |
| **Variable** | **Estimate** | **Standard error** | **Z value** | **P value** |
| Intercept | -14.55 | 2.81 | -5.179 | <0.0001 |
| Log GDP per capita | 1.31 | 0.285 | 4.585 | <0.0001 |
| Log vaccine cost (2017 USD) | -1.112 | 0.224 | -4.965 | <0.0001 |
| Coverage | 0.002 | 0.020 | 0.112 | 0.911 |
| Cost discount rate | -0.146 | 0.226 | -0.649 | 0.516 |
| DALY/QALY discount rate | 0.219 | 0.228 | 0.963 | 0.336 |
| Burden Measure (QALYs; ref=DALYs) | 0.376 | 1.005 | 0.374 | 0.708 |
| Payer Perspective | -2.268 | 0.563 | -4.030 | <0.0001 |
| Efficacy | 0.077 | 0.021 | 3.630 | 0.0003 |
| Log burden variable | 0.544 | 0.169 | 3.218 | 0.0013 |
| Vaccine type (Pentavalent; ref = monovalent) | -0.610 | 0.377 | -1.615 | 0.106 |
| Vaccine type (both types; ref=monovalent) | -3.197 | 1.570 | -2.036 | 0.041 |

# Section 7. Vaccine Cost

Vaccine cost is a covariate in the meta-regression model, and we need a variable for vaccine cost for each country to predict its ICER. We used the Rotavirus vaccine per dose as reported to the WHO’s Market Information for Access to Vaccines (MI4A)[1] and aggregated by Linksbridge [2]. Linksbridge reports five categories of prices per dose for the Rotavirus vaccine in 2017. (**Table S8)**. Vaccine cost was defined as the cost to completely vaccinate one person with a monovalent vaccine in 2017. For example, the vaccine cost was US$4.61 ($2.31 x 2 doses) for countries that were eligible for GAVI support in 2020.

| **Table S8**. **Vaccine cost for estimating incremental cost-effectiveness ratios for 195 countries** | | |
| --- | --- | --- |
| **Category** | **Definition** | **2017 price per course in US$** |
| United States | Price from the US Centers for Disease Control and Prevention | 150.25 |
| High income countries other than the United States | Countries classified as high income by the World Bank | 41.37 |
| Upper-middle income countries | Countries classified as upper-middle income by the World Bank that are not members of PAHO or eligible for UNICEF | 22.53 |
| Lower-middle income countries | Countries classified as lower-middle income by the World Bank that are not members of PAHO or eligible for UNICEF | Only 3 countries in this category were not eligible for PAHO and UNICEF, and we used the price for upper middle income countries |
| Pan American Health Organization (PAHO) | Countries eligible for the PAHO Revolving Fund for supported countries and vaccines | 13.00 |
| United Nations Children’s Fund (UNICEF) | UNICEF as price paid for purchase made for GAVI-supported countries and vaccines | 4.61 |

Linksbridge reports vaccine cost data without adjusting prices to the currency year of the most recent almanac. We use 2017 vaccine cost, which would be in 2017 US$.

We also explored fitting a regression model to the vaccine cost data reported in published CEA, but decided that the Linksbridge results are more representative. The vaccine cost in the published CEA are below US$7.76 per for half of the published estimates using in our analysis, including many middle and high-income countries. **Figure S7** shows the relationship between vaccine cost and gross domestic product (GDP) per capita on a log scale for both sources of vaccine information: 1) published articles in the Tufts registries, and 2) Linksbridge. The low vaccine cost in the published articles is not a problem for the metaregression analyses, because the published ICERs are correspondingly low. They are a problem for predicting vaccine cost, because models don’t fit these data well, and vaccine cost is not representative of market prices.


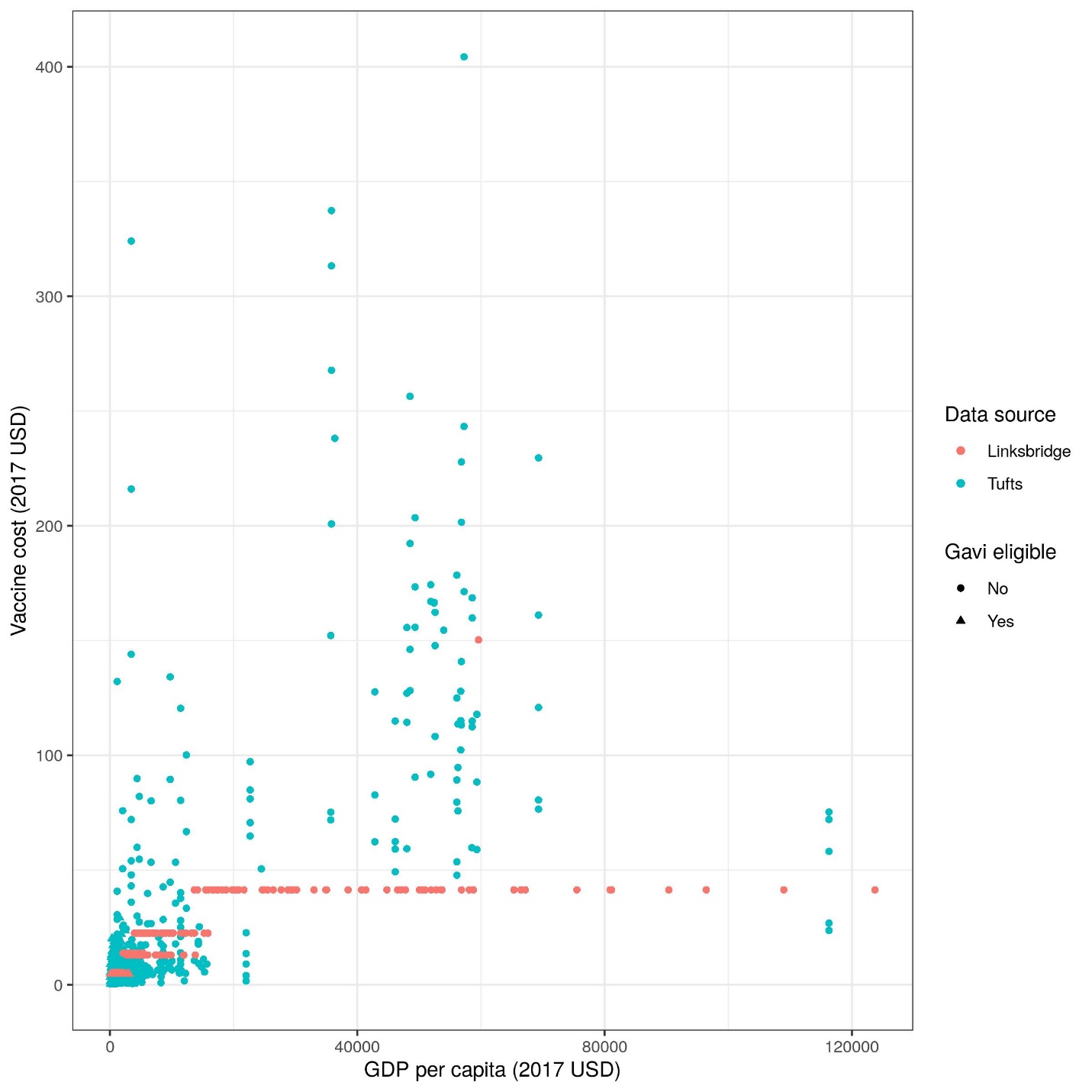


**Figure S7: Scatterplot of vaccine cost versus GDP per capita from sources of vaccine information: 1) published articles in the Tufts registries, and 2) Linksbridge**

A limitation of the Linksbridge results, however, is the low response rate among high income countries on the MI4A reporting. For example in 2019, 41% of high income countries reported complete data, compared to 72% of countries globally [3]. The Mi4A data are more representative for other categories.

## Section 7 References

[1] World Health Organization,. MI4A vaccine purchase data for countries. 2018.

[2] Linksbridge. Vaccine Almanac. 2021.

[3] World Health Organization,. Global vaccine market report. 2018.

Section 8. Adjusted incremental cost-effectiveness ratios predicted from the model excluding efficacy

| **Table S9. Predicted incremental cost-effectiveness ratios by country adjusted for cost-saving probabilities from the model without efficacy** | | | | | |
| --- | --- | --- | --- | --- | --- |
| **Country** | **Predicted ICER adjusted for cost-saving probabilities in 2017 US$ per DALY Averted (95% UI)** | **Rotavirus diarrhea DALYs per 100,000 children 0 to 5 years of age** | **Tufts registry dataset and additional extractions** | | |
|  |  |  | **Number of ratios** | **Minimum ICER in 2017 US$ per DALY or QALY** | **Maximum ICER in 2017 US$ per DALY or QALY** |
| Central Europe Eastern Europe and Central Asia |  |  |  |  |  |
| Albania | 3,243  (502 – 11,259) | 84.0 | 4 | 446 | 12,964 |
| Armenia | 3,792 (586 - 13,163) | 52.1 | 16 | 20 | 5,630 |
| Azerbaijan | 2,609  (404 – 8,932) | 249.5 | 9 | 9 | 100 |
| Belarus | 4,202  (650 - 14,499) | 62.6 | 2 | 2,989 | 3,626 |
| Bosnia and Herzegovina | 3,154  (488 - 10,886) | 114.7 | 2 | 6,160 | 6,949 |
| Bulgaria | 3,424  (530 - 11,662) | 170.7 | 2 | 4,584 | 6,370 |
| Croatia | 8,333 (1,281 - 28,253) | 105.8 | 0 | NA | NA |
| Czech Republic | 10,519  (1,614 - 35,490) | 110.0 | 0 | NA | NA |
| Estonia | 12,279  (1,884 - 41,609) | 55.6 | 0 | NA | NA |
| Georgia | 2,895  (449 - 10,016) | 52.0 | 10 | 61 | 4,274 |
| Hungary | 9,412  (1,446 - 31,893) | 89.4 | 0 | NA | NA |
| Kazakhstan | 5,422  (837 - 18,385) | 87.6 | 2 | 585 | 689 |
| Kyrgyzstan | 505  (77 - 1,761) | 228.1 | 10 | 22 | 685 |
| Latvia | 8,953  (1,376 - 30,350) | 93.9 | 0 | NA | NA |
| Lithuania | 8,215  (1,263 - 27,788) | 134.8 | 0 | NA | NA |
| Macedonia | 2,653  (411 - 9,158) | 167.0 | 2 | 5,866 | 6,849 |
| Moldova | 1,518  (236 - 5,316) | 117.3 | 10 | 839 | 5,272 |
| Mongolia | 1,373 (213 - 4,712) | 368.6 | 10 | 27 | 370 |
| Montenegro | 4,577  (707 - 15,715) | 64.4 | 0 | NA | NA |
| Poland | 7,515  (1,156 - 25,449) | 142.1 | 0 | NA | NA |
| Romania | 3,403  (526 - 11,514) | 248.0 | 2 | 2,163 | 4,570 |
| Russian Federation | 4,416  (682 - 14,920) | 177.6 | 0 | NA | NA |
| Serbia | 3,647  (564 - 12,559) | 92.5 | 0 | NA | NA |
| Slovakia | 8,726  (1,341 - 29,459) | 145.7 | 0 | NA | NA |
| Slovenia | 11,487  (1,764 - 38,711) | 105.7 | 0 | NA | NA |
| Tajikistan | 180  (28 - 623) | 3,114.2 | 10 | 2 | 185 |
| Turkmenistan | 3,258  (504 - 11,119) | 173.1 | 0 | NA | NA |
| Ukraine | 2,173  (337 - 7,584) | 66.9 | 10 | 1,641 | 4,783 |
| Uzbekistan | 1,075  (164 - 3,724) | 58.5 | 6 | 1 | 42 |
| High-Income |  |  |  |  |  |
| Andorra | 22,733  (3,514 – 77,292) | 46.2 | 0 | NA | NA |
| Argentina | 6,215  (957 - 21,073) | 178.9 | 14 | 95 | 21,814 |
| Australia | 31,739  (4,893 – 106,943) | 36.5 | 11 | 2,765 | 68,147 |
| Austria | 22,568  (3,498 – 76,725) | 52.5 | 0 | NA | NA |
| Belgium | 20,888  (3,236 - 71,150) | 58.4 | 61 | 5,630 | 119,778 |
| Brunei | 18,353  (2,831 - 62,737) | 59.6 | 0 | NA | NA |
| Canada | 30,457  (4,705 – 102,824) | 27.9 | 14 | 2,078 | 117,187 |
| Chile | 7,561  (1,162 - 25,555) | 168.2 | 7 | 2,237 | 35,200 |
| Cyprus | 15,171  (2,325 - 51,189) | 56.0 | 0 | NA | NA |
| Denmark | 17,231  (2,644 - 58,853) | 141.4 | 0 | NA | NA |
| Finland | 19,980  (3,093 - 68,117) | 68.2 | 30 | 4,820 | 151,315 |
| France | 19,143  (2,964 - 65,349) | 65.4 | 46 | 18,843 | 249,924 |
| Germany | 19,816  (3,069 - 67,574) | 67.7 | 8 | 80,059 | 209,020 |
| Greece | 18,885  (2,893 - 64,029) | 23.9 | 0 | NA | NA |
| Greenland | 16,218  (2,497 - 55,538) | 121.7 | 0 | NA | NA |
| Iceland | 23,283  (3,604 – 79,106) | 47.2 | 0 | NA | NA |
| Ireland | 37,446  (5,787 - 125,674) | 26.7 | 5 | 59,838 | 206,479 |
| Israel | 18,787  (2,894 – 64,192) | 54.8 | 12 | 3,284 | 96,391 |
| Italy | 23,539  (3,599 – 80,030) | 27.8 | 0 | NA | NA |
| Japan | 25,454  (3,943 -86,287) | 42.1 | 4 | 7,527 | 85,246 |
| Luxembourg | 35,839  (5,483 - 120,020) | 50.3 | 0 | NA | NA |
| Malta | 18,937  (2,899 – 63,981) | 32.9 | 0 | NA | NA |
| Netherlands | 23,537  (3,640 – 79,920) | 53.1 | 57 | 3,770 | 168,172 |
| New Zealand | 22,243  (3,426 - 75,691) | 41.0 | 2 | 39,221 | 57,018 |
| Norway | 22,723  (3,477 – 76,818) | 128.0 | 2 | 51,044 | 56,705 |
| Portugal | 17,839  (2,733 – 60,458) | 27.6 | 0 | NA | NA |
| Singapore | 24,704  (3,808 – 83,750) | 55.9 | 0 | NA | NA |
| South Korea | 17,275  (2,646 - 58,341) | 40.5 | 8 | 107 | 354 |
| Spain | 18,857  (2,888 - 64,370) | 42.2 | 10 | 26,761 | 319,574 |
| Sweden | 21,709  (3,335 - 73,791) | 80.6 | 0 | NA | NA |
| Switzerland | 24,872  (3,849 – 84,079) | 91.5 | 0 | NA | NA |
| United Kingdom | 36,818  (5,619 – 124,166) | 11.7 | 44 | 39,730 | 189,763 |
| Uruguay | 7,012  (1,078 - 23,713) | 182.6 | 2 | 1,470 | 1,525 |
| USA | 58,718  (9,164 – 197,602) | 45.5 | 2 | 1,470 | 1,525 |
| Latin America and Caribbean |  |  |  |  |  |
| Antigua and Barbuda | 7,492  (1,152 - 25,395) | 130.7 | 0 | NA | NA |
| Barbados | 9,528 (1,464 - 32,290) | 86.5 | 0 | NA | NA |
| Belize | 1,366 (212 - 4,699) | 310.5 | 2 | 544 | 621 |
| Bermuda | 29,813  (4,640 - 100,474) | 54.6 | 0 | NA | NA |
| Bolivia | 896  (139 - 3,104) | 562.1 | 10 | 25 | 388 |
| Brazil | 2,459  (381 - 8,284) | 277.7 | 9 | 856 | 7,223 |
| Colombia | 2,039  (316 - 6,907) | 245.8 | 4 | 892 | 2,461 |
| Costa Rica | 2,643  (410 - 8,931) | 183.8 | 2 | 5,186 | 5,313 |
| Cuba | 2,892  (448 - 9,902) | 72.9 | 8 | 6,674 | 17,604 |
| Dominica | 1,763  (273 - 6,000) | 276.8 | 0 | NA | NA |
| Dominican Republic | 1,676  (260 - 5,679) | 367.4 | 7 | 494 | 1,036 |
| Ecuador | 1,930  (299 – 6,619) | 163.1 | 0 | NA | NA |
| El Salvador | 1,196  (185 - 4,122) | 377.8 | 0 | NA | NA |
| Grenada | 3,010  (467 - 10,212) | 99.4 | 0 | NA | NA |
| Guatemala | 542  (84 - 1,861) | 2,533.1 | 2 | 662 | 906 |
| Guyana | 960  (149 - 3,294) | 741.3 | 10 | 3 | 2,973 |
| Haiti | 193  (30 - 666) | 2,110.5 | 10 | 2 | 174 |
| Honduras | 832  (129 – 2,889) | 598.4 | 15 | 51 | 1,089 |
| Jamaica | 2,147  (333 - 7,391) | 110.3 | 2 | 925 | 925 |
| Mexico | 2,433 (377 - 8,214) | 230.1 | 10 | 589 | 3,232 |
| Nicaragua | 511  (78 - 1,767) | 330.7 | 10 | 100 | 1,106 |
| Panama | 4,021  (620 - 13,588) | 531.2 | 7 | 70 | 3,295 |
| Paraguay | 1,858  (288 - 6,403) | 144.2 | 2 | 1,477 | 1,615 |
| Peru | 3,065  (475 - 10,505) | 62.0 | 20 | 132 | 2,438 |
| Puerto Rico | 14,327  (2,196 - 48,360) | 64.8 | 0 | NA | NA |
| Saint Lucia | 2,537  (393 - 8,603) | 144.9 | 2 | 2,567 | 2,568 |
| Saint Vincent and The Grenadines | 1,816  (282 - 6,189) | 247.2 | 0 | NA | NA |
| Suriname | 1,371  (213 - 4,653) | 536.4 | 0 | NA | NA |
| The Bahamas | 12,547  (1,925 - 42,371) | 89.4 | 0 | NA | NA |
| Trinidad and Tobago | 7,854  (1,207 - 26,532) | 165.3 | 0 | NA | NA |
| Venezuela | 1,381  (214 – 4,678) | 575.2 | 5 | 622 | 1,540 |
| Virgin Islands | 16,562 (2,538 - 56,369) | 54.1 | 0 | NA | NA |
| North Africa and Middle East |  |  |  |  |  |
| Afghanistan | 160  (25 - 553) | 3,324.8 | 13 | 5 | 82 |
| Algeria | 2,449  (379 - 8,435) | 218.5 | 2 | 828 | 1,070 |
| Bahrain | 12,245  (1,878 - 41,317) | 82.7 | 0 | NA | NA |
| Egypt | 594  (92 - 2,055) | 1,646.9 | 3 | 424 | 553 |
| Iran | 2,765  (428 - 9,395) | 300.9 | 10 | 3 | 10,224 |
| Iraq | 2,334  (362 - 7,998) | 301.2 | 2 | 219 | 270 |
| Jordan | 2,146  (333 - 7,460) | 196.8 | 2 | 1,842 | 1,873 |
| Kuwait | 13,427  (2,080 - 46,222) | 130.2 | 0 | NA | NA |
| Lebanon | 2,984  (462 - 10,168) | 225.1 | 0 | NA | NA |
| Libya | 2,936  (454 - 10,113) | 146.2 | 1 | 8,411 | 8,411 |
| Morocco | 915  (142 - 3,158) | 711.8 | 2 | 672 | 871 |
| Oman | 6,855  (1,054 - 23,119) | 244.8 | 1 | 2,462 | 2,462 |
| Palestine | 1,536  (239 – 5,365) | 137.0 | 0 | NA | NA |
| Qatar | 26,850  (4,155 – 90,754) | 61.1 | 0 | NA | NA |
| Saudi Arabia | 8,822  (1,358 - 29,697) | 184.2 | 0 | NA | NA |
| Sudan | 274 (42 - 935) | 2,206.7 | 10 | 62 | 316 |
| Syria | 686  (104 - 2,391) | 118.3 | 2 | 720 | 753 |
| Tunisia | 2,101   (326 - 7,248) | 117.2 | 2 | 1,387 | 1,412 |
| Turkey | 5,796  (894 - 19,617) | 92.8 | 2 | 596 | 1,319 |
| United Arab Emirates | 15,641  (2,416 - 53,629) | 112.6 | 0 | NA | NA |
| Yemen | 143  (23 - 495) | 4,085.9 | 10 | 18 | 408 |
| South Asia |  |  |  |  |  |
| Bangladesh | 300  (46 - 1,038) | 829.8 | 19 | 23 | 1,543 |
| Bhutan | 1,335  (207 – 4,638) | 236.1 | 10 | 15 | 280 |
| India | 256  (40 - 879) | 1,981.5 | 29 | 17 | 294 |
| Nepal | 408  (63 - 1,429) | 298.7 | 10 | 23 | 776 |
| Pakistan | 320  (49 - 1,107) | 791.4 | 15 | 15 | 371 |
| Southeast Asia East Asia and Oceania |  |  |  |  |  |
| American Samoa | 3,372  (521 - 11,458) | 195.0 | 0 | NA | NA |
| Cambodia | 299  (46 - 1,035) | 869.3 | 9 | 1 | 99 |
| China | 3,176  (491 - 10,855) | 173.9 | 35 | 688 | 213,789 |
| Federated States of Micronesia | 1,434  (223 - 4,986) | 192.0 | 2 | 960 | 1,057 |
| Fiji | 1,510  (234 - 5,192) | 692.1 | 2 | 1,971 | 2,179 |
| Guam | 9,731  (1,506 - 33,527) | 236.8 | 0 | NA | NA |
| Indonesia | 666  (103 - 2,271) | 2,402.1 | 13 | 30 | 468 |
| Kiribati | 622  (97 - 2,166) | 1,121.5 | 8 | 4 | 153 |
| Laos | 170  (27 - 584) | 5,772.2 | 10 | 6 | 111 |
| Malaysia | 4,586  (708 - 15,539) | 127.7 | 2 | 2,488 | 2,899 |
| Maldives | 3,927  (607 - 13,371) | 127.7 | 2 | 19,039 | 23,812 |
| Marshall Islands | 1,970  (306 - 6,856) | 225.3 | 0 | NA | NA |
| Mauritius | 3,337  (516 - 11,285) | 264.7 | 1 | 418,802 | 418,802 |
| Myanmar | 237  (37 - 815) | 2,107.3 | 10 | 7 | 238 |
| North Korea | 305  (47 - 1,057) | 741.2 | 0 | NA | NA |
| Northern Mariana Islands | 7,707  (1,185 - 26,015) | 183.9 | 0 | NA | NA |
| Papua New Guinea | 261  (40 - 890) | 2,501.9 | 8 | 20 | 181 |
| Philippines | 728  (113 - 2,511) | 1,221.6 | 2 | 404 | 446 |
| Samoa | 2,247  (348 - 7,794) | 196.7 | 2 | 1,527 | 1,698 |
| Seychelles | 7,187  (1,106 - 24,364) | 140.7 | 0 | NA | NA |
| Solomon Islands | 368  (56 - 1,271) | 662.8 | 10 | 168 | 990 |
| Sri Lanka | 2,380  (369 - 8,256) | 70.7 | 10 | 190 | 4,144 |
| Taiwan (Province Of China) | 10,779  (1,654 - 36,350) | 109.3 | 5 | 308 | 7,903 |
| Thailand | 2,405  (373 - 8,235) | 293.7 | 3 | 126 | 4,335 |
| Timor-Leste | 654  (102 - 2,251) | 1,617.6 | 10 | 19 | 302 |
| Tonga | 2,432  (377 - 8,410) | 187.2 | 2 | 2,869 | 3,170 |
| Vanuatu | 865  (134 - 2,989) | 768.7 | 2 | 2,878 | 3,254 |
| Vietnam | 1,201  (187 - 4,208) | 205.3 | 29 | 82 | 3,548 |
| Sub-Saharan Africa |  |  |  |  |  |
| Angola | 472  (73 - 1,611) | 5,893.8 | 8 | 0 | 107 |
| Benin | 128  (20 - 441) | 6,770.6 | 10 | 11 | 134 |
| Botswana | 1,194  (185 - 4,041) | 2,682.2 | 2 | 298 | 459 |
| Burkina Faso | 103  (16 -357) | 10,865.4 | 10 | 9 | 99 |
| Burundi | 128 (20 - 443) | 3,689.4 | 10 | 11 | 103 |
| Cameroon | 164  (26 - 565) | 4,953.5 | 10 | 10 | 87 |
| Cape Verde | 1,083  (168 - 3,739) | 492.2 | 2 | 1,041 | 1,373 |
| Central African Republic | 64  (10 - 223) | 27,445.5 | 10 | 14 | 117 |
| Chad | 81  (13 - 280) | 23,156.9 | 9 | 7 | 47 |
| Comoros | 190  (30 - 656) | 2,673.6 | 10 | 71 | 210 |
| Congo (Brazzaville) | 212  (33 - 725) | 4,290.1 | 11 | 17 | 149 |
| Cote D'Ivoire | 164   (26 - 562) | 5,289.2 | 10 | 10 | 184 |
| Djibouti | 252  (39 - 869) | 1,768.6 | 7 | 17 | 121 |
| DR Congo | 101  (16 - 349) | 9,297.0 | 10 | 15 | 106 |
| Equatorial Guinea | 1,632  (253 – 5,508) | 2,480.6 | 0 | NA | NA |
| Eritrea | 142   (22 - 492) | 4,185.5 | 10 | 72 | 635 |
| Ethiopia | 144 (23 -499) | 3,995.0 | 11 | 13 | 145 |
| Gabon | 1,373  (213 - 4,638) | 2,426.4 | 0 | NA | NA |
| Ghana | 211  (33 - 721) | 3147.3 | 17 | 2 | 519 |
| Guinea | 135  (21 - 468) | 5116.5 | 10 | 8 | 80 |
| Guinea-Bissau | 112  (18 - 388) | 8,414.6 | 10 | 4 | 70 |
| Kenya | 197  (31 - 680) | 2,547.8 | 25 | 22 | 553 |
| Lesotho | 177  (28 - 609) | 4,207.3 | 10 | 184 | 790 |
| Liberia | 131  (21 - 452) | 4,966.2 | 10 | 10 | 144 |
| Madagascar | 132  (21 - 458) | 4,728.8 | 10 | 7 | 206 |
| Malawi | 173  (27 - 599) | 2,354.5 | 25 | 2 | 194 |
| Mali | 192  (30 - 665) | 2,326.7 | 10 | 2 | 60 |
| Mauritania | 199   (31 - 684) | 3,046.7 | 10 | 8 | 75 |
| Mozambique | 178  (28 - 615) | 2,327.8 | 10 | 13 | 98 |
| Namibia | 1,211   (188 - 4,125) | 1,643.8 | 2 | 650 | 819 |
| Niger | 95  (15 - 328) | 10,655.5 | 10 | 5 | 63 |
| Nigeria | 129  (20 - 446) | 14,002.9 | 11 | 1 | 74 |
| Rwanda | 159  (25 - 551) | 3,653.4 | 10 | 0 | 127 |
| Sao Tome and Principe | 303  (47 - 1,046) | 902.0 | 10 | 27 | 385 |
| Senegal | 170  (27 - 587) | 3,732.1 | 24 | 15 | 174 |
| Sierra Leone | 126  (20 - 438) | 5,362.5 | 10 | 1 | 71 |
| Somalia | 86  (14 - 301) | 7,146.1 | 10 | 4 | 30 |
| South Africa | 1,594  (247 - 5,399) | 1,114.7 | 2 | 124 | 186 |
| South Sudan | 108  (17 - 374) | 14,259.4 | 0 | NA | NA |
| Swaziland | 467  (73 - 1,597) | 5,669.8 | 2 | 222 | 302 |
| Tanzania | 291  (45 - 1,010) | 790.3 | 11 | 16 | 160 |
| The Gambia | 257  (40 - 895) | 836.8 | 10 | 20 | 160 |
| Togo | 129  (20 - 447) | 5,594.9 | 10 | 20 | 216 |
| Uganda | 175  (28 - 606) | 2,580.0 | 13 | 11 | 80 |
| Zambia | 212  (33 - 727) | 2,967.4 | 10 | 10 | 118 |
| Zimbabwe | 182  (29 - 629) | 2,773.1 | 8 | 18 | 246 |
| **Legend to Table S9.** Country predictions assuming 90% vaccine coverage, lifetime time horizon, 3% cost and burden discount rates, monovalent vaccine type, DALYs averted as health outcome measure, vaccine cost (for 2-dose course) and no intervention as the comparator. ICER=incremental cost-effectiveness ratio, UI=uncertainty interval, QALY=quality-adjusted life-year, DALY=disability adjusted life-year | | | | | |
